# MCM3 upregulation confers endocrine resistance in breast cancer and is a predictive marker of diminished tamoxifen benefit

**DOI:** 10.1038/s41523-020-00210-8

**Published:** 2021-01-04

**Authors:** Sanne Løkkegaard, Daniel Elias, Carla L. Alves, Martin V. Bennetzen, Anne-Vibeke Lænkholm, Martin Bak, Morten F. Gjerstorff, Lene E. Johansen, Henriette Vever, Christina Bjerre, Tove Kirkegaard, Bo Nordenskjöld, Tommy Fornander, Olle Stål, Linda S. Lindström, Laura J. Esserman, Anne E. Lykkesfeldt, Jens S. Andersen, Rikke Leth-Larsen, Henrik J. Ditzel

**Affiliations:** 1 Department of Cancer and Inflammation Research, Institute of Molecular Medicine, University of Southern Denmark, Odense, DK-5000 Denmark; 2 Center of Experimental Bioinformatics, Department of Biochemistry and Molecular Biology, University of Southern Denmark, Odense, DK-5230 Denmark; 3 Department of Surgical Pathology, Zealand University Hospital, Roskilde, DK-4000 Denmark; 4 Department of Pathology, Odense University Hospital, Odense, DK-5000 Denmark; 5 Department of Oncology, Copenhagen University Hospital, Rigshospitalet DK-2100 Copenhagen, Denmark; 6 Cell Death and Metabolism, Danish Cancer Society Research Center, Copenhagen, DK-2100 Denmark; 7 Department of Clinical and Experimental Medicine, Division of Oncology, Linköping University, Linköping, SE-58185 Sweden; 8 Department of Oncology, Karolinska University Hospital, Stockholm, SE-11883 Sweden; 9 Department of Biosciences and Nutrition, Karolinska Institutet, Stockholm, SE-14183 Sweden; 10 Department of Surgery, UCSF Carol Franc Buck Breast Care Center, University of California, San Francisco, San Francisco, 94115 CA USA; 11 Department of Regional Health Research, University of Southern Denmark, Odense, DK-5000 Denmark; 12 Department of Oncology, Odense University Hospital; Department of Clinical Research, University of Southern Deanmark, Odense, DK-5000 Denmark; 13 Academy of Geriatric Cancer Research (AgeCare), Odense University Hospital, Odense, DK-5000 Denmark

**Keywords:** Predictive markers, Mechanisms of disease

## Abstract

Resistance to endocrine therapy in estrogen receptor-positive (ER^+^) breast cancer is a major clinical problem with poorly understood mechanisms. There is an unmet need for prognostic and predictive biomarkers to allow appropriate therapeutic targeting. We evaluated the mechanism by which minichromosome maintenance protein 3 (MCM3) influences endocrine resistance and its predictive/prognostic potential in ER^+^ breast cancer. We discovered that ER^+^ breast cancer cells survive tamoxifen and letrozole treatments through upregulation of minichromosome maintenance proteins (MCMs), including MCM3, which are key molecules in the cell cycle and DNA replication. Lowering MCM3 expression in endocrine-resistant cells restored drug sensitivity and altered phosphorylation of cell cycle regulators, including p53(Ser^315,33^), CHK1(Ser^317^), and cdc25b(Ser^323^), suggesting that the interaction of MCM3 with cell cycle proteins is an important mechanism of overcoming replicative stress and anti-proliferative effects of endocrine treatments. Interestingly, the MCM3 levels did not affect the efficacy of growth inhibitory by CDK4/6 inhibitors. Evaluation of MCM3 levels in primary tumors from four independent cohorts of breast cancer patients receiving adjuvant tamoxifen mono-therapy or no adjuvant treatment, including the Stockholm tamoxifen (STO-3) trial, showed MCM3 to be an independent prognostic marker adding information beyond Ki67. In addition, MCM3 was shown to be a predictive marker of response to endocrine treatment. Our study reveals a coordinated signaling network centered around MCM3 that limits response to endocrine therapy in ER^+^ breast cancer and identifies MCM3 as a clinically useful prognostic and predictive biomarker that allows personalized treatment of ER^+^ breast cancer patients.

## Introduction

Approximately 80% of breast cancers express the estrogen-receptor (ER^+^)^1^, rendering them suitable for adjuvant anti-estrogen treatment. In ER^+^ breast cancer patients, adjuvant anti-estrogen treatment with aromatase inhibitors (AIs) and tamoxifen significantly reduced the risk of recurrence and death in all age groups studied^2^. In addition to the adjuvant setting, anti-estrogen treatments are also effective in the metastatic setting and, although not curative, extend survival. In postmenopausal women with ER^+^ breast cancer, AIs are superior to tamoxifen^3^. However, the side-effects of the two types of drugs differ and some patients may not be eligible for AI treatment due to co-morbidities^3^. Tamoxifen is therefore maintained as an adjuvant treatment option for postmenopausal ER^+^ breast cancer patients. Further, tamoxifen is a recommended treatment modality for premenopausal breast cancer patients. Although tamoxifen is of great benefit for many ER^+^ breast cancer patients, recurrence occurs in approximately 30% over 15-years of follow-up^2^.

Tamoxifen is a selective estrogen receptor modulator (SERM) with both antagonistic and agonistic tissue-dependent effects. In vitro, tamoxifen acts as a partial estrogen antagonist, by antagonizing the estrogen regulation of the transcription of most ER-regulated genes and inhibiting growth of estrogen receptor-dependent breast cancer cells^4–6^. Tamoxifen resistance has been studied in vitro and at the clinical level. Loss of ERα expression during adjuvant tamoxifen therapy has been observed in approximately 20% of recurring patients^7,8^, however, tamoxifen resistance can be acquired with intact functional ER signaling, since studies have shown that patients with acquired tamoxifen resistance may benefit from fulvestrant or AIs^9–11^.

The minichromosome maintenance 3 protein (MCM3) protein belongs to a family of 6 highly conserved minichromosome maintenance proteins (MCM2-MCM7) that are essential to ensure eukaryotic DNA is replicated only once per cell cycle, and additionally acts as a helicase to drive replication elongation. In late M1 phase, cdt1 and cdc6 recruits a heterohexamer complex of MCM2-7, which is loaded on to the origin of replication forming the pre-replication complex. Activation of the pre-replication complex occurs by cdc7-, cdc45-, DBF4- and S-phase cyclin-dependent kinases along G1-S phase transition^12^.

In this study, quantitative proteomic analysis revealed upregulation of MCM3 in tamoxifen- and AI-resistant breast cancer cells and knockdown of MCM3 resensitized these cells to tamoxifen or letrozole and resulted in altered phosphorylation of cell cycle regulator proteins. The clinical relevance of MCM3 expression as a biomarker was demonstrated in primary ER^+^ breast cancers of 4 large well-characterized cohorts of ER^+^ breast cancer patients.

## Results

### Quantitative proteomic analysis reveals altered protein expression levels in tamoxifen-resistant vs. parental breast cancer cell lines

**Fig. 1:**
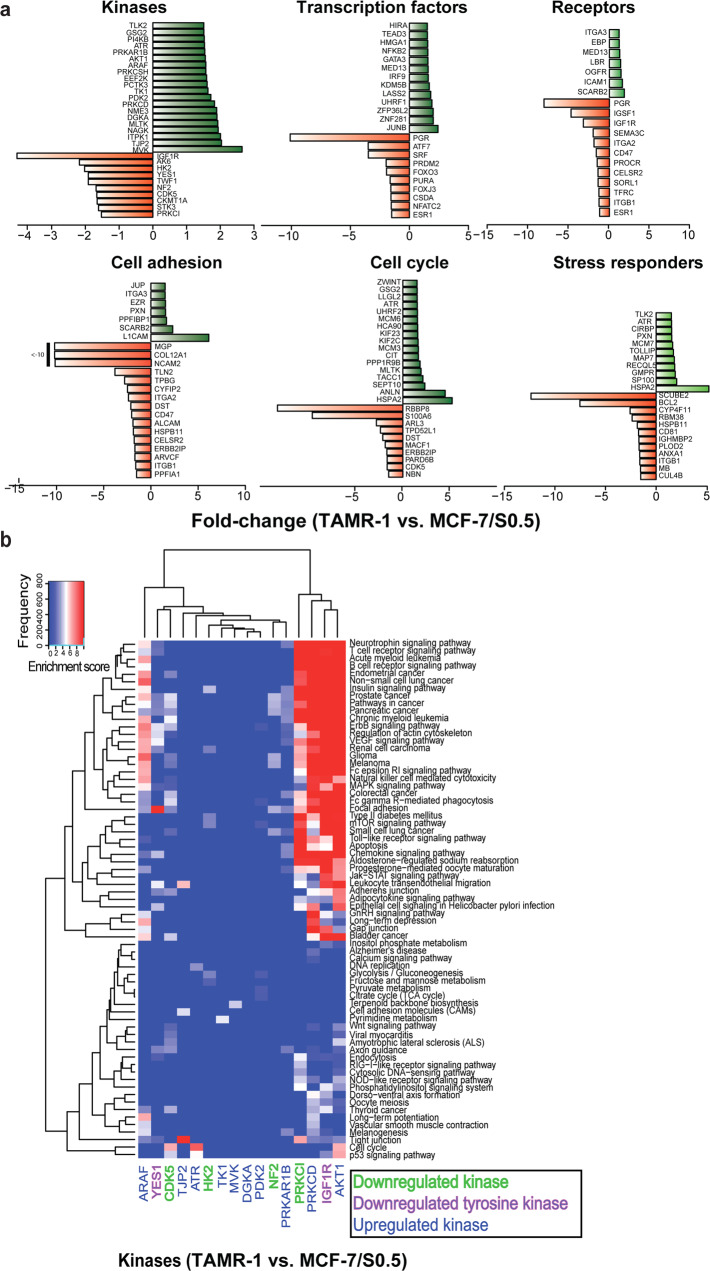
Protein expression in tamoxifen-resistant cells by quantitative proteomic analysis. (**a**) Proteins exhibiting altered expression levels above 1.5-fold in TamR-1 vs. MCF-7/S0.5 were sub-grouped into kinase-, transcription factor-, receptor-, cell adhesion-, cell cycle and stress response proteins. (**b**) Heatmap of pathways associated with subnetworks centered around each of the 16 kinases from larger significant protein networks (STRING score > 0.7) among the regulated kinases shown in (**a**). A global functional association network was constructed using the probabilistic algorithm STRING based on identified proteins exhibiting altered expression by >1.5-fold. Subsequently, networks of direct neighbors for each regulated kinase were extracted and subjected to KEGG pathway analysis. using the bioinformatical tool DAVID by applying hypergeometric enrichment testing (Fisher’s Exact test) and subsequent adjustment for multiple testing using the standard Benjamini-Hochberg (BH) algorithm to obtain an adjusted *p*-value. The significance level was set to 0.05 after BH-adjustment. Finally, an enrichment score was defined as –log10(P). Color key indicates the pathways enrichment score. Kinases central to each subnetwork are shown as expressed in TamR-1 vs. MCF-7/S0.5 and listed as the column names in the heatmap. Upregulated kinases are shown in blue, downregulated kinases in green, and downregulated tyrosine kinases in purple. The pathways as listed as the row names in the heatmap.

To identify protein and pathways alterations associated with tamoxifen resistance, the proteomes of parental tamoxifen-sensitive MCF-7/S0.5 and tamoxifen-resistant TamR-1 cell lines were compared using SILAC-labeling and quantitative LC-MS/MS. The mass spectrometry data identified 4,448 proteins (>two unique peptides, FDR of 1%), of which 539 exhibited significant altered expression in TamR-1 vs. MCF-7/S0.5 cells (Supplementary Table 1). The 539 proteins with altered expression could be sub-grouped into kinases, transcription factors, receptor proteins, cell adhesion proteins, cell cycle proteins and stress response proteins (Fig. 1a). Among the kinases, expression of AKT1, PDK2, and A-Raf were higher, while proto-oncogenes YES1 and IGF-1R, were lower in TamR-1 vs. MCF-7/S0.5 (Fig. 1a, b). Among transcription factors, KDM5B, JUNB, and NFKB2 showed increased expression, while FOXO3 and FOXJ3 were expressed at a lower level in TamR-1 vs. MCF-7/S0.5 (Fig. 1a). In agreement with previous studies, we found lower ERα (1.5-fold) and severely reduced (10-fold) expression of PGR in TamR-1 vs. MCF-7/S0.5^6,13^. Among the cell cycle and stress response proteins, the conserved family of DNA replication licensing factor MCM complex proteins, including MCM3, −6 and −7 and the DNA damage-response serine/threonine kinase ATR, exhibited higher expression, while the cell cycle regulatory protein p95 (NBS1) and CtIP exhibited reduced expression in TamR-1 vs. MCF-7/S0.5 cells (Fig. 1a). The altered expression of selected proteins from each sub-group, including AKT1, IGF-1R, JUNB, FOXO3a, ERα, ATR, and CtIP, was validated by Western blotting (Supplementary Fig. 1). Sixteen of the 30 protein kinases exhibiting altered expression in TamR-1 vs. MCF-7/S0.5 could be associated with different pathways of which AKT1, IGF1R, and PRKCD were all involved in pathways such as MAPK-, ErbB-, and insulin signaling pathways (Fig. 1b).

### DNA replication and cell cycle functions are enriched in tamoxifen-resistant breast cancer cells

Protein-protein interaction network analysis of the 539 proteins exhibiting altered expression was performed using the STRING database (Supplementary Fig. 2a). MCODE analysis of the global network extracted five highly connected subnetworks (Supplementary Figs 2b and 3a). Deciphering the functional subnetworks with KEGG pathway analysis showed that DNA replication and cell cycle functions were greatly enriched among the regulated proteins (Supplementary Figs. 2b and 3b). Interestingly, subnetwork 1 included the family of MCM proteins, including MCM3, MCM4, MCM5, MCM6 and MCM7, and the serine/threonine kinase ATR, that were all more highly expressed in TamR-1 vs. MCF-7/S0.5 (Supplementary Fig. 3a, c) and involved in DNA replication and cell cycle functions.

### MCM3 expression is associated with short recurrence-free and overall survival in early-stage, ER^+^ breast cancer patients treated with tamoxifen mono-therapy

**Fig. 2:**
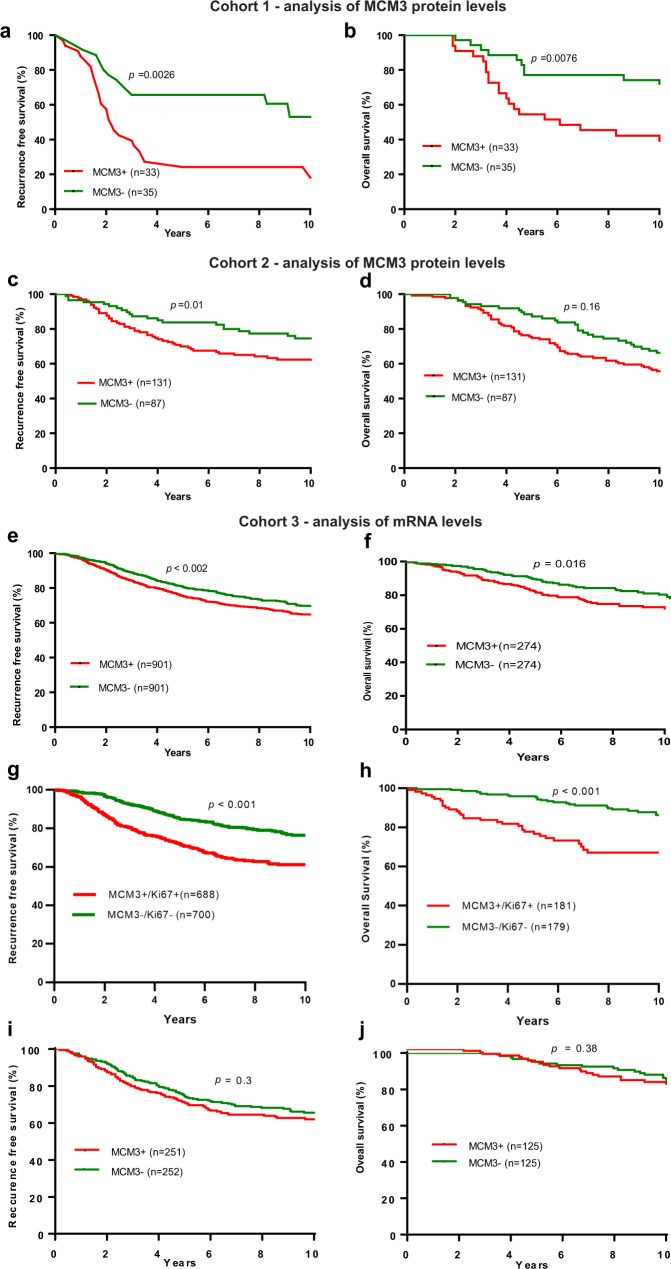
Association of MCM3 expression with clinical outcome in endocrine treated-patients. Kaplan-Meier plots showing the association of MCM3 expression with 10-year recurrence-free survival (RFS) and overall survival (OS) in ER^+^, adjuvant endocrine treated patients in cohort 1 (**a**, **b**) that consisted of 79 ER^+^ breast cancer patients who received 5 years of adjuvant tamoxifen monotherapy, and in cohort 2 (**c**, **d**) that consisted of 218 post and perimenopausal ER^+^ breast cancer patients who received adjuvant tamoxifen monotherapy assessed by immunohistochemistry. The association of MCM3 expression with PFS and OS in cohort 3 (**e**, **f**) that consisted of 1802 adjuvant endocrine treated ER^+^ breast cancer patients where MCM3 expression was assessed by gene array. The prognostic significance of the combination of MCM3/Ki67 in ER^+^ breast cancer patients as demonstrated by correlation to disease progression (**g**) or death (**h**) in patients included in cohort 3 using Cox regression analysis. Evaluation of the association of MCM3 mRNA expression with PFS (**i**) and OS (**j**) in the 503 ER^+^ breast cancer patients in cohort 3 who did not receive adjuvant endocrine or chemotherapy showed no significant correlation between MCM3 expression and clinical outcome.

To explore whether MCM expression levels were associated with clinical outcome of tamoxifen treatment, we evaluated MCM3 expression by immunohistochemistry in three independent cohorts ER^+^ breast cancer patients. In addition, we evaluated MCM3 expression at the mRNA level in a large cohort (cohort 3) of ER^+^ breast cancer patients treated with endocrine therapy (*n* = 1802), ER^+^ patients who did not receive any systemic therapy (*n* = 503) as well as ER^−^ breast cancer patients (*n* = 250). The first cohort consisted of postmenopausal patients with early ER^+^ breast cancers (*n* = 79) who received adjuvant tamoxifen mono-therapy for 5 years (Supplementary Table 2) of which 68 had sufficient tumor tissue for MCM3 staining. Evaluation of MCM3 staining showed that its expression was limited to the nucleus of tumor cells, while other cells in the tumor such as lymphocytes and stromal cells were generally negative (Supplementary Fig. 4). The analysis showed that patients with MCM3-positive (MCM3^+^) tumors were significantly more likely to develop recurrence and die than patients with MCM3-negative (MCM3^−^) tumors (Fig. 2a, b). Multivariate analysis showed that MCM3 expression was independent of tumor size, lymph node status, tumor grade, and age in its association with RFS (*p* < 0.003) and OS (*p* < 0.008) (Table 1).

**Table 1:** Correlation of selected clinical parameters and MCM3 expression with clinical outcome in the 3 cohorts of breast cancer patients where MCM3 expression was measured by immunohistochemistry.

	Cohort 1				Cohort 2				Cohort 4			
	RFS		OS		RFS		OS		RFS		BCSS	
Clinical parameter	*P* value	HR (95% CI)	*P* value	HR (95% CI)	*P* value	HR (95% CI)	*P* value	HR (95% CI)	*P* value	HR (95% CI)	*P* value	HR (95% CI)
MCM3	0.0026	2.7 (1.4–5.3)	0.007	2.5 (1.3–4.6)	0.01	2.0 (1.2–3.3)	0.16	1.4 (0.89–1.89)	0.033	2.5 (1.1–5.7)	0.044	2.9 (1.0–8.1)
Lymph node	0.002	2.4 (1.4–4.2)	0.002	2.5 (1.4–4.6)	0.0001	2.6 (1.7–4.0)	0.0001	2.1 (1.5–3.0)	NA	–	NA	–
Tumor size	0.4	0.9 (0.6–1.4)	0.9	0.9 (0.6–1.5)	0.005	1.7 (1.2–2.4)	0.002	1.6 (1.2–2.1)	0.7	1.2 (0.5–2.6)	0.065	2.4 (0.9–5.9)
Age	0.9	0.9 (0.9–1.0)	0.7	1.0 (0.9–1.1)	0.87	1.0 (0.96–1.03)	0.02	1.2 (1.0–1.05)	0.3	0.7 (0.3–1.4)	0.7	0.8 (0.3–2.1)
Tumor grade	0.012	3.4 (1.3–8.9)	0.03	2.6 (1.1–6.6)	0.6	1.1 (0.85–1.3)	0.8	1.02 (0.85–1.2)	0.1	1.6 (0.8–3.3)	0.3	1.6 (0.7–3.8)

Variables were analyzed as follows: MCM3 (+ vs. −), tumor size (<20 mm vs. >20 mm), tumor grade (1 vs. 2 and 3), nodal status (+ vs. −), age (< or = 59 vs. > 59). 95% CI (95% confidence interval). Bold data represent significant values. *RFS* relapse-free survival, *OS* Overall survival, *BCSS* breast cancer-specific survival.

Subsequently, MCM3 expression was evaluated in the second cohort consisting of 218 postmenopausal patients with high-risk, early-stage, ER^+^ breast cancers, who had received adjuvant tamoxifen mono-therapy (Supplementary Table 2). MCM3^+^ tumors were significantly associated with poor 10-year RFS compared to MCM3^−^ tumors. However, the association to OS did not reach statistical significance in this cohort (Fig. 2c, d). Multivariate analysis revealed that MCM3 expression in this cohort was an independent prognostic factor associated with a shorter RFS (*p* = 0.01) (Table 1).

Next, we analyzed the correlation between MCM3 mRNA expression and outcome in a large cohort of 1802 endocrine-treated ER^+^ breast cancer patients that received adjuvant endocrine therapy, ER^+^ breast cancer patients that did not receive adjuvant therapy (*n* = 504 for PFS and *n* = 250 for OS) as well as ER^−^ patients (*n* = 249 for PFS and *n* = 104 for OS) available through KM plotter 2014 version^14^. In agreement with the MCM3 analysis by immunohistochemistry, the gene expression data showed that endocrine treated patients with primary tumors exhibiting high MCM3 mRNA level were significantly more likely to develop recurrence (HR 1.3, 95%CI 1.13–1.56, *p* = 0.0019) and die (HR 1.7, 95%CI 1.1–2.3, *p* = 0.016) than patients with low MCM3 mRNA level (Fig. 2e, f). In contrast, no correlation between MCM3 mRNA expression and clinical outcome in ER^−^ breast cancer patients was observed using mRNA data in KM plotter (data not shown).

**Fig. 3:**
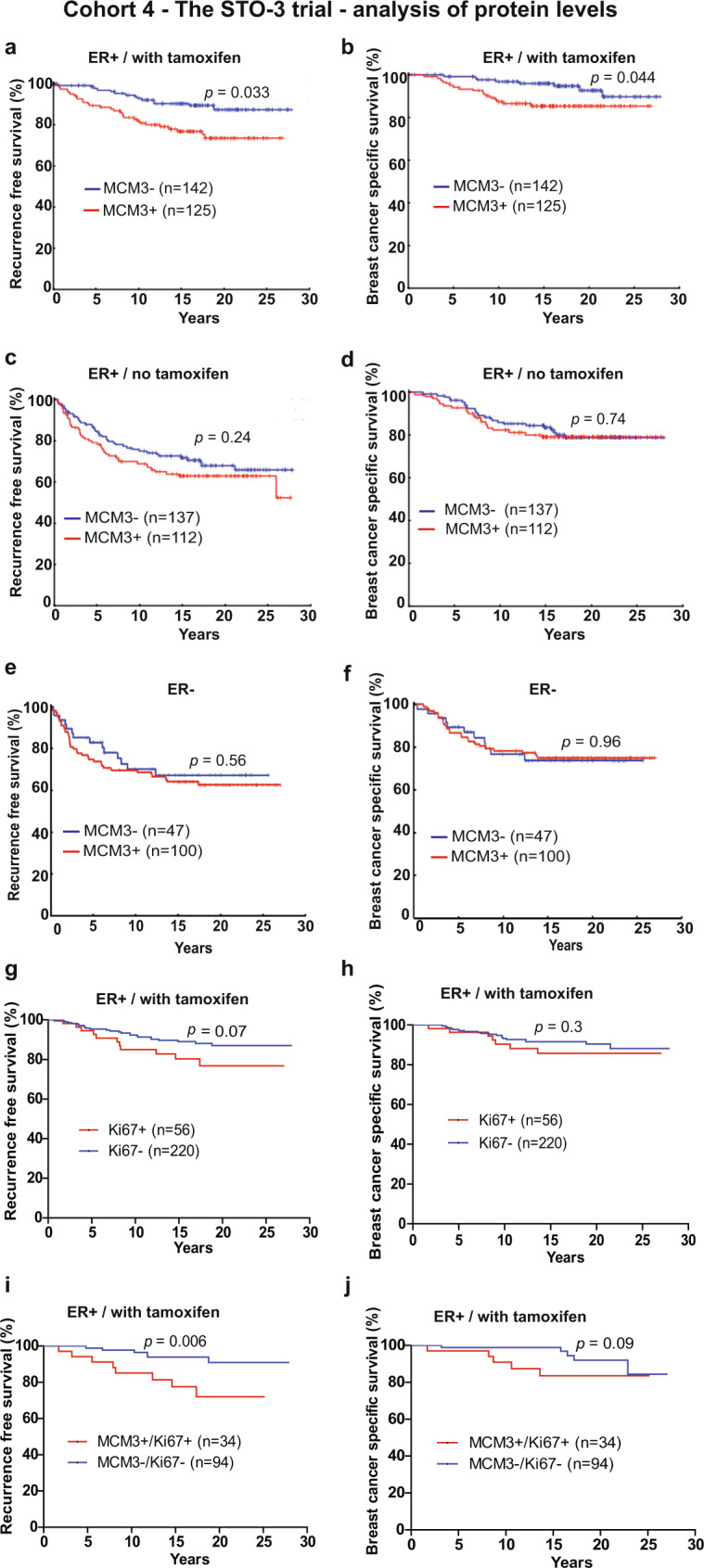
Association of MCM3 expression with clinical outcome in tamoxifen-treated patients. Kaplan-Meier plots showing the association of MCM3 expression with recurrence-free survival (RFS) and breast cancer-specific survival (BCSS) in cohort 4 consisting of adjuvant tamoxifen-treated or -untreated postmenopausal, high-risk, ER^+^ breast cancer patients from the STO-3 trial. In ER^+^ adjuvant tamoxifen-treated patients, MCM3^+^ expression was significantly associated with a shorter RFS (**a**) and BCSS (**b**) compared to MCM3^−^ tumors. However, in adjuvant untreated patients, MCM3 expression was not significantly associated with RFS (**c**) or BCSS (**d**). Moreover, in the ER^−^ subgroup of this cohort, MCM3 expression did not significantly correlate with RFS (**e**) or BCSS (**f**). Ki67 expression was evaluated in the tamoxifen-treated patient population and shown not to significantly correlate to RFS (**g**) or BCSS (**h**). Combining MCM3 expression with Ki67 significantly improved correlation to RFS (**i**), but not BCSS (**j**). The log-rank (Mantel-Cox) test was used to determine statistical significance.

Next, we evaluated whether MCM3 is a predictive marker of adjuvant tamoxifen treatment benefit. The MCM3 level was examined by immunohistochemistry in the Stockholm Tamoxifen (STO-3) trial from the Stockholm Breast Cancer Study Group randomizing postmenopausal lymph node-negative breast cancer patients to receive adjuvant tamoxifen versus not^15^. MCM3 expression was evaluated in 683 accessible tumors, of which 516 (78%) were ER^+^ and 147 (22%) were ER^−^ (Supplementary Table 2). Of the 516 ER^+^ patients, 267 (51.7%) were treated with adjuvant tamoxifen. In this subgroup, MCM3^+^ tumors were detected in 125 of 267 (47%) ER^+^ patients. These patients had a significantly shorter RFS than those with MCM3^−^ tumors (Fig. 3a), supporting the findings in the first 3 cohorts. Interestingly, patients with MCM3^+^ tumors also had a significantly shorter breast cancer-specific survival (BCSS) compared to patients with MCM3^−^ tumors (Fig. 3b). Multivariate analysis revealed that MCM3 expression was an independent prognostic factor associated with RFS (*p* = 0.033) and a BCSS (*p* = 0.044) in ER^+^ patients in cohort 4 (Table 1).

### MCM3 is a predictive marker of adjuvant tamoxifen treatment benefit in early-stage ER^+^ breast cancer patients

To determine whether MCM3 had a predictive potential in addition to its prognostic value, the association of MCM3 expression and clinical outcome was investigated in 2 cohorts of ER^+^ breast cancer patients that did not receive adjuvant endocrine or chemotherapy; one based on gene array data obtained from KMplot.com and the second based on immunohistochemical evaluated of this subgroup of the STO-3 cohort. Interestingly, no significant correlation between MCM3 expression and RFS (*p* = 0.3, or *p* = 0.24) or breast cancer-specific death (*p* = 0.34 or *p* = 0.74) was observed for neither of the two cohorts (Fig. 2i, j and Fig. 3c, d), suggesting that MCM3 has potential as a predictive marker that can stratify ER^+^ patients into good and poor responders of adjuvant tamoxifen treatment. Further analysis showed that MCM3 was not significantly associated with RFS or breast cancer-specific death in the ER^−^ patient subgroup in the STO-3 cohort (Fig. 3e, f) and similar observation was made in ER^−^ patients obtained from the KM plotter data (data not shown), which suggests that the prognostic and predictive value of MCM3 is restricted to the ER^+^ breast cancer subgroup.

### Comparison of the prognostic potential of MCM3 and Ki67 in ER^+^ breast cancer patients who received adjuvant endocrine therapy

Since the MCM3 protein is involved in cell proliferation and is consistently associated with outcome of ER^+^ breast cancer patients who received adjuvant endocrine therapy, we questioned whether the prognostic potential of MCM3 expression is affected by Ki67, a molecule widely used as a proliferative marker. Ki67 is a clinically used marker to distinguish high versus low proliferative tumors, which is especially important for ER^+^ breast cancers. Univariate analysis showed that Ki67 expression is significantly correlated to clinical outcomes in cohorts 1, 2, and 3, but not in cohort 4 (Table 2, Fig. 3g–j). For cohort 3 we further investigated whether the prognostic potential of MCM3 can be affected by Ki67 expression levels by performing multivariate analysis using Cox proportional hazards model with MCM3 and Ki67 as co-variates. The result showed that the 2 markers independently correlate to RFS (MCM3: HR 1.32, 95% CI 1.11–1.58, *p* = 0.002; Ki67: HR 1.27 95*%* CI 1.05–1.55, *p* = 0.013). MCM3 was independent of Ki67 in its correlation to OS (HR 1.67, 95% CI 1.12–2.5, *p* = 0.012). However, the correlation of Ki67 to OS was not significant (*p* = 0.8) when controlling for MCM3 expression, consistent with the observation in cohort 4, where Ki67 failed to show significant correlation to clinical outcome (Fig. 3g, h). This suggests that MCM3 expression is a more consistent and reliable marker of clinical outcome in ER + breast cancer patients than Ki67. Taken together, the results suggest that MCM3, in addition to being a predictive marker of tamoxifen benefit in ER^+^ breast cancer, is a more consistent prognostic marker in ER^+^ breast cancer than Ki67, and may add information to clinical decision-making beyond that of Ki67.

**Table 2:** Correlation between Ki67 expression levels and clinical outcome in 4 independent cohorts of tamoxifen-treated ER^+^ breast cancer patients.

Cohort	Progression free survival		Overall survival (or BCSS^a^)	
	HR (95% CI)	*P* value	HR (95% CI)	*P* value
Cohort 1	2.8 (1.48–5.3)	0.003	2.4 (1.2–4.8)	0.012
Cohort 2	1.8 (1.05–2.9)	0.03	1.7 (1.1–2.7)	0.011
Cohort 3	1.5 (1.3–1.8)	<0.0001	2.4 (1.7–3.5)	<0.0001
Cohort 4	2.1 (0.93–4.97)	0.07	1.7 (0.6–4.4)	0.30

^a^For cohort 4 breast cancer-specific survival (BCSS) was considered the clinical end-point, while in cohorts 1, 2, and 3, death irrespective of cause was considered the clinical endpoint.

### Reduction of MCM3 protein expression restored tamoxifen and AI sensitivity in resistant cells

**Fig. 4:**
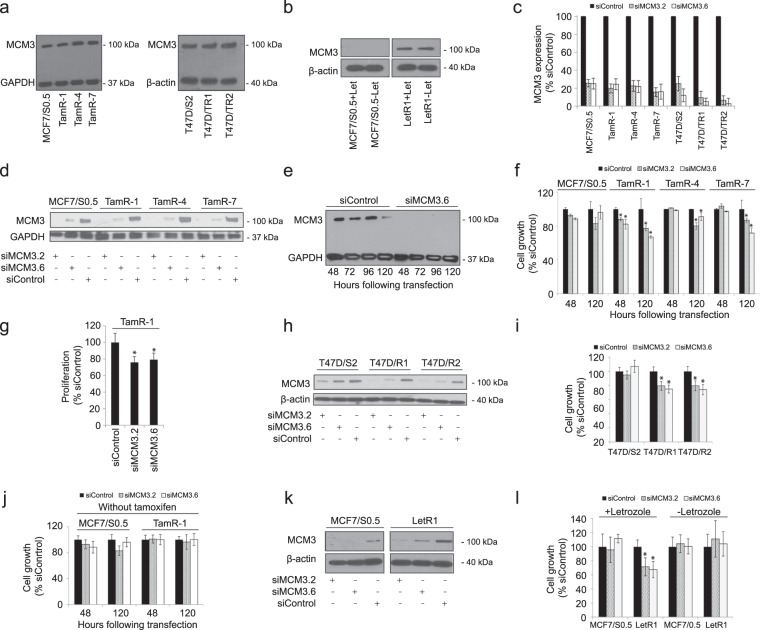
Resistant cells regain sensitivity to tamoxifen and letrozole following MCM3 knockdown. Increased MCM3 protein levels in tamoxifen- or aromatase inhibitor-resistant vs. -sensitive cell lines was confirmed by Western blotting using whole cell lysate in (**a**) two tamoxifen-resistant models, one MCF-7-based (MCF-7/S0.5 vs. TamR-1, TamR-4 and TamR-7) and one T47D-based (T47D/S2 vs. T47D/TR-1 and T47D/TR-2) and in (**b**) a letrozole-resistant model (MCF-7/S0.5 vs. LetR1). Transfection of cells with 2 separate MCM3 targeting siRNAs (siMCM3.2 and siMCM3.6) resulted in > 75% reduction in the expression of MCM3 compared to a scrambled siRNA (siControl) used as a control, as measured by RT-qPCR at 48 h (**c**) and Western blotting at 96 h (**d**) or at 48, 72, 96 and 120 h (**e**). Data are representive of three independent experiments. (**f**) Knockdown of MCM3 by either of the two siRNAs in TamR cells treated with 10^−6^M tamoxifen significantly decreased growth compared to siControl transfected cells measured by a colorimetric crystal violet assay at 120 h. The reduction in cell growth of MCM3 knockdown was confirmed in three independent experiments. Data is represented as OD590 values ± s.e.m of triplicates. (**g**) Cell proliferation as measured by BrdU incorporation demonstrated significantly lower proliferation of TamR-1 cells transfected with siMCM3.2 and siMCM3.6 compared to siControl-transfected cells. Similarly, transfection of the tamoxifen-resistant cell lines T47D/S2, T47D/TR-1, and T47D/TR-2 with two separate siRNAs targeting MCM3 lead to 70–90% reduction of MCM3 levels vs. siControl, as measured by RT-qPCR at 48 h (**c**) and by Western blotting at 96 h (**h**), and significantly reduced growth of T47D/TR-1 and T47D/TR-2 in the presence of 10^−6^M tamoxifen (**i**) as measured by a colorimetric crystal violet assay. In contrast, the growth of the tamoxifen-sensitive parental cell lines MCF-7/S0.5 (**f**) and T47D/S.2 (**i**) or tamoxifen-resistant TamR-1 cells cultured in the absence of tamoxifen (**j**) transfected with siMCM3 was not significantly reduced compared to cells transfected with siControl. A representative of three independent experiments in which data is represented by OD590 values ± s.e.m in triplicates is shown, *p < 0.05. Transfection of the letrozole-resistant cell line LetR1 vs. MCF-7/S0.5 with two separate siRNAs targeting MCM3 resulted in significant reduction of MCM3 levels vs. siControl as measured by Western blotting at 96 h (**k**), and significantly reduced growth of LetR1 in the presence, but not in the absence, of letrozole (**l**), as measured by a colorimetric crystal violet assay at 120 h. *p < 0.05. PUM1 was used as a reference gene in the RT-qPCR and β-actin or GAPDH was used as loading control for Western blotting.

Based on the important clinical data on MCM3 as a prognostic marker and a predictive biomarker for tamoxifen responsiveness, we examined the underlying mechanism by which MCM3 confers endocrine resistance. Initially, the higher MCM3 level in tamoxifen-resistant vs. parental cells was confirmed by Western blotting (Fig. 4a and Supplementary Fig. 5a) and found to be independent of the growth rate (Supplementary Fig. 5b). Higher MCM3 level was also observed in T47D-derived tamoxifen-resistant (T47D/R) cells vs. parental cells (T47D/S2) (Fig. 4a and Supplementary Fig. 5a) and in AI-resistant (letrozole) cell line (LetR1) vs. parental cells (Fig. 4b). We also found increased MCM3 level (1.5–1.7 fold) in MCF-7 cells cultured 6–10 months in estrogen-deprived medium, referred to as long-term estrogen-deprived (LTED) cells vs. those cultured at normal conditions (Supplementary Table 3). In contrast, MCM3 level was not increased in the fulvestrant-resistant cell line (FulvR-1) compared to the parental cells (MCF-7/S0.5) (Supplementary Fig. 5c).

The effect of MCM3 expression on growth of the different MCF-7-derived tamoxifen-resistant cell lines treated with tamoxifen was studied by MCM3 silencing using two different siRNAs, siMCM3.2 and siMCM3.6. MCM3 knockdown resulted in significantly reduced growth of TamR-1 (*p* ≤ 0.037), TamR-4 (*p* = 0.013), and TamR-7 (*p* ≤ 0.048) in tamoxifen-containing medium as measured by colorimetric crystal violet assay, but did not significantly affect the growth of tamoxifen-sensitive parental MCF-7/S0.5 cells, indicating that up-regulation of MCM3 is important for the resistant phenotype (Fig. 4f). MCM3 knockdown also reduced proliferation as determined by a BrdU incorporation assay, as demonstrated for TamR-1 (Fig. 4g). Together this suggests that increased MCM3 expression protects tamoxifen-resistant cell lines against tamoxifen-induced growth inhibition.

To confirm that these observations were not restricted to one cell line model, MCM3 was also silenced in two T47D-derived tamoxifen-resistant (Fig. 4c, h) and the letrozole-resistant LetR1 cell lines (Fig. 4k). Lowering MCM3 expression led to significantly reduced growth of T47D/TR-1 (*p* = 0.033) and T47D/TR-2 (*p* = 0.008) cells in tamoxifen-containing medium (Fig. 4i and Supplementary Fig. 5d) and LetR1 cells (*p* = 0.001) in letrozole-containing medium (Fig. 4l and Supplementary Fig. 5e). Importantly, MCM3 knockdown in parental cells did not significantly inhibit growth (Fig. 4i, l). Neither did MCM3 knockdown in the resistant cells in absence of tamoxifen or letrozole (Fig. 4j, l), suggesting that the importance of MCM3 to the growth and survival is dependent on the ER pathway.

**Fig. 5:**
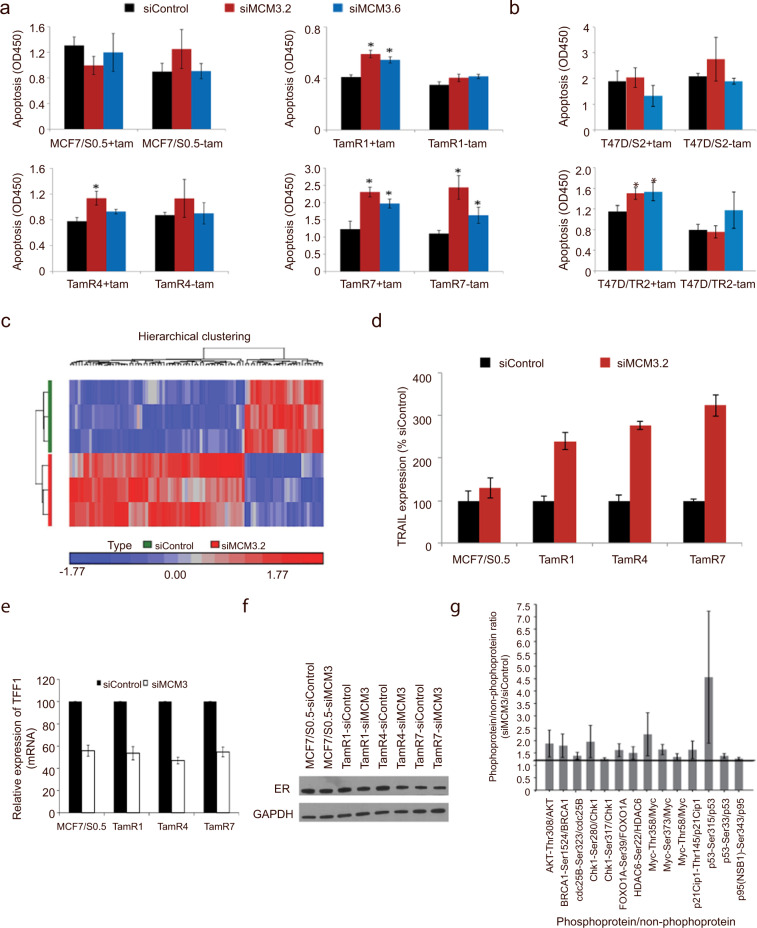
Reduction of MCM3 levels enhances apoptosis and reduces ER-transactivation. Apoptotic cell death following siRNA-mediated knockdown of MCM3 was analyzed in two different tamoxifen-resistant breast cancer cell line models, (**a**) one MCF-7-based consisting of 3 tamoxifen-resistant TamR-1, TamR-4 and TamR-7, and the parental (MCF-7/S0.5) cells and (**b**) one T47D-based consisting of one tamoxifen-resistant (T47D/TR2) and the parental (T47D/S2) cells. Apoptosis was measured by ELISA at 96 h following transfection with two different siRNAs targeting MCM3 (siMCM3.2 and siMCM3.6) and control siRNA (siControl) in the presence (+tam) or absence (-tam) of 10^−6^M tamoxifen. *p < 0.05. A representative of three independent experiments, each performed in triplicate, is shown. (**c**) Heatmap showing the effect of MCM3 knockdown on gene expression in TamR cell lines as evaluated using Affymetrix HG-U133 plus 2 gene array. Data analysis was performed using Partek Genomic Suite. Raw Affymetrix intensity measurements were normalized and summarized into gene expression measurements using Robust Multiarray Average. Levels of (**d**) TRAIL, (**e**) the estrogen-regulated gene TFF1 (pS2) and (**f**) estrogen receptor (ER) in MCF7/S0.5 and TamR cell lines following MCM3 gene knockdown (comparing siMCM3.2- vs. siControl-transfected cells) as determined by RT-qPCR or Western blotting. PUM1 was used as a reference gene in the RT-qPCR and GAPDH used as Western blot loading control. (**g**) Phosphorylation alterations of 14 cell cycle regulator proteins in TamR cells following MCM3 knockdown as determined by protein array. The bold line indicates a 1.2-fold regulation. Fold-changes are the mean values of 3 biological experiments.

Next, the effect on MCM3 knockdown on apoptosis was evaluated in the MCF7- and T47D-based tamoxifen-resistant and corresponding parental cells (Fig. 5a, b) using camptothecin treated or untreated cells as a positive (signal intensity of 1.7) and negative control (signal intensity of 0.011) respectively. Knockdown of MCM3 resulted in significant enhancement of tamoxifen-induced apoptotic cell death in tamoxifen-resistant breast cancer cell lines (Fig. 5a, b), while no significant difference was observed in the absence of tamoxifen, with the exception of TamR-7. Furthermore, no significant change in apoptosis was observed following MCM3 knockdown in the parental cell lines.

To address the underlying mechanism of the decreased cell growth and increased apoptosis in endocrine-resistant cells, we performed global gene array analysis of TamR-1, TamR-4, TamR-7, and parental MCF/S0.5 cells following siRNA MCM3 knockdown or transfection with siControl using Affymetrix HG-U133 plus 2 arrays. To identify the genes that were functionally related to growth inhibition in TamR cells following MCM3 knockdown, we focused on those genes that were regulated in TamR, but not in the parental MCF7/S0.5 cells, transfected with MCM3-specific siRNAs. This analysis led to the identification of 77 genes with altered expressions (FDR < 0.05 and ≥ 1.5 fold altered expression) (Fig. 5c, Supplementary Table 4) and enrichment analysis using Ingenuity Pathway Analysis (IPA) revealed that the genes were associated with cell signaling, cell death and survival, and cell cycle (Supplementary Table 5). The two top networks of genes exhibiting altered expression are provided in Supplementary Fig. 6. Analysis of the MCM3-regulated genes unique to TamR showed that TRAIL, a key inducer of apoptosis, was upregulated upon MCM3 knockdown, as confirmed by RT-qPCR (Fig. 5d). Among growth and proliferation-related genes, EGR3 and JAM-A (F11R) were downregulated and STAT1 upregulated following MCM3 knockdown in TamR cells. Finally, two tumor suppressors, CDKN2B and TSLC1, were upregulated upon MCM3 knockdown in TamR cells.

The importance of the ER pathway in mediating the effect of MCM3 on growth and survival in tamoxifen-resistant cells, was demonstrated by evaluation of ER-mediated transactivation, as it is well known that the ER transcriptional activity is reprogrammed upon development of endocrine resistance^16,17^. TFF1 (pS2), an estrogen-regulated gene, was consistently downregulated in tamoxifen-resistant and parental cells following MCM3 knockdown as determined by RT-qPCR (Fig. 5e), while ER levels in tamoxifen-resistant and parental cells were not significantly altered (Fig. 5f).

### MCM3 Reduction Alters Phosphorylation of Cell Cycle Regulator Proteins in Tamoxifen-Resistant Cells

To understand how MCM3 expression may influence tamoxifen resistance, the TamR-1 cells following MCM3 knockdown were analyzed for altered expression of downstream cell cycle-associated molecules using protein/phosphoprotein arrays. Analysis of the antibody-based microarray showed that phospho-protein vs. non-phospho-protein ratios of Cdc25B(Ser^323^)/Cdc25B, Chk1(Ser^280^)/Chk1, Chk1(Ser^317^)/Chk1, p21^Cip1^(Thr^145^/p21^Cip1^, p53(Ser^315^)/p53 and p53(Ser^33^)/p53 were consistently increased when MCM3 expression was reduced (Fig. 5g). IPA analysis of the proteins exhibiting altered phosphorylation revealed cell cycle G1/S checkpoint regulation and G2/M DNA damage checkpoint regulation as the top canonical pathways, and identified one protein network (Supplementary Fig. 7). Phosphorylation of all sites shown in Fig. 5g has been reported to cause activation of cell signaling, and most are associated with DNA damage response and cell cycle arrest, e.g., p53 is phosphorylated at multiple sites in response to DNA damage, including Ser33 and Ser315, which we found to be increased, leading to p53-dependent cell cycle arrest. Phosphorylation of Chk1 at both Ser317 and Ser280, also found to be increased, are both central for the activation of replication checkpoints. Downstream of Chk1, DNA damage causes phosphorylation of Cdc25B at Ser323, which were also increased, mediating G2/M cell cycle arrest. Finally, phosphorylation of Myc at Thr358 and Ser373, also increased, regulates Myc transcriptional activity and Myc phosphorylation at these sites, decreasing the affinity of Myc for Myc-associated factor X and thus decreasing binding of E-box DNA elements. Considering that the phosphorylated forms of several of the above-mentioned cell cycle regulators act as inhibitors of cell cycle progression, increased MCM3 expression in tamoxifen-resistant cells may suppress activation of cell cycle inhibitors and thereby overcome the anti-proliferative effects of tamoxifen.

### Efficacy of CDK4/6 inhibitor treatment is independent of MCM3 expression

**Fig. 6:**
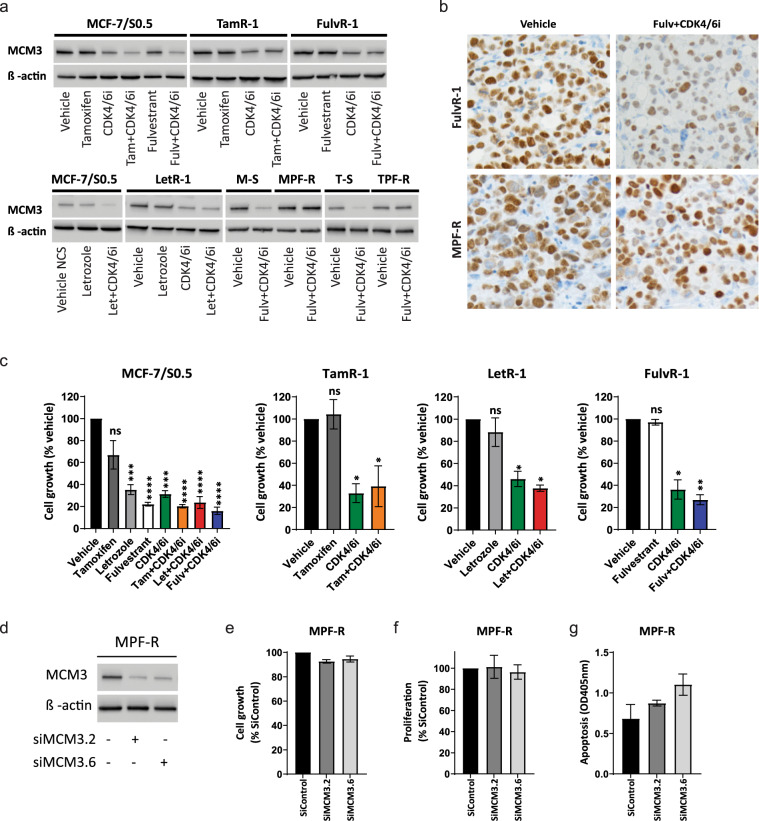
Efficacy of CDK4/6 inhibitor is independent of MCM3 expression. (**a**) MCM3 protein expression was evaluated following treatment with CDK4/6 inhibitor (CDK4/6i) in endocrine-resistant cells, including tamoxifen- (TamR-1), aromatase inhibitor- (LetR-1) and fulvestrant-resistant (FulvR-1), and in cell lines resistant to combined CDK4/6i and endocrine therapy (MPF-R and TPF-R), by Western blotting using whole cell lysate. β-actin was used as loading control. A representative of two biological replicates is shown. (**b**) Representative micrographs (40x magnification) showing MCM3 immunhistochemistry staining of FulvR-1 and MPF-R tumor xenografts treated with CDK4/6i (50 mg/Kg) combined with fulvestrant (100 mg/Kg; *n* = 7 and 9) or vehicle (castor oil and 25% w/v HPB cyclodextrin; *n* = 8 and 10). (**c**) Evaluation of growth of endocrine-resistant cells following treatment with CDK4/6i as measured by colorimetric crystal violet assay at 96 h. Data are representative of three independent experiments and are shown as ± s.e.m in triplicates. (**d**) Transfection of MPF-R cells with 2 separate MCM3 targeting siRNAs (siMCM3.2 and siMCM3.6) resulted in a marked reduction of MCM3 protein levels as evaluated by Western blotting 96 h post siRNA transfection. β-actin was used as loading control. A representative of two biological replicates is shown. Knockdown of MCM3 expression caused no significant changes in growth, proliferation, or apoptosis as measured by colorimetric crystal violet (**e**), BrdU incorporation (**f**), and cell death (**g**) assays, respectively, at 96 h after transfection. Data were confirmed in 3 independent experiments and are shown as ± s.e.m in triplicates. *p < 0.05.

CDK4/6 inhibitors (CDK4/6i) have shown impressive improvement of PFS and OS in ER^+^ advanced breast cancer in combination with either aromatase inhibitors or fulvestrant, and these combinations have recently become standard-of-care in this patient population^18–23^. Moreover, combined CDK4/6i and endocrine therapy is currently being tested in the adjuvant setting (NCT02513394, NCT03155997 and NCT03701334) and is expected to also be approved for treatment of primary ER^+^ breast cancer. Therefore, we investigated the role of MCM3 in the response to CDK4/6i. First, we evaluated MCM3 expression by Western blotting after treating ER^+^ breast cancer cells with CDK4/6i alone and in combination with endocrine therapy. We found that CDK4/6i alone reduced MCM3 levels in both endocrine-sensitive (MCF7/S0.5) and -resistant (TamR-1, LetR-1, and FulvR-1) breast cancer cell lines (Fig. 6a). This reduction in MCM3 expression was more significant when CDK4/6i was combined with endocrine therapy in endocrine-sensitive cells, but not in endocrine-resistant cell lines (Fig. 6a). Importantly, no effect on MCM3 expression was observed in ER^+^ breast cancer cell lines resistant to combined CDK4/6i and fulvestrant (MPF-R and TPF-R cells) after treatment with combined therapy (Fig. 6a). We also evaluated the effect of CDK4/6i on MCM3 expression in vivo by transplanting mice with FulvR-1 and MPF-R cells. When tumors reached 100–150 mm^3^, mice were treated with combined CDK4/6i and fulvestrant or vehicle. After sacrifice, the tumors were removed and stained for MCM3 protein expression by immunohistochemistry. Comparable to the results in vitro, we observed that treatment with combined CDK4/6i and fulvestrant decreased MCM3 levels in endocrine-resistant FulvR-1 tumors, but not in MPF-R tumors, which are resistant to combined CDK4/6i and endocrine therapy (Fig. 6b). Next, we investigated whether MCM3 expression affects response to combined CDK4/6i and endocrine therapy in ER^+^ breast cancer cell lines. We observed that growth of both MCM3-low (MCF-7/S0.5 and FulvR-1) and -high (TamR-1 and LetR-1) cell lines was significantly inhibited by CDK4/6i alone or combined with endocrine therapy (Fig. 6c). Furthermore, we found no significant difference in CDK4/6i IC50 between tamoxifen-resistant (high-MCM3) and -sensitive (low-MCM3) cells in either MCF-7 or T47D models (Supplementary Fig. 8a, b). However, letrozole-resistant (high-MCM3) cells showed higher CDK4/6i IC50 compared to the parental cell line MCF-7 (low-MCM3) (Supplementary Fig. 8a). Importantly, CDK4/6i IC50 was not significantly altered following MCM3 knockdown of endocrine-sensitive and -resistant cells (Supplementary Fig.8c and 8d). Together, these results suggest that the effect of CDK4/6i on endocrine-sensitive and -resistant cells is independent of MCM3 levels. To further investigate whether MCM3 is involved in resistance to combined CDK4/6i and endocrine therapy, we performed transient knockdown of MCM3 in MPF-R cells using two different siRNAs, siMCM3.2 and siMCM3.6, which significantly reduced MCM3 protein levels, as evaluated by Western blotting (Fig. 6d). We found that MCM3 knockdown did not significantly alter MPF-R cell growth, proliferation or apoptosis (Fig. 6e–g). Together, these results suggest that MCM3 does not confer resistance to CDK4/6i. Finally, we examined whether MCM3 is a marker of resistance to CDK4/6i, and compared it with another cell cycle molecule, AURKA, which is known to be involved in the mechanisms of resistance to CDK4/6i^24^. We evaluated the expression of MCM3 and AURKA by immunohistochemistry in metastatic lesions from ER^+^ advanced breast cancer patients treated with combined CDK4/6i and endocrine therapy (*n* = 86). Neither MCM3 or AURKA showed a significant correlation with PFS in this patient cohort (*p* = 0.31 and *p* = 0.81, respectively; Supplementary Fig. 9a, b). Furthermore, subgroup analysis according to endocrine status (sensitive, intrinsic, or acquired resistance) did not identify a correlation between MCM3 level and PFS (Supplementary Fig. 9c). There were no patients with intrinsic resistance to endocrine therapy whose tumors expressed low MCM3 (Supplementary Fig. 9c). However, ER + advanced breast cancer patients with intrinsic resistance to endocrine therapy and treated with combined CDK4/6i and endocrine therapy whose tumors expressed low AURKA exhibited significantly shorter PFS (Supplementary Fig. 9d). The clinical findings that MCM3 does not correlate with response to CDK4/6i are in line with our in vitro data showing that MCM3 is not involved in resistance to CDK4/6i.

## Discussion

To gain insight into the biology of endocrine resistance in breast cancer, and to identify potential predictive or prognostic markers, we used a quantitative proteomic approach combined with systems biology analyses. Cell cycle and DNA replication were particularly enriched in the differentially expressed proteins, including MCM3 and other MCM proteins as well as ATR kinases that showed increased expression in tamoxifen-resistant vs. -sensitive cell lines and were found to be central in the highest-ranked subnetwork. MCM3 proteins, together with MCM2-7, form a heterohexamer complex, which is the final component loaded onto origins of DNA replication sites^25,26^. We propose that the increased expression of MCM proteins and ATR kinases in tamoxifen-resistant cells allow them to escape G0/G1 cell cycle arrest as induced in tamoxifen-sensitive cells upon tamoxifen treatment. This is supported by our findings that lowering MCM3 protein expression levels in multiple tamoxifen-resistant and letrozole-resistant cell lines restored their sensitivity to tamoxifen or letrozole. We expect this will also be the case for the other MCM proteins as they exhibit highly similar function, and also exhibited correlation with outcome in cohort 3 (Supplementary Fig. 7). However, we chose to focus on MCM3 as it was central in a functional subnetwork of regulated proteins with DNA replication and cell cycle functions. Earlier reports have shown that in vitro estrogen treatment of ER^+^ breast cancer cell lines affected MCM3 expression^27^, and that a number of different tumor types including breast cancer exhibit increased expression compared to surrounding normal tissues^28^. These data suggest that MCM3 may play a potential role in tumor development and growth.

The clinical relevance of MCM3 levels was demonstrated by its strong correlation with RFS and OS in 4 independent cohorts consisting of 79, 218, 2305, and 683 early-stage, ER^+^ breast cancer patients receiving adjuvant tamoxifen or no adjuvant treatment. Importantly, the results indicated that MCM3 was not only a prognostic marker in adjuvant tamoxifen-treated early-stage breast cancer patients, but also a predictive marker of tamoxifen benefit, as we found a correlation between MCM3 levels and outcome in the tamoxifen adjuvant treatment arm, but not in the no-adjuvant-treatment arm of ER^+^ breast cancer patients. Further, no correlation between MCM3 levels and outcome was observed in early-stage ER- breast cancer patients.

Among biomarkers of clinical relevance in breast cancer, the proliferation marker Ki67 has shown promise as a prognostic marker. However, the use of Ki67 in the clinical routine varies due to limitations such as lack of standardized assessment and interpretation^29^. We compared the prognostic potential of Ki67 with MCM3 in 4 different cohorts of ER^+^ breast cancer patients treated with anti-hormonal therapy. In the first 3 cohorts, both MCM3 and Ki67 showed significant prognostic potential. However, in cohort 4, MCM3, but not Ki67, was significantly correlated to clinical outcome in the tamoxifen-treated population despite using the same cut-off for Ki67 in all cohorts (>15%). Interestingly, comparison of the four cohorts in terms of clinical outcome showed that cohort 4 consisted of patients who were lymph node-negative, generally had smaller tumors and lower recurrence rates than the first 3 cohorts. It has been widely shown that Ki67 is a good prognostic marker in high-risk patients, but performs poorly in the low-risk patients subgroup (reviewed in^30^). This, and the lack of standardized cut-off for Ki67 measurement, have limited its use in clinical decision-making. Our results indicate that MCM3 may be a more robust prognostic marker, easy to assess by immunohistochemistry, and works in both low- and high-risk ER^+^ breast cancer patients.

Our study showed that reduction of MCM3 increased the activating phosphorylation of Chk1(Ser^317^), which is a phosphorylation site of ATR^31^. This suggests a possible involvement of the Chk1/ATR canonical cell cycle signaling cascade normally associated with genotoxic stress-related single-stranded DNA^32^, and double-stranded DNA damage^33^. Although several cell cycle-related molecules, such as Rb, Cyclin E, Cyclin D, CDK6, p16, and AURKA, have been associated with resistance to the recently approved CDK4/6 inhibitor^24,34–38^, our study showed that reduction of MCM3 level does not impair the efficacy of this inhibitor.

In conclusion, our results showed increased expression of MCM3 in endocrine-resistant breast cancer cell lines, and that this upregulation plays a significant role in protecting cells against tamoxifen- or letrozole-mediated growth inhibition and apoptosis. Moreover, high expression of MCM3 in primary breast tumors is not only a strong and consistent prognostic marker, but also predictive of diminished response to tamoxifen therapy.

## Methods

### Cell lines and Standard Culture Conditions

The human breast cancer cell line MCF-7 was originally received from The Breast Cancer Task Force Cell Culture Bank, Mason Research Institute (Worcester, MA). The MCF-7 cells were gradually adapted to grow in low serum concentration and the tamoxifen-sensitive subline MCF-7/S0.5^39^ was used to establish the tamoxifen-resistant cell lines MCF-7/TAM^R^-1 (TamR-1), MCF-7/TAM^R^-4 (TamR-4), and MCF-7/TAM^R^-7 (TamR-7) by extended treatment with high dose tamoxifen (10^−6^M) for 18–20 passages^6,40,41^. Although generated in parallel, the three TamR cell lines exhibited considerable differences. The MCF-7/0.5 cell lines were grown in standard phenol-red-free DMEM:F12 (1:1) medium (Gibco, 21041-025) supplied with 1% heat-inactivated FBS (Gibco, 10270-106), 6 ng/ml insulin (Sigma-Aldrich, I6634) and 2.5 mM glutamax (Gibco, 35050). The standard medium for TamR-1, TamR-4, and TamR-7 was the same as the medium of parental cells supplied with 10^−6^ M tamoxifen (Sigma-Aldrich, T5648). Fulvestrant-resistant cell line FulvR-1 was developed from MCF-7/S0.5 cells, by extended treatment with high dose fulvestrant 10^−7^M (Tocris, ICI 182,780) for 4–5 months^42^, and maintained in the same growth medium as MCF-7/S0.5 cells supplemented with 100 nM fulvestrant. MCF-7-derived cell lines resistant to combined CDK4/6 inhibitor (CDK4/6i, MedchemExpress, HY-A0065) and fulvestrant (MPF-R) were developed from FulvR-1 cells by prolonged treatment (4 months) with 150–200 nM of CDK4/6i and 100 nM of fulvestrant and maintained in the same growth medium as FulvR-1 cells supplemented with 200 nM CDK4/6i. MCF-7-sensitive cells grown in parallel with MPF-R cells were designated M-S. The tamoxifen-sensitive T47D/S2 cell line was gradually adapted to grow in medium with 2% FBS. The tamoxifen-resistant cell lines T47D/TR-1 and T47D/TR-2 were established from T47D/S2 by long-term treatment with tamoxifen (10^−6^ M) for 10 months^43^. The T47D-derived cell line model was cultured in phenol-red free RPMI1640 medium supplied with 2% heat-inactivated FBS, 8 µg/ml insulin, and 2.5 mM glutamax. The standard medium for T47D/TR-1 and T47D/TR-2 was the same as the medium of parental cells supplied with 10^−6^ M tamoxifen. T47D cells resistant to fulvestrant and CDK4/6i (TPF-R) were established from T47D-derived fulvestrant-resistant cells^44^ by long-term treatment (3 months) with 100 nM fulvestrant and 150–200 nM CDK4/6i and maintained in the same growth medium as T47D/S2 cells supplemented with 5% heat-inactivated FBS, 100 nM fulvestrant and 200 nM CDK4/6i. T47D-sensitive cells grown in parallel with TPF-R were designated T-S. From the MCF-7/S0.5 cell line, the letrozol-resistant cell line MCF-7/S0.5/Let^R^-1 (LetR-1) was established by selection of surviving colonies from long-term AI treatment (10^−6^ M letrozole, Selleck Chemicals) of the MCF-7/S0.5 cells grown under conditions where endogenous aromatase-mediated conversion of androgen to estrogen was required for growth (DMEM/F12 medium supplemented with 10 % newborn calf serum (NCS, Life Technologies), 10^−7^ M testosterone, 6 ng/mL insulin, and 2.0 mM glutamax. The AI-resistant cell line was maintained in DMEM/F12 medium with 10 % NCS, 10^−7^ M testosterone, 6 ng/mL insulin, 2.0 mM glutamax, and 10^−6^ M letrozole. Previous studies have shown that the AI-resistant cell line had acquired the ability to proliferate without aromatase-mediated conversion of testosterone to estradiol. However, when AI treatment was withdrawn, testosterone caused minor growth stimulation^45^. Letrozole was able to inhibit the stimulation by testosterone, but was unable to reduce growth to below the level in standard growth medium with AI. In contrast, fulvestrant totally abolished growth of the AI-resistant cell line both after AI was withdrawn and in the presence of AI treatment. Together this indicates that ER is the main driver of growth of the AI-resistant cell line and points to ligand-independent activation of ER^45^. The cell lines were cultured at 37 °C and 5% CO_2_ and kept at maximum 10 passages throughout the experiments to minimize possible phenotypic changes. The cell lines were verified as being free of mycoplasma contamination.

### Mass spectrometry-based proteomic analysis

MCF-7/S0.5 and TamR-1 cells were SILAC-labeled (Stable Isotope Labeling with Amino Acids) and prepared for mass spectrometry analysis as described in the Supplementary Methods (available online). MS analysis was performed on an LTQ-Orbitrap-Velos (Thermo Fisher Scientific) connected to an Agilent 1100 nanoflow HPLC system (Agilent) using a nanoelectrospray ion source (Proxeon Biosystems). All raw files were processed with MaxQuant v. 1.0.13.13 into centroided data and submitted to the database with Mascot v.2.2 (Matrix‐Science). Data from MaxQuant processing was subjected to analysis using the statistical environment R (RDevelopmentCoreTeam). More details regarding processing and bioinformatic analysis of proteomic data, processing of gene microarray datasets, Western blot analysis, quantitative real-time PCR, phospho-specific cell cycle antibody microarray analysis are provided in the Supplementary Methods (available online).

### Characteristics of Patient Cohorts

Formalin-fixed, paraffin-embedded (FFPE), ER^+^ primary breast cancer samples from three comparable, but independent, patient populations were analyzed (Supplementary Table 1). In addition, microarray data from 2555 breast cancer patients obtained from kmplot.com database were analyzed. The first cohort consisted of ER^+^ primary breast cancer tissues from 79 patients collected from Herlev and Roskilde Hospitals, Denmark. Of these 68 had sufficient tumor for staining. All tumors were ERα + (> 10%), 54% had tumor sizes > 20 mm, and 92% of the patients were lymph node-positive. These patients were part of a nationwide study of 1,115 Danish postmenopausal early-stage ER^+^ breast cancer patients who received 20 mg adjuvant tamoxifen daily for 5 years following radical surgery between 1995 and 2006. Eight-ten patients were excluded due to lack of tissue, leaving a total of 68 evaluable samples^46,47^. The second cohort was extracted from a retrospective cohort of 589 patients from the Danish Breast Cancer Co-operative Group (DBCG) 89C randomized study^46,48^. The selected patients consisted of 218 post- or peri-menopausal patients who, between 1989 and 2001, had lumpectomy or mastectomy at Odense Hospital. These patients received adjuvant tamoxifen mono-therapy for 0.5, 1, 2, or 5 years. Inclusion criteria included one of the following: positive axillary lymph nodes, tumor size > 50 mm (> 20 mm since 1999) and/or ductal grade II–III (since 1999), and therefore defined as high-risk patients. An additional criterion for inclusion was age at surgery less than 75 years. Patients were selected on the basis of the availability of concurrent fresh-frozen tumor tissue and positive hormone receptor status (ERα or PGR). None of the cancer patients included in the cohorts had received adjuvant cytotoxic therapy, treatment with AIs, or neo-adjuvant endocrine treatment. The third cohort included 2555 breast cancer patients of which 2051 were ER^+^ (1802 were endocrine treated and 503 did not receive any systemic treatment). The remaining 250 were ER- patients. All data for cohort 3 were obtained from www.kmplot.com database, a net-based survival analysis tool where microarray data from over 4000 breast cancer patients was curetted and made publicly available (kmplot.com)^14^. The fourth cohort originated from the Stockholm Breast Cancer Study Group randomized tamoxifen STO-3 trial 1976–1990^15^. A cohort of 1,780 postmenopausal women with breast cancer were randomized to adjuvant tamoxifen for 2 or 5 years (*n* = 886), or no adjuvant endocrine therapy (*n* = 894). The patients were all postmenopausal at the time of diagnosis and had a tumor size ≤ 30 mm (76% ≤ 20 mm) and lymph node-negative (N0), thus defined as low-risk patients. The patients had been treated with modified radical mastectomy (*n* = 1348) or breast-conserving surgery plus radiation therapy (*n* = 432). Since the treatment predictive value of hormone receptor status in the adjuvant setting was not certain at the time of the trial, no selection on the basis of hormone receptor status was made. TMA was originally generated from primary tumors of 910 of these patients, and sufficient tumor tissue of 683 patients remained available for MCM3 assessment. The clinicopathological characteristics in this subset were similar to those in the complete series of 1,780 patients, such as a tumor size ≤ 20 mm (76% vs. 78%), ER-positive status (78% vs. 80%), and tamoxifen treatment (51% vs. 50%). The tumors were graded retrospectively according to the Nottingham system (NHG) by one pathologist blinded to clinical outcome. The study was approved by the local ethical committee at Karolinska Institute, Stockholm, Sweden. The biomarker study was conducted according to the REMARK recommendations^49^. FFPE metastatic lesions from ER^+^ breast cancer patients treated with combined CDK4/6i and endocrine therapy in the advanced setting were selected retrospectively by database extraction from the archives of the Department of Pathology at Odense University Hospital (*n* = 115). ER^+^ breast cancer patients treated with combined CDK4/6i and endocrine therapy in the advanced setting who had undergone surgery or biopsy for advanced-stage disease at Odense University Hospital were included. Samples were excluded if there was insufficient tumor material in the FFPE block or if the metastatic biopsy was only available after treatment with combined CDK4/6i and endocrine therapy. These parameters yielded *n* = 86 patients. All clinical samples were coded to maintain patient confidentiality and the studies were approved by the Ethics Committee of the Region of Southern Denmark and Copenhagen and Frederiksberg Counties (approval no. S-20080115, S-20170154, and 01025-KF12-138-99) and the Danish Data Protection Agency (2008-58-0035). All tissue samples were collected in compliance with informed consent policy.

### Clinical Data: Endpoints

For cohorts 1 and 2, clinical data on post-surgical patients were retrieved from the DBCG registry and by record linkage to the Danish Central Population Registry (date of death). For the first cohort, recurrence-free (RFS) and overall survival (OS) were defined as the time from surgery to recurrence or death within 10 years, respectively. Patients without recurrence/death were censored at the date of emigration, 10 years after the date of surgery, or to the date of clinical database retrieval from the national registry (July 12011), whichever came first. For the second cohort, RFS was defined as the time from surgery to the date of recurrence within 10 years. Exclusion criteria were: recurrence < 3 months of surgery, any treatment with AIs, bilateral breast cancer, and secondary primary cancers. OS was defined as the time from surgery to death within 10 years regardless of cause or end of follow-up. Patients who were recurrence-free or alive at 10-years follow-up were censored at the date of emigration, 10 years after surgery, or on the date of clinical database retrieval from DBCG (June 6, 2012), whichever came first. With regard to OS, no patients were lost to follow-up due to database linkage to the Danish Civil Registration System. For the third cohort, follow-up data was obtained from a public database in kmplotter.com that was collected and curated for use in survival analysis. For the 4th cohort, follow-up data were collected from regional population registers and the Swedish Cause of Death Registry and the median follow-up period for the patients was 17 years. RFS and breast cancer-specific survival (BCSS) were chosen as primary endpoints. For the cohort of ER^+^ advanced breast cancer patients treated with CDK4/6i, progression-free survival (PFS) was chosen as the endpoint and defined as the time from initiation of combined endocrine therapy and CDK4/6i treatment until disease progression or death.

### Xenograft tumor models

FulvR-1 and MPF-R cells (1 × 10^6^) were resuspended in 50 ul of extracellular matrix (ECM) from Engelbreth-Holm-Swarm sarcoma (Sigma-Aldrich) and injected orthotopically into the mammary fat pad of 7-week-old female NOG CIEA mice (Taconic) without exogenous estrogen supplements. When the FulvR-1 and MPF-R tumor xenografts reached 100–150 mm^3^, treatment with CDK4/6i (palbociclib, 50 mg/Kg bodyweight) combined with fulvestrant (100 mg/Kg bodyweight; *n* = 7 and 9) or vehicle (castor oil and 25% w/v HPB cyclodextrin; *n* = 8 and 10) was initiated and continued for up to 7 weeks. CDK4/6i was administered by oral gavage once daily for 5 days a week, whereas fulvestrant was administered subcutaneously once a week. At the end of treatment, animals were euthanized, tumors excised, and FFPE. The animal experiment was approved by the Experimental Animal Committee of The Danish Ministry of Justice and was performed at the animal core facility at University of Southern Denmark.

### Immunohistochemical Staining

Tissue microarrays (TMAs) of cohorts 1 and 2 contained two tissue cores per patient tumor with a diameter of 2 mm, while the TMA of cohort 4 contained three tissue cores per patient tumor with a diameter of 0.5 mm. TMAs and whole FFPE sections (4 μm) of metastatic lesions from patients treated with combined endocrine therapy and CDK4/6i and tumor xenografts were cut with a microtome, mounted on ChemMateTM Capillary Gap Slides (Dako), dried at 60 °C, deparaffinized, and hydrated. Endogenous peroxidase was blocked by 1.5% hydrogen peroxide in TBS buffer, pH 7.4, for 10 min. Antigen retrieval was performed by pretreatment with cell conditioner 1 (CC1) buffer for 32 minutes at 100 °C or 36 minutes at 36 °C, or by boiling sections in T-EG solution/TRS buffer (Dako)^50^. Whole biopsy or TMAs FFPE sections were incubated with anti-MCM3 (1:750 and 1:500, HPA004789, Atlas Antibodies), anti-Ki67 (Cohorts 1 and 2: ready to use, clone 30-7, Ventana)^51^, anti-Ki67 (Cohort 4: MIB-1; DAKO M7240)^52^ or anti-AURKA (1:1000, HPA002636, Sigma-Aldrich) using the PowerVision + ™ Poly-HRP (Leica, PV6104), Ultraview DAB detection kit (Ventana), EnVision Plus (Dako) and EnVision FLEX/HRP (Dako), respectively, on the autostainer (TechMate™ 500, Dako) or Ventana system. Evaluation of the immunohistochemistry staining was performed by a skilled breast pathologist in a blinded setup. MCM3 staining was located to the nucleus, and tumors were considered MCM3-positive (MCM3^+^) if 10% of the cells were clearly stained. Normal cells, such as lymphocytes and stromal cells, were generally negative for MCM3, making the scoring straightforward. For the majority of the TMAs, 2–3 cores were scored separately and good concordance was observed between them. In case of a discrepancy between the score of 2–3 paired cores, positive staining in just one core was deemed sufficient to be recorded as positive staining. Tumors were considered Ki67-positive (Ki67^+^), if > 15% of the tumor cells were stained for Ki67. AURKA staining was located in the nucleoplasm, centrosome and cytosol, and tumors were considered AURKA-positive (AURKA^+^) if 10% of the cells were clearly stained.

### Targeted Gene Knockdown using siRNA

Cells grown under standard culture conditions were harvested at 80% confluence. Cells (2 × 10^6^) were transfected with a combination of two or a single small interfering RNAs (siRNAs) (300 nM) in 100 µl nucleofector solution v (Cell Line Nucleofector^®^ Kit V, Amaxa) using electroporation P20 (nucleofector^®^ II, Amaxa) at room temperature according to manufacturer’s protocol. MCM3-specific siRNAs were obtained from Qiagen: SI02664879, SI00037009, while Control siRNA (siControl) was obtained from Sigma-Aldrich; MISSION^®^ siRNA Universal Negative Control: sic001. Transfected cells were cultured in standard culture medium. Gene knockdown efficiency was evaluated at 48 h using RT-qPCR. Cells were seeded in triplicates to measure cell growth after 48 and 120 h using the colorimetric crystal violet staining (absorbance at 590 nm) and for immunoblot assessment of protein expression levels 48, 72, 96, and 120 h after transfection. For the BrdU proliferation assay, cells were labeled with 10 µM BrdU for 16 h, stained with BrdU antibody and HRP-conjugated secondary antibody according to the manufacturer’s recommendations (BrdU cell proliferation assay kit; Cell Signaling). The signal was developed with TMB substrate and read at 450 nm. Total RNA was purified from TamR and MCF-7/S0.5 cells transfected with specific or siControl for 48 h using RNA kit (Qiagen) and arrayed separately. The effect of MCM3 knockdown on apoptotic cell death was assessed using cell death detection ELISA^plus^ assay (Roche) according to the manufacturer’s instructions. Briefly, cells transfected with siMCM3.2, siMCM3.6, or siControl were seeded in 24-well plates at a density of 4 × 10^4^ cells per well. After 96 h, the supernatant was removed, adherent cells were lysed in 200 μl lysis buffer and the lysates were assayed for levels of nucleosomes using the immunoreagent from the kit.

### Statistics

A two-tailed t-test was used to compare proliferative responses in cells following targeted gene knockdown. The association of protein expression to clinicopathological parameters was determined by Chi-square or Fisher’s exact test. The log-rank test was applied to examine the association between selected protein expression and clinical end-points, including RFS, OS and PFS. Results were depicted by Kaplan-Meier curves. The Cox proportional hazard regression model was used to calculate the hazard ratio (HR) of the protein expressions and standard clinical prognostic parameters using both the univariate and multivariate models. All statistical calculations were done with the statistical software GraphPad Prism or STATISTICA 10 (StatSoft Inc.). For microarray and proteomic data analysis false discovery rate of 0.05 was set as a cut-off for statistical significance, while *p*-values less than or equal to 0.05 in two-sided tests were considered significant for all other statistical tests involving 2-group comparisons. MCM3 expression in tumors was dichotomized as MCM3^+^ or MCM3^−^ using a cut-off of 10%, which was set and tested in cohort 1 and used in all subsequent cohorts.

### Reporting Summary

Further information on research design is available in the Nature Research Reporting Summary linked to this article.

## Supplementary information

Supplemental Materials

Reporting Summary Checklist

## Data Availability

The mass spectrometry proteomics data generated during the study, are publicly available in the PRIDE repository, under the accession number https://identifiers.org/pride.project:PXD001087^53^. The effect of MCM3 knockdown on gene expression in TamR cell lines data, are publicly available in Gene Expression Omnibus, under the accession number: https://identifiers.org/geo:GSE148878^54^. The three microarray gene datasets analyzed during the study, are publicly available in Gene Expression Omnibus, under the following accession numbers: https://identifiers.org/geo:GSE20361^55^, https://identifiers.org/geo:GSE38829^56^, and https://identifiers.org/geo:GSE50820^57^. Microarray data from 2555 breast cancer patients (cohort 3), analyzed during the study, were obtained from the kmplot.com database (www.kmplot.com). Survival analyses and immunohistochemistry data, are not publicly available to protect patient privacy, but will be made available to authorized researchers who have an approved Institutional Review Board application and have obtained approval from The Regional Committees on Health Research Ethics for Southern Denmark. Please contact the corresponding author with data access requests. All other datasets generated during the study will be made available upon reasonable request from the corresponding author, Dr. Henrik Ditzel, email address: hditzel@health.sdu.dk. Supplementary Tables 1 and 4 are available in the figshare repository: 10.6084/m9.figshare.13234520^58^. Uncropped Western blots are part of the supplementary files.
