# Supplementary material for: MCM3 upregulation confers endocrine resistance in breast cancer and is a predictive marker of diminished tamoxifen benefit
Source: NPJ Breast Cancer. 2021 Jan 4;7:2. doi: 10.1038/s41523-020-00210-8 (PMC7782683; doi:10.1038/s41523-020-00210-8)
Supplement: Supplementary file 1 — Supplemental Materials [file 41523_2020_210_MOESM1_ESM.pdf]

Supplementary Materials

MCM3 upregulation confers endocrine resistance in breast cancer and is a predictive marker of diminished tamoxifen benefit.

S. Løkkegaard, D. Elias, C. L. Alves, M. V. Bennetzen, A.-V. Lænkholm, M. Bak, M. F. Gjerstorff, L. E. Johansen, H. Vever, C. Bjerre, T. Kirkegaard, B. Nordenskjöld, T. Fornander, O. Stål, L. S. Lindström, L. J. Esserman, A. E. Lykkesfeldt, J. S. Andersen, R. Leth-Larsen and H. J. Ditzel.

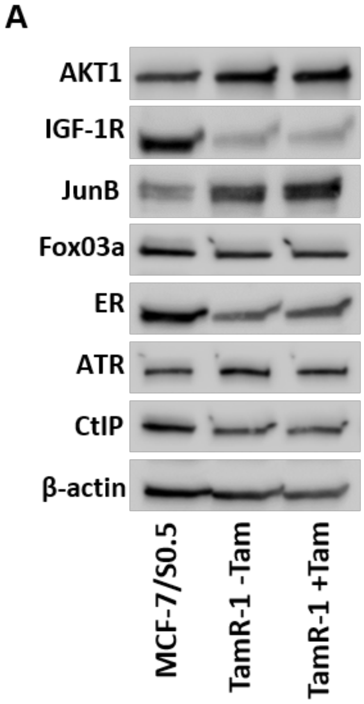

Supplementary Figure 1. Validation by Western blotting of the altered expression of selected proteins identified by quantitative proteomic analysis. (**A**) Altered expression of selected proteins in tamoxifen-resistant TamR-1 cells grown in the presence of tamoxifen (TamR-1 +Tam) or absence of tamoxifen (TamR-1 -Tam) vs. parental MCF-7/S0.5 cells was validated by Western blotting using whole cell lysates. β-actin was used as loading control. A representative of two biological replicates is shown.

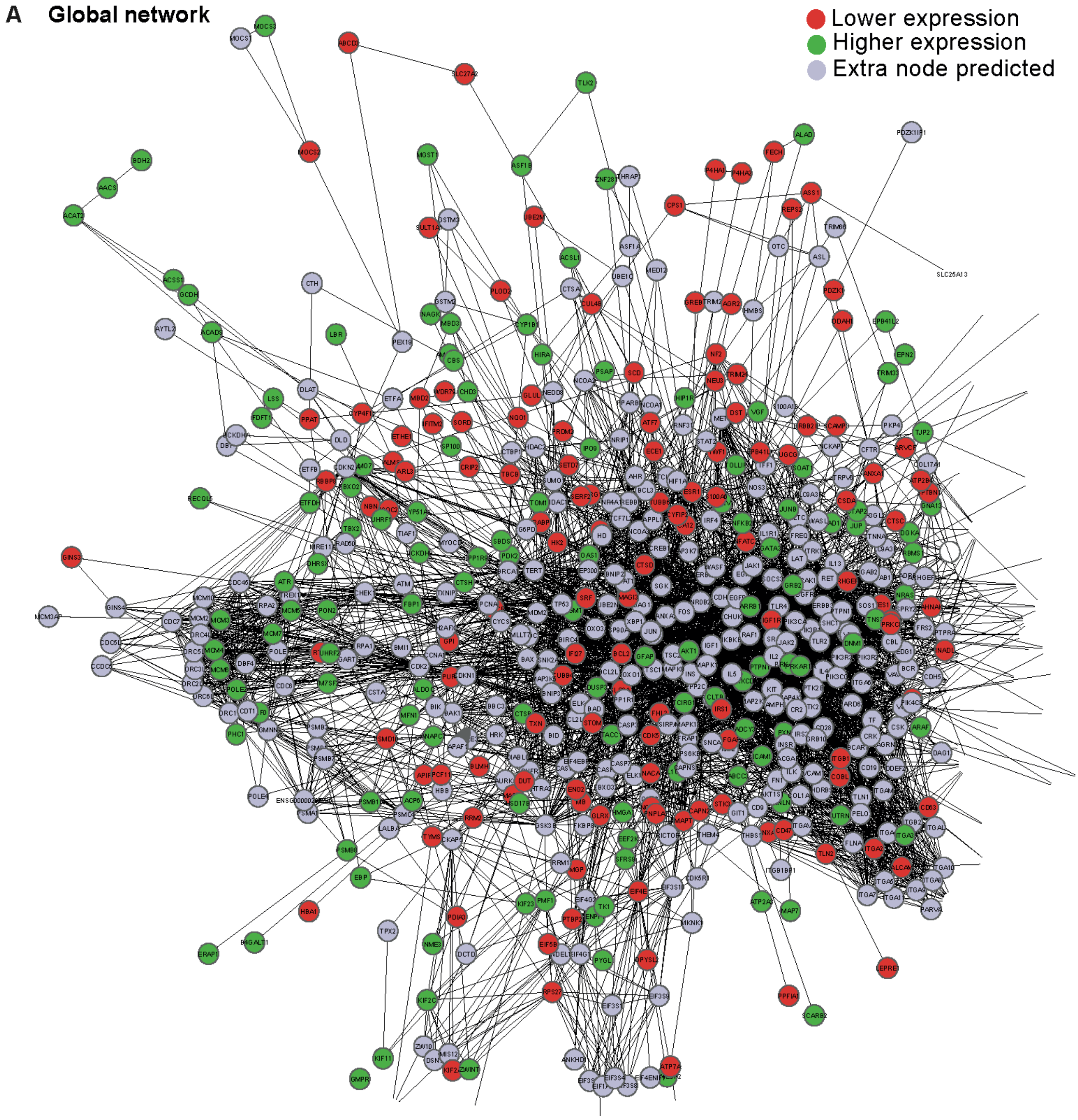
A Global network
Lower expression
Higher expression
Extra node predicted

B

Subnetwork 2

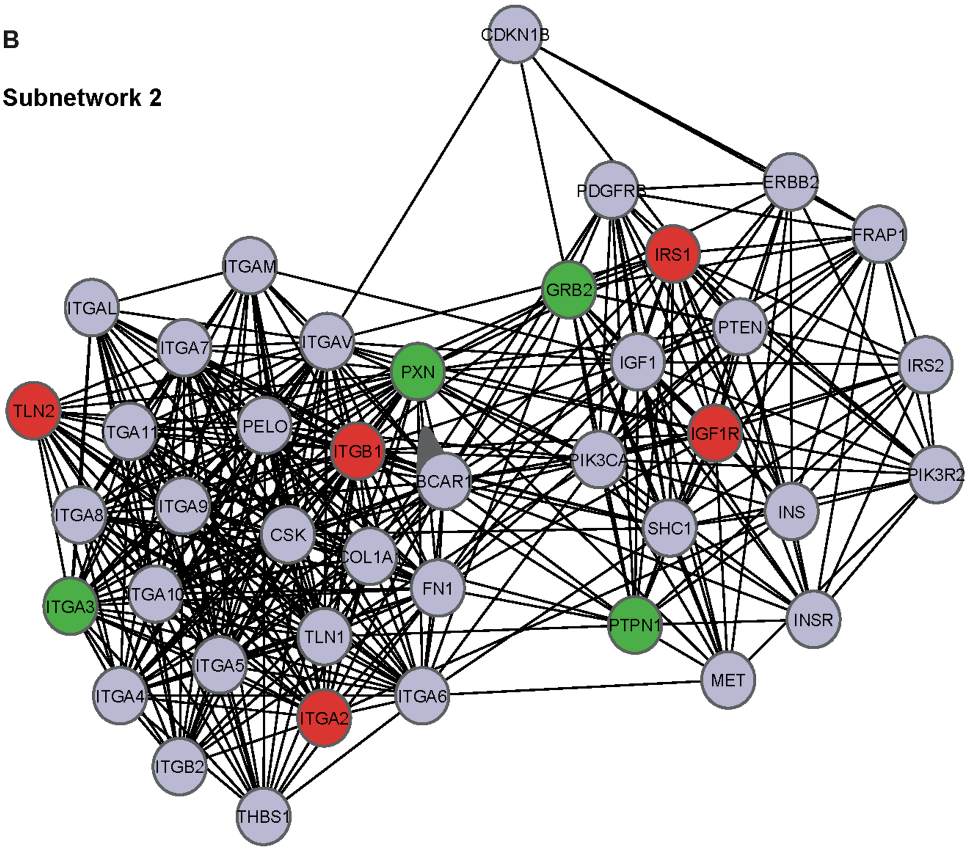

Subnetwork 3

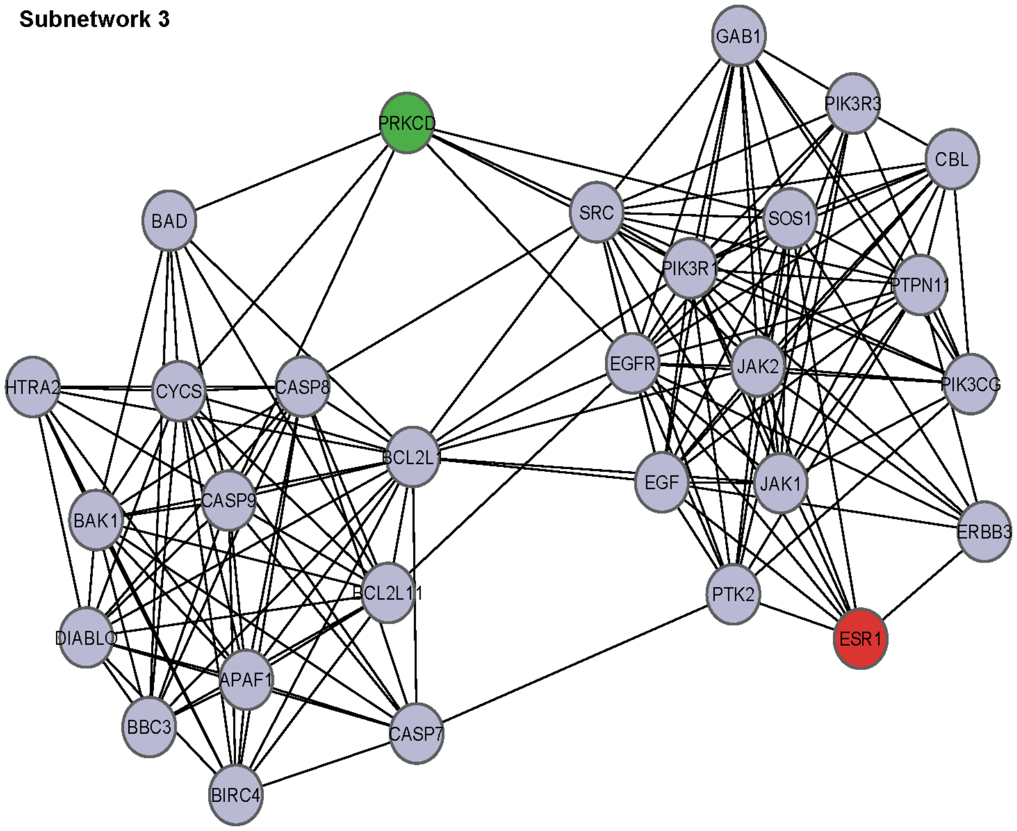

Subnetwork 4

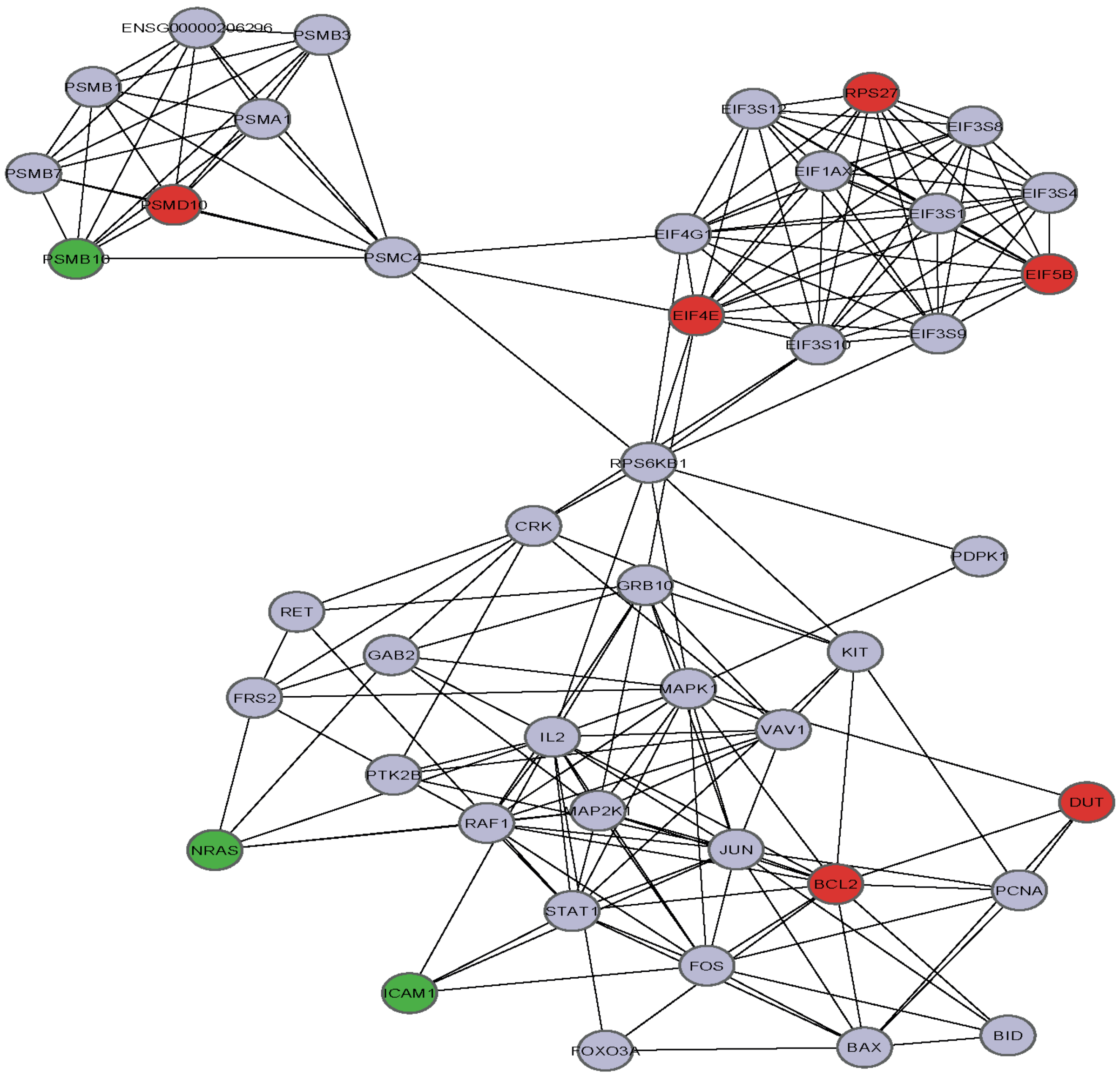

Subnetwork 5

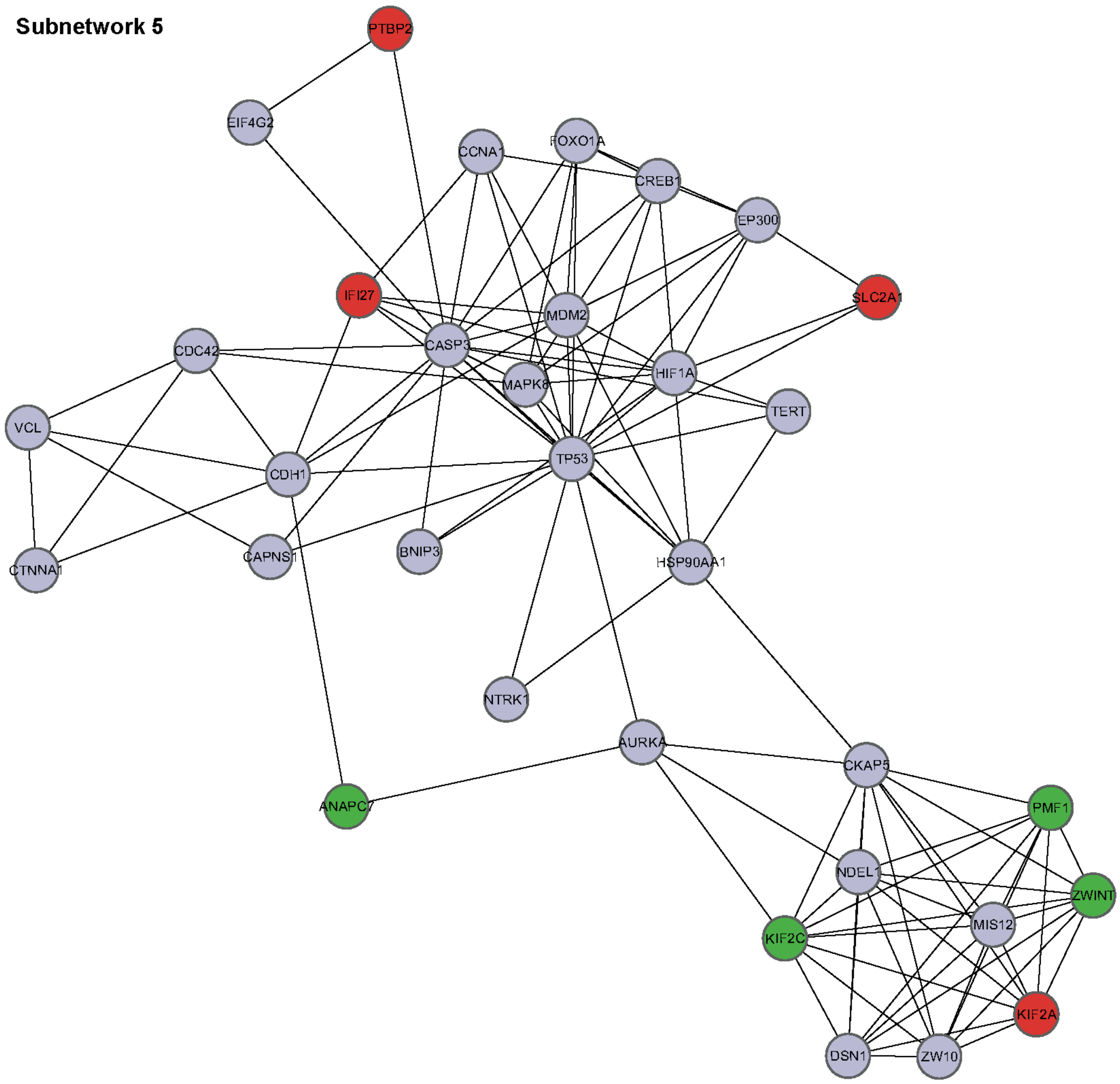

Supplementary Figure 2. Functional Association Network Analysis of proteins regulated >1.5-fold. One extra network node was allowed to study associations with proteins not regulated in the LC-MS/MS data set. (**A**) Functional association network prediction extracted from the STRING database. (**B**) MCODE algorithm and KEGG pathway analysis of the functional association network extracted five highly-connected subnetworks. Subnetwork 1 is shown in Supplementary Figure 3. Subnetwork 2 was enriched for proteins involved in focal adhesion, ECM-receptor interaction, cell adhesion molecules, regulations of actin cytoskeleton and insulin signaling receptor pathways, indicating that the regulated proteins in subnetwork 2 affect cell motility. Among others, the ErbB signaling pathway was enriched in subnetwork 3 and 4, which has been suggested to be involved in tamoxifen resistance. Green nodes represent proteins upregulated in TamR-1 vs. MCF-7/S0.5, red nodes represent downregulated proteins and blue nodes represent extra node predictions.

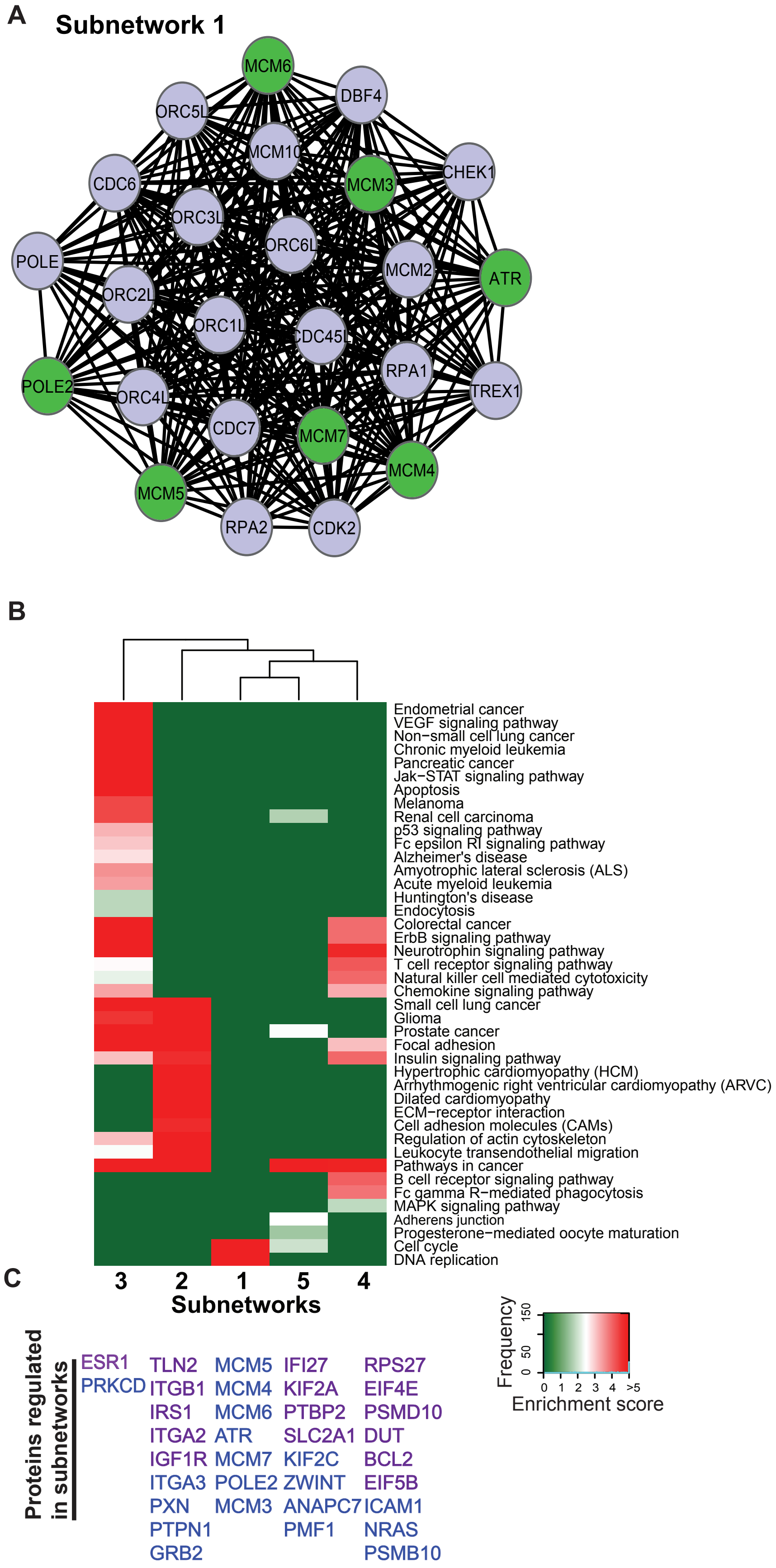
A
Subnetwork 1
MCM6
DBF4
ORC5L
MCM10
CDC6
MCM3
CHEK1
ORC3L
ORC6L
POLE
MCM2
ATR
ORC2L
ORC1L
CDC45L
RPA1
POLE2
ORC4L
TREX1
CDC7
MCM7
MCM4
MCM5
RPA2
CDK2
B
Endometrial cancer
VEGF signaling pathway
Non−small cell lung cancer
Chronic myeloid leukemia
Pancreatic cancer
Jak−STAT signaling pathway
Apoptosis
Melanoma
Renal cell carcinoma
p53 signaling pathway
Fc epsilon RI signaling pathway
Alzheimer's disease
Amyotrophic lateral sclerosis (ALS)
Acute myeloid leukemia
Huntington's disease
Endocytosis
Colorectal cancer
ErbB signaling pathway
Neurotrophin signaling pathway
T cell receptor signaling pathway
Natural killer cell mediated cytotoxicity
Chemokine signaling pathway
Small cell lung cancer
Glioma
Prostate cancer
Focal adhesion
Insulin signaling pathway
Hypertrophic cardiomyopathy (HCM)
Arrhythmogenic right ventricular cardiomyopathy (ARVC)
Dilated cardiomyopathy
ECM−receptor interaction
Cell adhesion molecules (CAMs)
Regulation of actin cytoskeleton
Leukocyte transendothelial migration
Pathways in cancer
B cell receptor signaling pathway
Fc gamma R−mediated phagocytosis
MAPK signaling pathway
Adherens junction
Progesterone−mediated oocyte maturation
Cell cycle
DNA replication
3 2 1 5 4
Subnetworks
Frequency
0 50 150
0 1 2 3 4 >5
Enrichment score
C
Proteins regulated in subnetworks
ESR1
PRKCD
TLN2
ITGB1
IRS1
ITGA2
IGF1R
ITGA3
PXN
PTPN1
GRB2
MCM5
MCM4
MCM6
ATR
MCM7
POLE2
MCM3
IFI27
KIF2A
PTBP2
SLC2A1
KIF2C
ZWINT
ANAPC7
PMF1
RPS27
EIF4E
PSMD10
DUT
BCL2
EIF5B
ICAM1
NRAS
PSMB10

Supplementary Figure 3. KEGG pathway analysis of 5 highly connected subnetworks. (**A**) MCODE algorithm and KEGG pathway analysis of the functional association network extracted five highly-connected subnetworks. The top network, containing several MCM proteins and ATR, is shown, while the other networks are depicted in Supplementary Fig. 2. Green nodes represent proteins upregulated in TamR-1 vs. MCF-7/S0.5, red nodes represent downregulated proteins and blue nodes represent extra node predictions. (**B**) Heatmap of pathways associated with the five major subnetworks were identified by KEGG pathway analysis. Color key indicates the pathway enrichment scores. (**C**) Some representative regulated proteins in each subnetwork. Protein names in blue and purple represent upregulation and downregulation, respectively, in TamR-1 vs. MCF-7/S0.5.

A

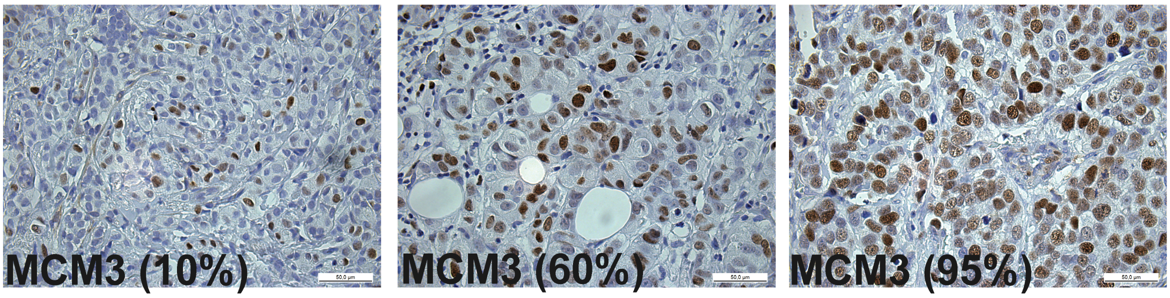

Supplementary Figure 4. (**A**) **Immunohistochemical staining of representative ER+ primary breast cancer tissue samples for MCM3.** MCM3 (% stained cells).

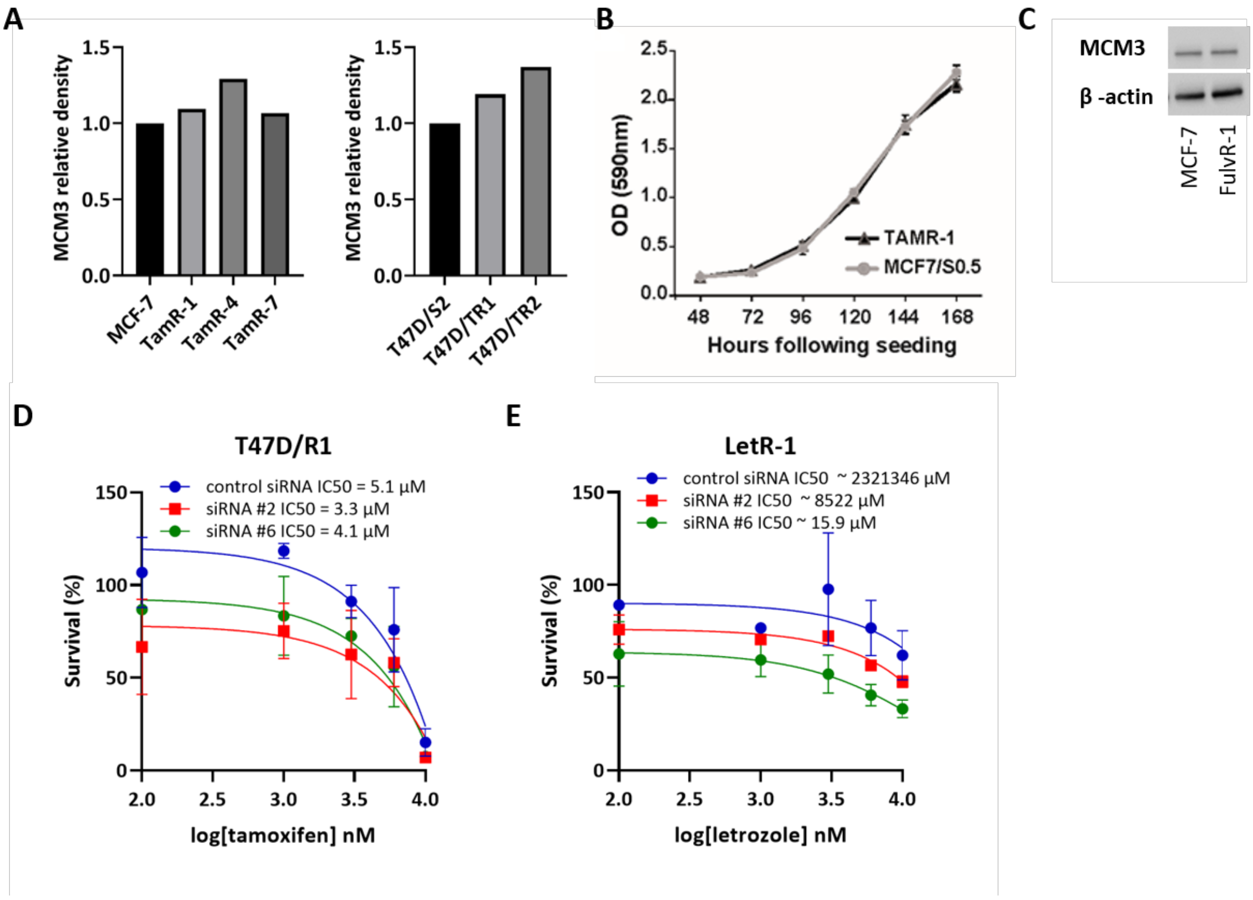

Supplementary Figure 5. (**A**) Band intensity of MCM3 in TamR vs. MCF-7/S0.5 cells and T47D/TR vs. T47D/S2 cells was quantified using ImageJ software and normalized to GAPDH and β-actin, respectively. (**B**) Cell growth analysis of TamR-1 and MCF-7/S0.5 cultured in standard conditions as measured by a colorimetric crystal violet assay. A representative experiment of two where data is represented as OD570 values ± s.e.m of 4 replicates is shown. (**C**) MCM3 expression in FulvR-1 vs. MCF-7/S0.5 cells by Western blotting using whole cell lysates. β-actin was used as loading control. A representative of two biological replicates is shown. T47D/R1 (**D**) and LetR-1 (**E**) cells were treated with different concentrations of tamoxifen (T47D/R1) or letrozole (LetR-1) for 5 days after MCM3 knockdown. Cell growth was measured by crystal violet colorimetric assay. Growth relative to vehicle (%) is plotted against log drug concentration. Each data point represents the average of three technical replicates.

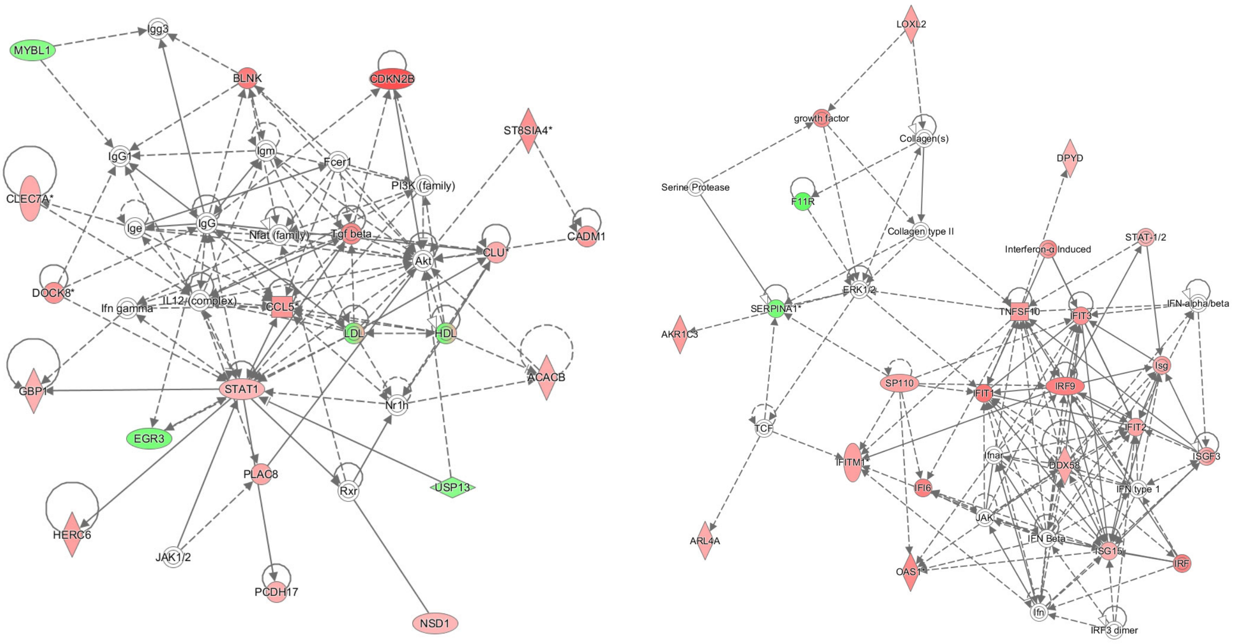

Supplementary Figure 6. The two top networks of genes exhibiting altered expression in MCM3 knockdown versus siControl TamR cells identified using Ingenuity Pathway analysis software. Gene names for the altered genes are indicated. Red = elevated levels, green = decreased levels in MCM3 knockdown versus siControl TamR cells.

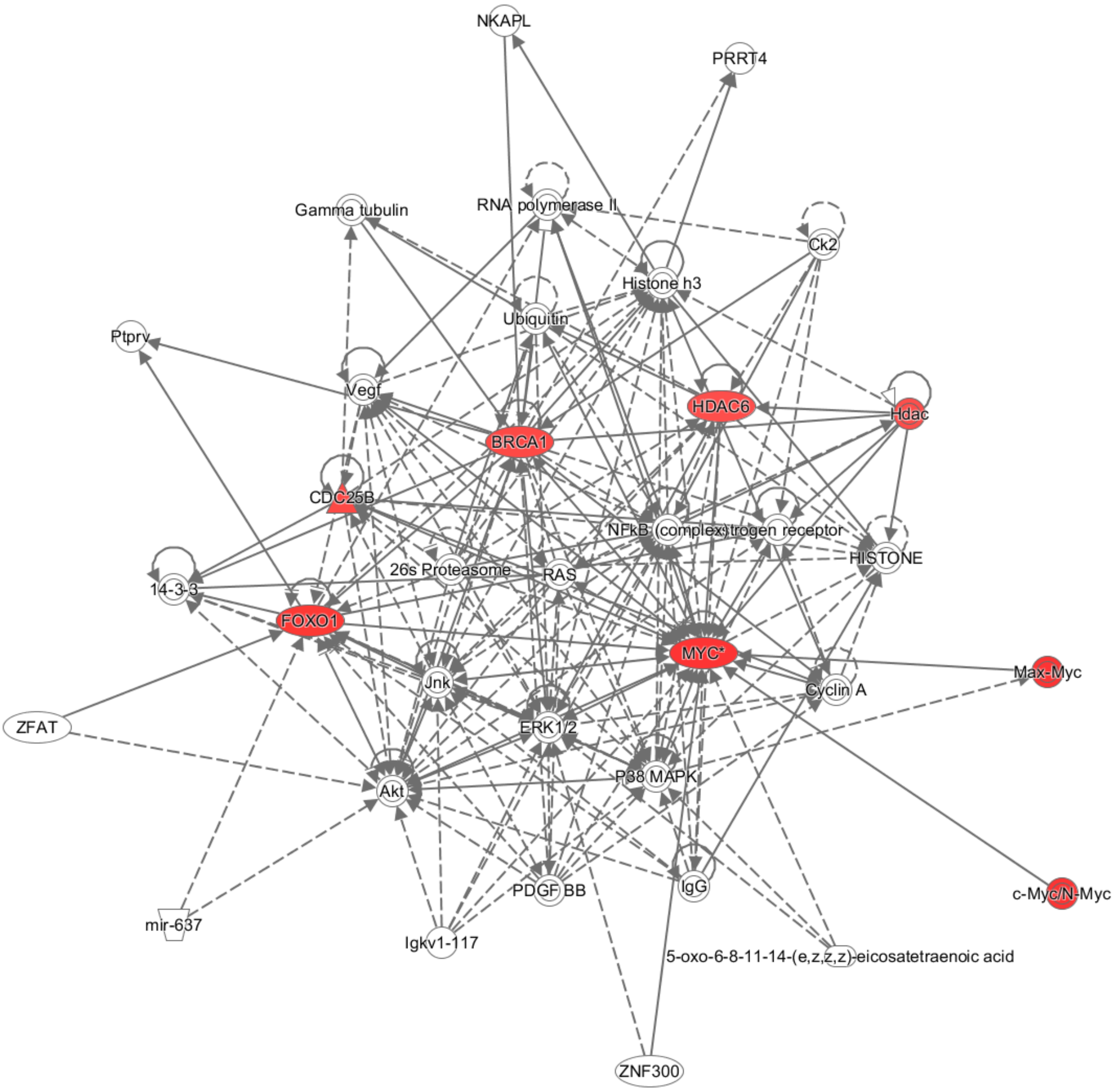

Supplementary Figure 7. Network of proteins exhibiting altered phosphorylation in MCM3 knockdown versus siControl TamR-1 cells identified using Ingenuity Pathway analysis software (only one was identified). Gene names of the proteins exhibiting altered phosphorylated are indicated. Red = elevated levels, green = decreased levels in MCM3 knockdown versus siControl TamR-1 cells.

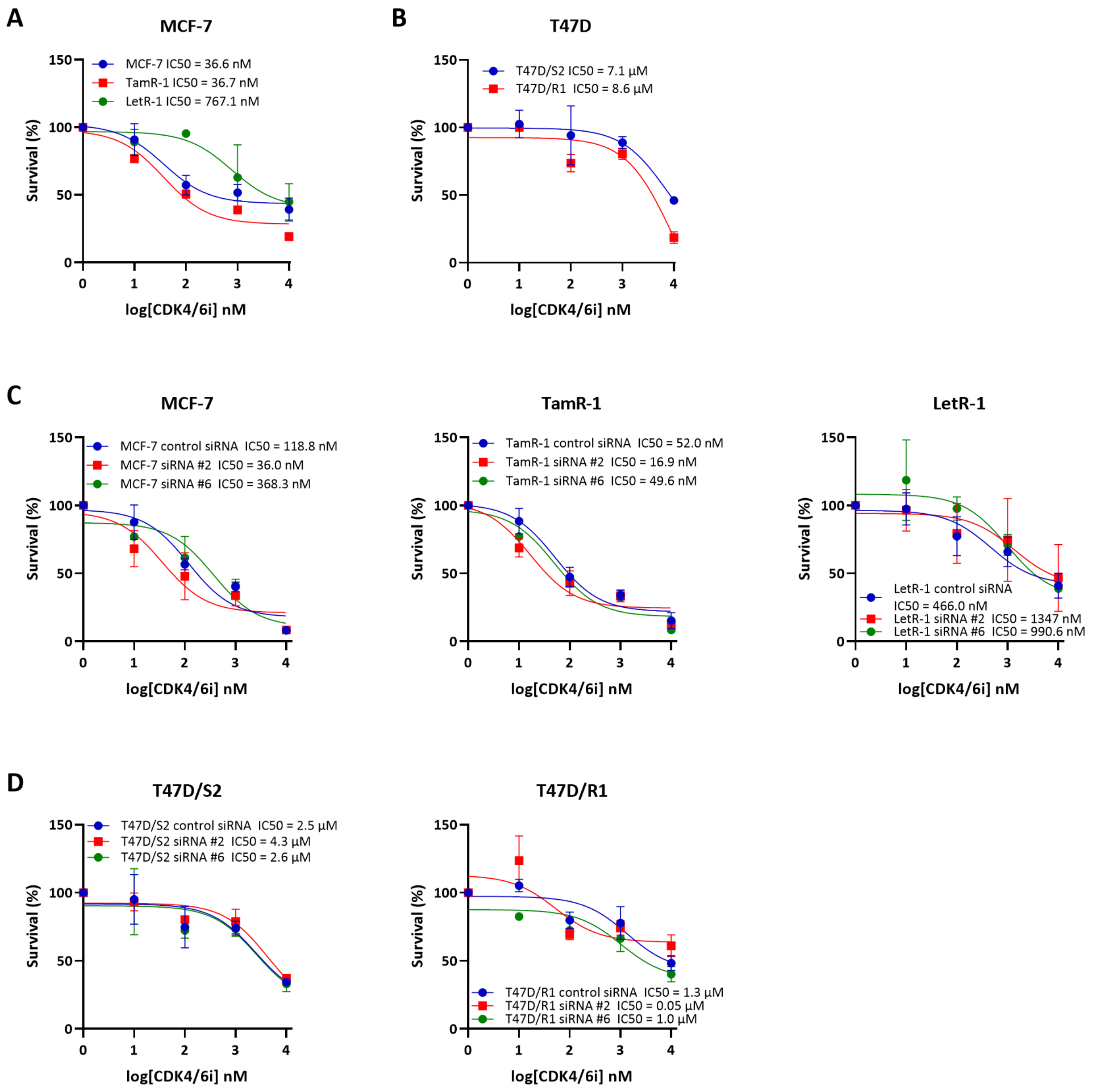

Supplementary Figure 8. Dose-dependent effect of CDK4/6 inhibitor in tamoxifen- and letrozole-resistat cell models. MCF-7 (MCF-7/S0.5), TamR-1, LetR-1, T47D/S2 and T47D/R1 cells were treated with different concentrations of the CDK4/6 inhibitor (CDK4/6i) palbociclib for 4 days without (**A** and **B**) or with (**C** and **D**) MCM3 knockdown. Cell growth was measured by crystal violet colorimetric assay. Growth relative to vehicle (%) is plotted against log drug concentration. Each data point represents the average of three technical replicates.

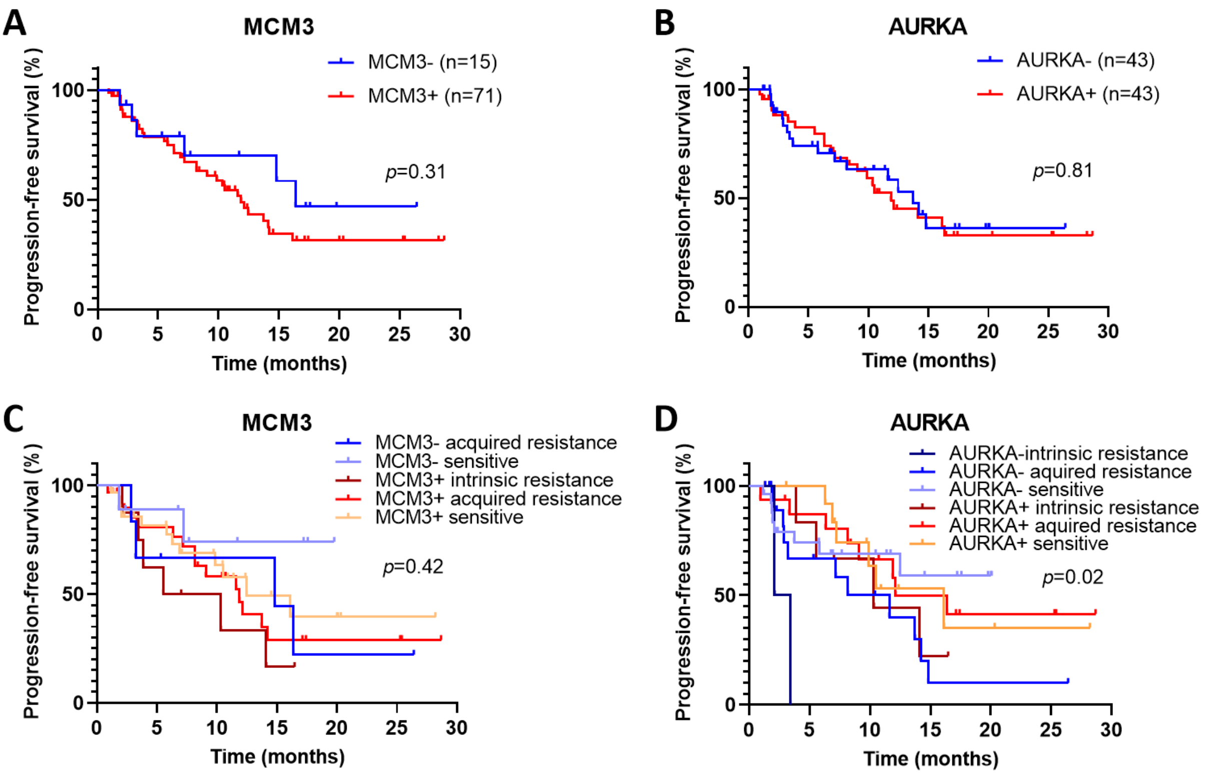

Supplementary Figure 9. MCM3 and AURKA expression in metastasis does not correlate with progression-free survival of advanced ER+ breast cancer patients treated with combined CDK4/6 inhibitor and endocrine therapy. Kaplan-Meier plots evaluating the association of MCM3 (**A** and **C**) and AURKA (**B** and **D**) expression and progression-free survival in a cohort of 86 ER+ advanced breast cancer patients treated with combined CDK4/6 inhibitor and endocrine therapy. The log-rank (Mantel-Cox) test was used to determine statistical significance. (**C** and **D**) For the subgroup analysis intrinsic endocrine resistance was defined as relapse during the first 2 years of adjuvant endocrine therapy, or progression within 6 months while receiving first-line endocrine therapy in the metastatic setting. Acquired endocrine resistance was defined as relapse after the first 2 years while on adjuvant endocrine therapy, or relapse within 12 months after the end of adjuvant endocrine therapy, or progression after at least 6 months while on endocrine therapy for metastatic disease. Endocrine sensitive was defined as relapse at least 12 months after the completion of neoadjuvant or adjuvant endocrine therapy, or no treatment for metastatic disease.

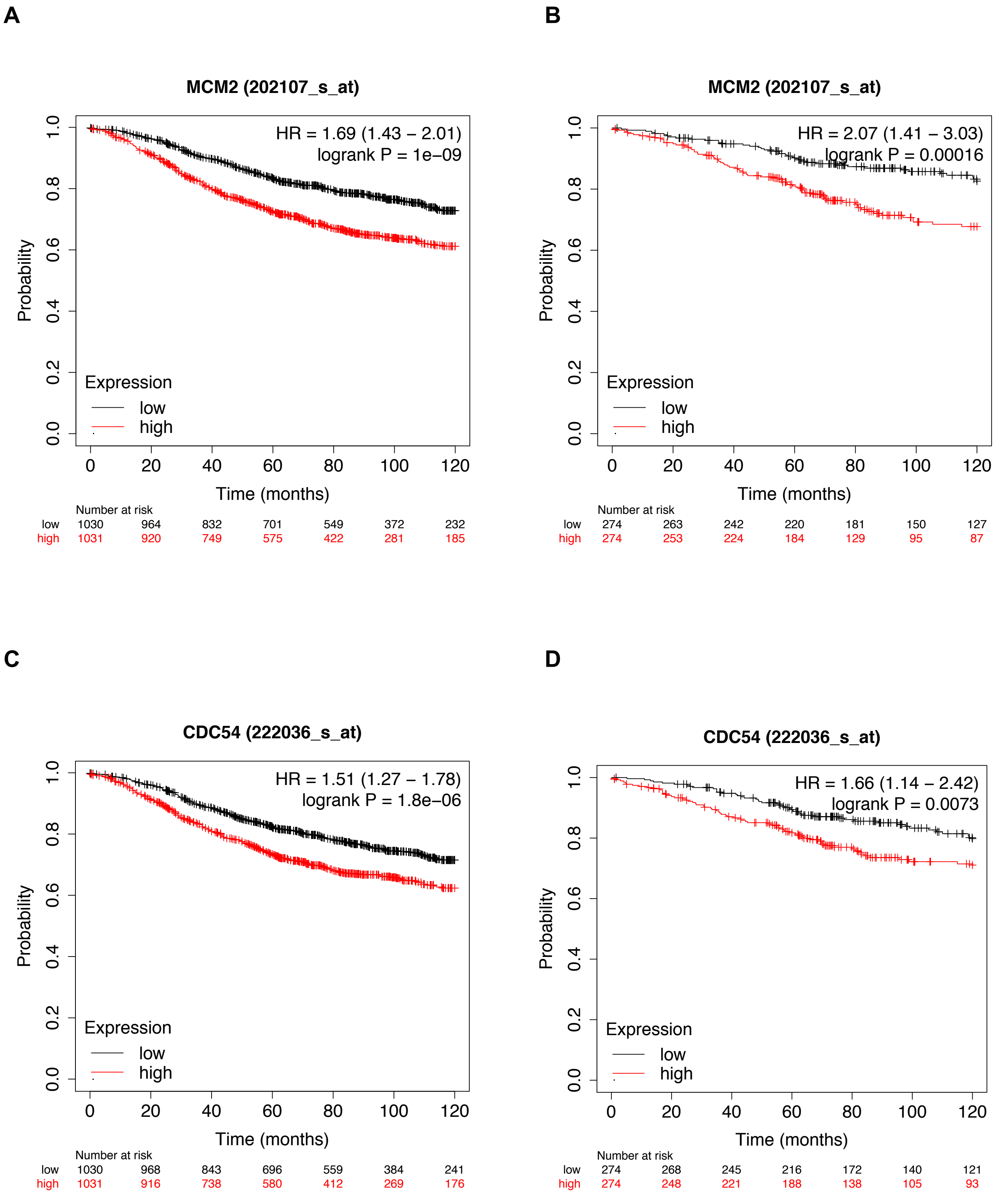

Supplementary Figure 10. Kaplan-Meier plots showing the association of MCM2 (**A** and **B**) and MCM4 (CDC54) (**C** and **D**) gene expression with 10-year recurrence-free survival (RFS) (**A** and **C**) and overall survival (OS) (**B** and **D**) in cohort 3 of early-stage, ER^+ breast cancer patients treated with adjuvant endocrine treatment based on gene array data obtained from KMplot.com.

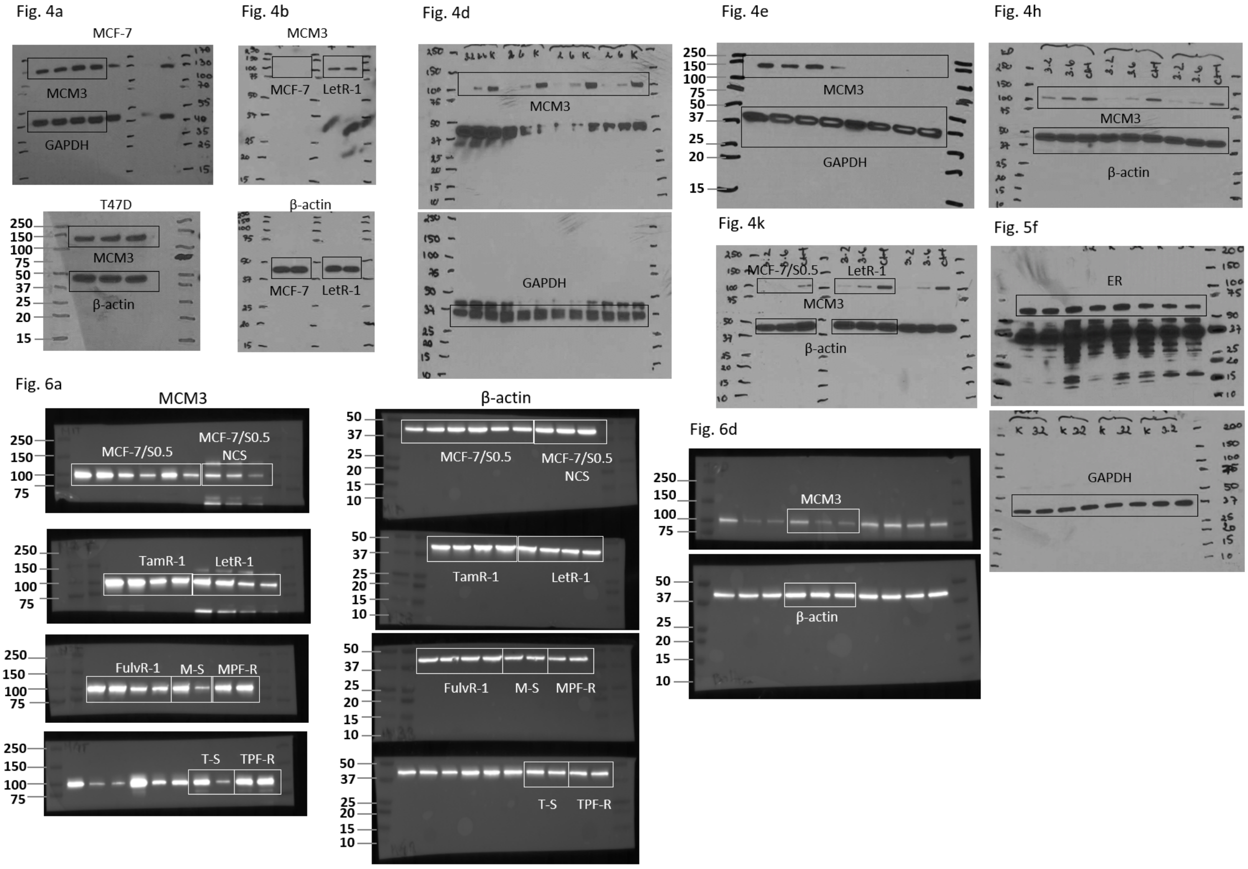

Supplementary Figure 11. Uncropped images of all Western blots shown in the main figures. Uncropped full scans of Western blots from the corresponding cropped Western blots shown in the main figures. Figure subpanel and molecular markers are indicated.

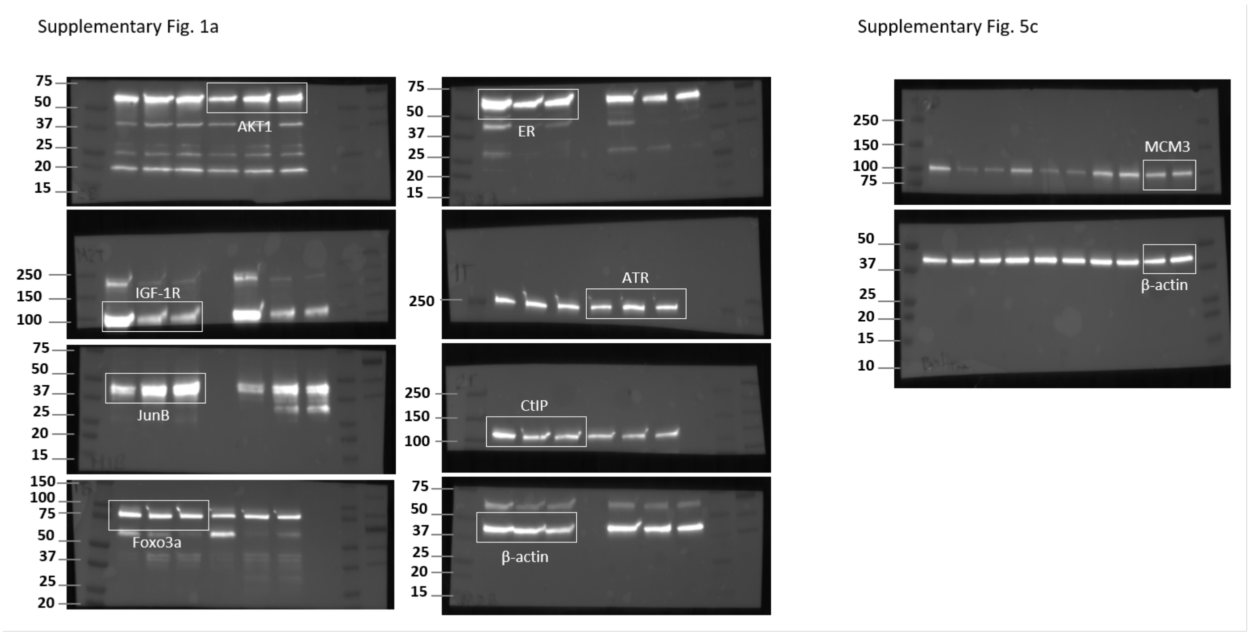

Supplementary Figure 12. Uncropped images of all Western blots shown in the Supplementary Figures. Uncropped full scans of Western blots from the corresponding cropped Western blots shown in the Supplementary Figures. Figure subpanel and molecular markers are indicated.

Supplementary Table 1. List of the 275 proteins showing increased expression, and 264 showed reduced expression in TAM^R-1 vs. MCF-7/S0.5 cells defined as ≥1.5-fold differential expression.

Protein names	Gene names	Uniprot accession no.	Numbers of peptides used for quantification	Sequence Coverage (%)	Expression level (TAMR1 vs. MCF-7/S0.5)
**SH3 domain-binding glutamic acid-rich-like protein**	SH3BGRL	O75368	12	70.2	15.2
**Neurosecretory protein VGF**	VGF	Q9UDW8	7	8.5	11.0
**Glial fibrillary acidic protein**	GFAP	P14136	2	8.2	8.2
**Kynureninase**	KYNU	Q16719	14	25.2	7.3
**cDNA FLJ76744**	L1CAM	A8K139	5	2.9	6.1
**Heat shock-related 70 kDa protein 2**	HSPA2	P54652	10	25.8	5.2
**Ladinin-1**	LAD1	O00515	5	12.6	5.2
**SET domain-containing protein 5**	SETD5	Q9C0A6	2	1.2	4.6
**Actin-binding protein anillin**	ANLN	A8MUK7	4	5.7	4.5
**Calcium-binding protein 39-like**	CAB39L	Q9H9S4	9	8.3	4.4
**Glycerophosphodiester phosphodiesterase domain-containing protein 1**	GDPD1	Q8N9F7	3	8	4.3
**Serine beta-lactamase-like protein LACTB, mitochondrial**	LACTB	P83111	19	21.4	4.2
**Glutathione S-transferase Mu 4**	GSTM4	Q03013	11	25.2	3.8
**LIM and calponin homology domains-containing protein 1**	LIMCH1	Q9UPQ0	4	2.7	3.5
**LIM and cysteine-rich domains protein 1**	LMCD1	Q9NZU5	4	6.4	3.4
**Protein dpy-19 homolog 1**	DPY19L1	Q2PZI1	2	3	3.4
**Glycogen phosphorylase**	PYGL	P06737	15	21.3	3.3
**Acetyl-coenzyme A synthetase 2-like, mitochondrial**	ACSS1	Q9NUB1	11	14.2	3.0
**Fructose-bisphosphate aldolase C**	ALDOC	P09972	7	41.8	3.0
**Short-chain specific acyl-CoA dehydrogenase**	ACADS	P16219	6	21.6	2.9
**Ankyrin repeat and SOCS box protein 9**	ASB9	Q96DX5	6	16.7	2.9

**Kelch repeat and BTB domain-containing protein 2**	KBTBD2	Q8IY47	2	4.5	2.9
**Cytochrome P450 1B1**	CYP1B1	Q16678	2	7.2	2.9
**LIM domain only protein 7**	LMO7	Q8WWI1	4	3.3	2.9
**Sarcoplasmic/endoplasmic reticulum calcium ATPase 3**	ATP2A3	Q93084	7	12.9	2.7
**Mevalonate kinase**	MVK	Q03426	4	18.2	2.6
**Late secretory pathway protein AVL9 homolog**	AVL9	Q8NBF6	8	13.7	2.6
**Mitochondrial glutamate carrier 2**	SLC25A18	Q9H1K4	4	15.9	2.6
**Vacuolar protein sorting-associated protein 41 homolog**	VPS41	P49754	4	2.7	2.6
**Tensin-3**	TNS3	Q68CZ2	7	8.4	2.6
**Latexin**	LXN	Q9BS40	4	8.1	2.5
**NEDD4-binding protein 3**	N4BP3	O15049	2	4.6	2.5
**SH2 domain-containing protein 4A**	SH2D4A	Q9H788	2	7.3	2.5
**Proteasome subunit beta type-8**	PSMB8	P28062	3	13.8	2.5
**Transcription factor jun-B**	JUNB	P17275	11	42.7	2.4
**Paralemmin-3**	PALM3	A6NDB9	7	4.8	2.4
**cDNA FLJ78458**	"SEPT10;"	A8K7M3	6	7.7	2.3
**Lysosome membrane protein 2**	SCARB2	Q14108	13	14.6	2.3
**Dynamin 1 isoform 2 variant**	DNM1	Q05193	3	3.5	2.3
**Myosin-XVIIIa**	MYO18A	Q92614	10	4.8	2.3
**Cysteine and glycine-rich protein 1**	CSRP1	P21291	15	36.8	2.2
**Canalicular multispecific organic anion transporter 2**	ABCC3	O15438	19	11.7	2.2
**Brain acid soluble protein 1**	BASP1	P80723	8	36.6	2.2
**Cleavage stimulation factor 64 kDa subunit**	CSTF2T	Q9H0L4	2	19.8	2.2
**V-type proton ATPase 116 kDa subunit a isoform 3**	TCIRG1	Q13488	2	1.4	2.2
**Protein LSM14 homolog B**	LSM14B	Q9BX40	2	28.8	2.2
**GTPase Nras**	NRAS	P01111	17	39.7	2.1
**Transforming acidic coiled-coil-containing protein 1**	TACC1	O75410	6	8	2.1
**Cingulin**	CGN	Q9P2M7	13	9.1	2.1
**Inositol monophosphatase**	IMPA1	P29218	2	10.8	2.1
**Delta-aminolevulinic acid dehydratase**	ALAD	A8K375	6	9.7	2.1
**Band 4.1-like protein 2**	EPB41L2	O43491	25	15.9	2.1

**KDEL motif-containing protein 2**	KDELC2	Q7Z4H8	10	4.7	2.1
**Cathepsin H**	CTSH	P09668	2	7.8	2.1
**Neurobeachin**	NBEA	Q8NFP9	4	3.4	2.0
**Tight junction protein ZO-2**	TJP2	Q9UDY2	18	14.3	2.0
**Nuclear autoantigen Sp-100**	SP100	P23497	3	5	2.0
**Zinc finger protein 281**	ZNF281	Q9Y2X9	6	10.5	2.0
**Plastin-2**	LCP1	P13796	13	23.8	2.0
**Butyrate response factor 2**	ZFP36L2	P47974	2	6.6	2.0
**Intercellular adhesion molecule 1**	ICAM1	P05362	4	6	2.0
**Mitochondrial carnitine/acylcarnitine carrier protein CACL**	SLC25A29	Q8N8R3	6	17.2	2.0
**Inositol-tetrakisphosphate 1-kinase**	ITPK1	Q13572	10	25.1	2.0
**Choline transporter-like protein 2**	SLC44A2	Q8IWA5	4	3.1	2.0
**Synaptotagmin-like protein 2**	SYTL2	Q9HCH5	5	9	2.0
**Dephospho-CoA kinase domain-containing protein**	DCAKD	Q8WVC6	5	10.8	2.0
**Tudor and KH domain-containing protein**	TDRKH	Q9Y2W6	6	9.4	2.0
**Zinc finger CCCH-type with G patch domain-containing protein**	ZGPAT	Q8N5A5	3	10.9	2.0
**Cytochrome b561**	CYB561	P49447	2	5.1	2.0
**DnaJ homolog subfamily C member 5**	DNAJC5	Q9H3Z4	4	29.3	2.0
**ADP-ribosylation factor-related protein 1**	ARFRP1	Q13795	7	21.9	2.0
**Squalene synthetase**	FDFT1	P37268	11	15.3	2.0
**Putative uncharacterized protein YIF1A**	YIF1A	A6NM00	3	4	2.0
**DNA polymerase epsilon subunit 2**	POLE2	P56282	3	7	2.0
**Histone H1.0**	H1F0	P07305	6	13.9	2.0
**Electron transfer flavoprotein-ubiquinone oxidoreductase**	ETFDH	Q16134	6	17.7	1.9
**N-acetyl-D-glucosamine kinase**	NAGK	Q9UJ70	24	35.8	1.9
**Fumarylacetoacetate hydrolase domain-containing protein 2B**	FAHD2B	Q6P2I3	2	26.4	1.9
**Mitogen-activated protein kinase kinase kinase MLT**	MLTK	Q9NYL2	4	7.5	1.9
**Agmatinase, mitochondrial**	AGMAT	Q9BSE5	5	19	1.9

**Protein LSM14 homolog B**	LSM14B	Q9BX40	16	26.5	1.9
**cDNA FLJ78091**	UHRF1	A8K024	7	9.8	1.9
**Methyl-CpG-binding domain protein 3**	MBD3	O95983	4	18.9	1.9
**Diacylglycerol kinase alpha**	DGKA	P23743	2	3	1.9
**Dehydrogenase/reductase SDR family member 7B**	DHRS7B	Q6IAN0	6	17.8	1.9
**GMP reductase 1**	GMPR	P36959	2	13.9	1.9
**Nucleoside diphosphate kinase 3**	NME3	Q13232	8	30.8	1.9
**Delta(14)-sterol reductase**	TM7SF2	O76062	2	3.1	1.9
**Probable hydrolase PNKD**	PNKD	Q8N490	4	17.1	1.9
**Pyridoxine-5'-phosphate oxidase**	PNPO	Q9NVS9	14	29.9	1.9
**Cellular retinoic acid-binding protein 2**	CRABP2	P29373	7	34.8	1.9
**NADH-cytochrome b5 reductase 1**	CYB5R1	Q9UHQ9	13	27.2	1.9
**FYVE and coiled-coil domain-containing protein 1**	FYCO1	Q9BQS8	2	1.6	1.9
**Solute carrier family 25 member 42**	SLC25A42	Q86VD7	2	5.3	1.9
**Zinc finger protein 644**	ZNF644	Q9H582	2	1.3	1.9
**ATP-dependent DNA helicase Q5**	RECQL5	O94762	2	2	1.8
**Coactosin-like protein**	COTL1	Q14019	2	23.9	1.8
**Cystathionine beta-synthase**	CBS	P35520	3	6.5	1.8
**"Epsin-3;EPS-15-interacting protein 3"**	EPN3	Q9H201	23	11.6	1.8
**T-box transcription factor TBX2**	TBX2	Q13207	3	5.6	1.8
**Protein kinase C delta type**	PRKCD	Q05655	12	13.3	1.8
**Neurabin-2**	PPP1R9B	Q96SB3	4	7.1	1.8
**FK506-binding protein 9**	FKBP9	O95302	2	4.6	1.8
**Exocrine differentiation and proliferation factor**	EXDPF	Q4VXP1	5	35	1.8
**Lysophosphatidic acid phosphatase type 6**	ACP6	Q9NPH0	4	9.8	1.8
**Amine oxidase [flavin-containing] A**	MAOA	P21397	2	3	1.8
**Acetyl-CoA acetyltransferase, cytosolic**	ACAT2	Q9BWD1	38	37.3	1.8
**Dehydrogenase/reductase SDR family member on chromosome X**	DHRSX	Q8N5I4	3	4.5	1.8
**Signal-transducing adaptor protein 2**	STAP2	A6NKK3	7	12.7	1.8

**cDNA FLJ75329**	LASS2	A8K4M4	5	11.2	1.8
**Putative uncharacterized protein MAP7**	MAP7	A8MPQ8	12	21.8	1.8
**OGFR protein**	OGFR	Q05BV5	11	19.5	1.8
**Polyamine-modulated factor 1**	PMF1	Q5TCK2	2	6.6	1.8
**Putative uncharacterized protein DKFZp686N1969**	DKFZp686 N1969	Q68E09	4	1.4	1.8
**DNA polymerase epsilon catalytic subunit isoform a**	POLE1	Q9UNE9	2	1.2	1.8
**Epidermal growth factor receptor kinase substrate 8-like protein 2**	EPS8L2	Q9H6S3	6	4.2	1.8
**Guanine nucleotide-binding protein subunit alpha-13**	GNA13	Q14344	19	22.8	1.8
**DNA replication licensing factor MCM4**	MCM4	P33991	39	22.1	1.8
**UDP-N-acetylhexosamine pyrophosphorylase-like protein 1**	UAP1L1	Q3KQV9	5	18.1	1.8
**Gamma-synuclein**	SNCG	O76070	5	26	1.8
**Fructose-1,6-bisphosphatase 1**	FBP1	P09467	79	44.4	1.8
**Putative pre-mRNA-splicing factor ATP-dependent RNA helicase DHX32**	DHX32	Q7L7V1	10	9	1.8
**RNA-binding motif, single-stranded-interacting protein 1**	RBMS1	Q14869	6	17.2	1.8
**3-keto-steroid reductase**	HSD17B7	P56937	3	7	1.8
**3-hydroxybutyrate dehydrogenase type 2**	BDH2	Q9BUT1	5	24.1	1.8
**Histone chaperone ASF1B**	ASF1B	Q9NVP2	2	9.9	1.7
**Chitinase domain-containing protein 1**	CHID1	Q9BWS9	4	7.9	1.7
**Ribosome maturation protein SBDS**	SBDS	Q9Y3A5	5	10.8	1.7
**Lamin-B receptor**	LBR	Q14739	2	3.1	1.7
**Uncharacterized protein C20orf112**	C20orf11 2	Q96MY1	2	6.9	1.7
**Huntingtin-interacting protein 1**	HIP1	O00291	2	4.6	1.7
**[Pyruvate dehydrogenase [lipoamide]] kinase isozyme 2, mitochondrial**	PDK2	Q15119	4	7	1.7
**Toll interacting protein variant**	TOLLIP	Q59FB9	7	19.3	1.7
**"Cathepsin B;Cathepsin B1"**	CTSB	P07858	12	23.6	1.7
**Elongation factor 1-alpha 2**	EEF1A2	Q05639	115	66.3	1.7

**Vesicle transport through interaction with t-SNAREs homolog 1B**	VTI1B	Q9UEU0	2	10.3	1.7
**Zinc finger protein 512B**	ZNF512B	Q96KM6	4	9.2	1.7
**Huntingtin-interacting protein 1-related protein**	HIP1R	O75146	26	21	1.7
**Plasma membrane calcium-transporting ATPase 1**	ATP2B1	P20020	21	14.8	1.7
**Prosaposin;Proactivator polypeptide;Saposin-A**	PSAP	B1AVU8	27	12.2	1.7
**Glycolipid transfer protein**	GLTP	Q9NZD2	7	29.2	1.7
**Lysine-specific demethylase 2A**	KDM2A	Q9Y2K7	8	8.1	1.7
**Eukaryotic translation initiation factor 4E-binding protein 2**	EIF4EBP2	Q13542	2	43.3	1.7
**Glycerophosphodiester phosphodiesterase domain-containing protein 3**	GDPD3	Q7L5L3	3	9.4	1.7
**Lanosterol 14-alpha demethylase**	CYP51A1	Q16850	12	23.4	1.7
**Lysine-specific demethylase 5B**	KDM5B	Q9UGL1	5	3.7	1.7
**Probable saccharopine dehydrogenase**	SCCPDH	Q8NBX0	48	35	1.7
**Breast cancer-associated antigen SGA-72M**	SGA72M	Q6TV07	12	8.4	1.7
**Xaa-Pro aminopeptidase 1**	XPNPEP1	Q9NQW7	8	7.2	1.7
**Cytosolic purine 5'-nucleotidase**	NT5C2	P49902	8	19.4	1.7
**Beta-arrestin-1**	ARRB1	P49407	7	16.1	1.7
**Uncharacterized protein LOC113230**		Q96FF7	2	5.3	1.7
**E3 ubiquitin-protein ligase TRIM33**	TRIM33	Q9UPN9	46	20.4	1.7
**Clathrin light chain B**	CLTB	P09497	3	8.7	1.7
**Utrophin**	UTRN	P46939	6	1.6	1.7
**Adenylate cyclase type 3**	ADCY3	O60266	2	2.4	1.7
**Peroxisomal proliferator-activated receptor A-interacting complex 285 kDa protein**	PRIC285	Q9BYK8	32	11.1	1.7
**Liprin-beta-1**	PPFIBP1	Q86W92	10	1.7	1.7
**Spectrin beta chain, brain 2**	SPTBN2	O15020	38	14.6	1.7
**Probable aminopeptidase NPEPL1**	NPEPL1	Q8NDH3	13	27.5	1.6
**Mitochondrial aspartate-glutamate carrier protein**	SLC25A13	Q546F9	35	36.7	1.6

**Dual specificity protein phosphatase 3**	DUSP3	P51452	6	26.2	1.6
**Phosphotriesterase-related protein**	PTER	Q96BW5	4	13.5	1.6
**TOM1-like protein 2**	TOM1L2	Q6ZVM7	2	11.8	1.6
**Long-chain-fatty-acid--CoA ligase 1**	ACSL1	P33121	6	8	1.6
**Tyrosine-protein phosphatase non-receptor type 1**	PTPN1	P18031	31	39.5	1.6
**Putative uncharacterized protein CIT**	CIT	A8MWQ3	2	1.4	1.6
**Transmembrane protein C3orf1**	C3orf1	Q9NPL8	3	13	1.6
**DNA replication licensing factor MCM3**	MCM3	P25205	34	24.5	1.6
**Probable ATP-dependent RNA helicase DDX60**	DDX60	Q8IY21	48	23.4	1.6
**DNA replication licensing factor MCM7**	MCM7	P33993	38	29.1	1.6
**Non-specific lipid-transfer protein**	SCP2	P22307	29	33.3	1.6
**Adenylyltransferase and sulfurtransferase MOCS3**	MOCS3	O95396	12	17.2	1.6
**Thymidine kinase, cytosolic**	TK1	P04183	7	12	1.6
**Glutaryl-CoA dehydrogenase, mitochondrial**	GCDH	Q92947	18	37.4	1.6
**Vacuolar protein sorting-associated protein 18 homolog**	VPS18	Q9P253	6	7.9	1.6
**RILP-like protein 1**	RILPL1	Q5EBL4	2	9.7	1.6
**L-xylulose reductase**	DCXR	Q7Z4W1	32	41.8	1.6
**WD repeat-containing protein 68**	WDR68	P61962	4	14	1.6
**cAMP-dependent protein kinase type I-alpha regulatory subunit**	PRKAR1A	P10644	18	18.4	1.6
**Carrier family 6 , member 8 variant**	PPGB	Q59EV6	11	9.8	1.6
**Kinesin-like protein KIF16B**	KIF16B	Q96L93	3	4.3	1.6
**DKFZp761E198 protein**	DKFZp761 E198	Q0D2Q2	4	1.5	1.6
**Sterol O-acyltransferase 1**	SOAT1	P35610	8	11.3	1.6
**Kinesin-like protein KIF2C**	KIF2C	Q99661	4	8.3	1.6
**Proteasome subunit beta type-10**	PSMB10	P40306	4	24.9	1.6
**Protein prune homolog**	PRUNE	Q86TP1	3	12.1	1.6
**PCTAIRE protein kinase 3**	PCTK3	Q5VXQ4	2	9.3	1.6

**Methylated-DNA--protein-cysteine methyltransferase**	MGMT	P16455	15	17.4	1.6
**Diphosphoinositol polyphosphate phosphohydrolase 2**	NUDT4	Q9NZJ9	2	19.9	1.6
**N(G),N(G)-dimethylarginine dimethylaminohydrolase 2**	DDAH2	O95865	10	48.1	1.6
**Acetoacetyl-CoA synthetase**	AACS	Q86V21	4	6.1	1.6
**Ubiquitin-conjugating enzyme E2 variant 1**	UBE2V1	Q13404	11	11.6	1.6
**3,2-trans-enoyl-CoA isomerase, mitochondrial**	DCI	P42126	14	33.1	1.6
**Elongation factor 2 kinase**	EEF2K	O00418	2	1.4	1.6
**Beta-1,4-galactosyltransferase 1**	B4GALT1	P15291	8	7.5	1.6
**2'-5' oligoadenylate synthetase 1 p52 isoform**	OAS1	Q3ZM02	12	18.7	1.6
**Probable ATP-dependent RNA helicase DDX60-like**	DDX60L	Q5H9U9	2	3.2	1.6
**cDNA FLJ78682**	OAS3	A8KA84	62	28.4	1.6
**Vesicular integral-membrane protein VIP36**	LMAN2	Q12907	9	23.3	1.6
**Absent in melanoma 1 protein**	AIM1	Q9Y4K1	9	6.6	1.6
**DNA replication licensing factor MCM5**	MCM5	P33992	56	33.2	1.6
**Interferon regulatory factor 9**	IRF9	Q00978	7	10.2	1.6
**Putative N-acetylglucosamine-6-phosphate deacetylase**	AMDHD2	Q9Y303	6	18.9	1.6
**Vacuolar protein sorting-associated protein 16 homolog**	VPS16	Q9H269	11	10.4	1.6
**Mediator of RNA polymerase II transcription subunit 13**	MED13	Q9UHV7	5	3.6	1.6
**Mitofusin-1**	MFN1	Q8IWA4	2	3.3	1.6
**Microsomal glutathione S-transferase 1**	MGST1	P10620	14	30.3	1.6
**Ras-related protein Rab-7L1**	RAB7L1	O14966	3	24.1	1.6
**Ras-related protein Rab-9A**	RAB9A	P51151	9	23.4	1.6
**Chromodomain-helicase-DNA-binding protein 3**	CHD3	Q12873	3	3.9	1.6
**Putative uncharacterized protein KIF23**	KIF23	A8MTR8	4	6.6	1.6
**Putative phospholipase B-like 2**	PLBD2	Q8NHP8	5	6.3	1.6

**Aspartate beta-hydroxylase domain-containing protein 1**	ASPHD1	Q5U4P2	10	6.7	1.6
**Hepatocellular carcinoma-associated antigen 90**	HCA90	Q96RR5	5	8	1.6
**Glucosidase 2 subunit beta**	PRKCSH	P14314	34	30.3	1.6
**A-Raf proto-oncogene serine/threonine-protein kinase**	ARAF	P10398	3	14.2	1.6
**Presqualene diphosphate phosphatase**	PPAPDC2	Q8IY26	2	3.4	1.6
**Ectonucleotide pyrophosphatase/phospho diesterase family member 1**	ENPP1	P22413	4	7.4	1.6
**Trans-acting T-cell-specific transcription factor GATA-3**	GATA3	P23771	6	13.3	1.6
**Arf-GAP with SH3 domain, ANK repeat and PH domain-containing protein 2**	ASAP2	O43150	8	7.2	1.6
**Monoacylglycerol lipase ABHD12**	ABHD12	Q8N2K0	10	25.7	1.6
**Anaphase-promoting complex subunit 7**	ANAPC7	Q9UJX3	10	17.3	1.6
**Cytoplasmic aconitate hydratase**	ACO1	P21399	31	25.8	1.6
**Splicing factor, arginine/serine-rich 9**	SFRS9	Q13242	10	34.8	1.6
**Target of myb1 (Chicken)**	TOM1	Q86X74	3	8.7	1.6
**RAC-alpha serine/threonine-protein kinase**	AKT1	P31749	5	12.3	1.6
**Nuclear factor NF-kappa-B p100 subunit**	NFKB2	Q00653	11	11.7	1.6
**Caskin-2**	CASKIN2	Q8WXE0	4	4.6	1.6
**Kinesin-like protein KIF11**	KIF11	P52732	5	4.5	1.6
**Vam6/Vps39-like protein**	VPS39	Q96JC1	5	4.5	1.6
**DNA replication licensing factor MCM6**	MCM6	Q14566	25	19.2	1.6
**Paxillin**	PXN	P49023	10	25	1.6
**E3 ubiquitin-protein ligase UHRF2**	UHRF2	Q96PU4	2	4	1.5
**Importin-9**	IPO9	Q96P70	32	24.7	1.5
**F-box/LRR-repeat protein 12**	FBXL12	Q9NXK8	2	6.1	1.5
**High mobility group protein HMG-I/HMG-Y**	HMGA1	P17096	7	9.4	1.5
**Probable cation-transporting ATPase 13A1**	ATP13A1	Q9HD20	28	15.8	1.5
**Ezrin**	EZR	P15311	48	36.7	1.5

**Polyhomeotic-like protein 1**	PHC1	P78364	7	14.7	1.5
**Lanosterol synthase**	LSS	P48449	13	10.4	1.5
**cDNA FLJ77959**	PRKAR1B	A8K099	2	4.7	1.5
**Mitogen-activated protein-binding protein-interacting protein**	ROBLD3	Q9Y2Q5	4	40.8	1.5
**UDP-glucose 6-dehydrogenase**	UGDH	O60701	42	54.3	1.5
**Cytochrome c oxidase assembly protein COX19**	COX19	Q49B96	5	21.1	1.5
**Cold-inducible RNA-binding protein**	CIRBP	Q14011	8	14.8	1.5
**WD repeat-containing protein 25**	WDR25	Q64LD2	3	4.4	1.5
**Serum paraoxonase/arylesterase 2**	PON2	Q15165	6	14	1.5
**Serine/threonine-protein kinase ATR**	ATR	Q13535	4	1.4	1.5
**F-box only protein 21**	FBXO21	O94952	4	5.9	1.5
**TEA domain family member 3**	TEAD3	Q7Z6U8	2	6.2	1.5
**Methylcrotonoyl-CoA carboxylase subunit alpha, mitochondrial**	MCCC1	Q96RQ3	35	35.4	1.5
**Phosphatidylinositol 4-kinase beta**	PI4KB	Q5VWB9	3	2.8	1.5
**Tumor suppressor candidate gene 1 protein**	TUSC1	Q2TAM9	2	10.8	1.5
**Chromosome-associated kinesin KIF4A**	KIF4A	O95239	4	7.1	1.5
**Cytochrome P450 20A1**	CYP20A1	Q6UW02	2	5.4	1.5
**Uncharacterized protein C1orf103**	C1orf103	Q5T3J3	2	3.9	1.5
**Lethal(2) giant larvae protein homolog 2**	LLGL2	Q6P1M3	36	20.8	1.5
**PKHD domain-containing transmembrane protein C17orf101**	C17orf10 1	Q6PK18	6	11.9	1.5
**Surfeit locus protein 2**	SURF2	Q15527	2	7.4	1.5
**3-beta-hydroxysteroid-Delta(8),Delta(7)-isomerase**	EBP	Q15125	3	5.2	1.5
**Serine/threonine-protein kinase haspin**	GSG2	Q8TF76	2	5.6	1.5
**Protein HIRA**	HIRA	P54198	3	3.5	1.5
**HEAT repeat-containing protein C7orf27**	C7orf27	Q6PJG6	12	17.2	1.5
**2-oxoisovalerate dehydrogenase subunit beta, mitochondrial**	BCKDHB	P21953	16	20.4	1.5

**Serine/threonine-protein kinase tousled-like 2**	TLK2	Q86UE8	14	10.1	1.5
**Epsin-2**	EPN2	O95208	3	4.2	1.5
**Glycerol-3-phosphate dehydrogenase, mitochondrial**	GPD2	P43304	56	51.6	1.5
**ZW10 interactor**	ZWINT	O95229	2	5.1	1.5
**Growth factor receptor-bound protein 2**	GRB2	P62993	12	23.5	1.5
**Integrin alpha-3**	ITGA3	P26006	2	1.6	1.5
**Kinesin-like protein KIF14**	KIF14	Q15058	2	0.7	1.5
**Putative uncharacterized protein DKFZp686I0955**	DKFZp686 I0955	Q5HYC3	3	4.3	1.5
**Junction plakoglobin**	JUP	Q7L3S5	30	32.2	1.5
**Endoplasmic reticulum aminopeptidase 1**	ERAP1	Q9NZ08	24	20.5	1.5
**Ubiquitin-like protein 3**	UBL3	O95164	3	23.1	-1.5
**Nibrin**	NBN	O60934	4	5.7	-1.5
**Vesicle-associated membrane protein-associated protein A**	VAPA	Q9P0L0	45	56.6	-1.5
**Sorbitol dehydrogenase**	SORD	Q00796	22	36.7	-1.5
**Thioredoxin**	TXN	P10599	13	48.6	-1.5
**Putative uncharacterized protein LYRM7**	LYRM7	A8MPQ9	2	14.4	-1.5
**Estrogen receptor**	ESR1	P03372	6	9.1	-1.5
**Glucose-6-phosphate isomerase**	GPI	P06744	99	54.1	-1.5
**Krueppel-like factor 16**	KLF16	Q9BXK1	2	15.9	-1.5
**Copper-transporting ATPase 1**	ATP7A	Q04656	2	1.3	-1.5
**Neuroblast differentiation-associated protein AHNAK**	AHNAK	Q09666	341	40.7	-1.5
**RNA polymerase II-associated protein 1**	RPAP1	Q9BWH6	6	2.7	-1.5
**UPF0488 protein C8orf33**	C8orf33	Q9H7E9	4	14	-1.5
**Liprin-alpha-1**	PPFIA1	Q13136	10	8.2	-1.5
**Protein YIF1B**	YIF1B	Q5BJH7	13	24.8	-1.5
**Eukaryotic translation initiation factor 3 subunit J**	EIF3J	O75822	7	19.8	-1.5
**Homer protein homolog 1**	HOMER1	Q86YM7	2	5.1	-1.5
**Cullin-4B**	CUL4B	Q13620	29	18.6	-1.5
**Protein cordon-bleu**	COBL	O75128	3	1.4	-1.5
**Elongation factor G 2, mitochondrial**	GFM2	Q969S9	10	11.7	-1.5
**Protein kinase C iota type**	PRKCI	P41743	13	19.3	-1.5
**CD276 antigen**	CD276	Q5ZPR3	10	18.9	-1.5
**RNA polymerase-associated protein RTF1 homolog**	RTF1	Q92541	5	4.5	-1.5

**Ankyrin repeat domain-containing protein 46**	ANKRD46	Q86W74	2	11.6	-1.5
**APAF1-interacting protein**	APIP	Q96GX9	2	11.6	-1.5
**dUTP pyrophosphatase**	DUT	P33316	11	31.7	-1.5
**Brain protein 16**	BRP16	Q9BTY7	6	19	-1.5
**NF-kappa-B-repressing factor**	NKRF	O15226	12	14.6	-1.5
**Acyl-coenzyme A thioesterase 13**	ACOT13	Q9NPJ3	9	36.4	-1.5
**Estradiol 17-beta-dehydrogenase 8**	HSD17B8	Q92506	8	19.2	-1.5
**Telomerase Cajal body protein 1**	WDR79	Q9BUR4	2	4	-1.5
**5'-nucleotidase domain-containing protein 1**	NT5DC1	Q5TFE4	9	15.2	-1.5
**Calpain-2 catalytic subunit**	CAPN2	A6NDG7	4	4.3	-1.5
**Myoglobin**	MB	P02144	2	18.8	-1.5
**Alstrom syndrome protein 1**	ALMS1	Q8TCU4	2	0.6	-1.5
**Methyl-CpG-binding domain protein 2**	MBD2	Q9UBB5	3	15.6	-1.5
**Transmembrane emp24 domain-containing protein 4**	TMED4	Q7Z7H5	2	22.5	-1.5
**Nuclear factor of activated T-cells, cytoplasmic 2**	NFATC2	Q13469	2	7	-1.5
**Putative ATP-dependent RNA helicase DHX57**	DHX57	Q6P158	5	4.8	-1.5
**NAD(P)H dehydrogenase [quinone] 1**	NQO1	P15559	15	32.1	-1.5
**Conserved oligomeric Golgi complex subunit 6**	COG6	Q9Y2V7	2	8.7	-1.5
**Cancer susceptibility candidate gene 4 protein**	CASC4	Q6P4E1	2	6.2	-1.6
**Transmembrane channel-like protein 4**	TMC4	Q7Z404	10	2.7	-1.6
**Leucine-rich repeat-containing protein 8D**	LRRC8D	Q7L1W4	3	2.8	-1.6
**Methylcrotonoyl-CoA carboxylase beta chain, mitochondrial**	MCCC2	Q9HCC0	108	54.9	-1.6
**LRCH1 protein**	LRCH1	Q17R43	3	2.1	-1.6
**Patatin-like phospholipase domain-containing protein 2**	PNPLA2	Q96AD5	3	3.2	-1.6
**Gamma-enolase**	ENO2	P09104	14	32.7	-1.6
**Oxysterol-binding protein-related protein 1**	OSBPL1A	Q9BXW6	6	5.2	-1.6
**DNA-binding protein A**	CSDA	P16989	6	28.5	-1.6
**Neurolysin, mitochondrial**	NLN	Q9BYT8	22	24.1	-1.6

**AFG3-like protein 2**	AFG3L2	Q9Y4W6	20	22.7	-1.6
**Deoxyribonuclease-1-like 1**	DNASE1L 1	P49184	2	8.9	-1.6
**40S ribosomal protein S27**	RPS27	P42677	2	39.3	-1.6
**Forkhead box protein J3**	FOXJ3	Q9UPW0	4	5.8	-1.6
**Putative uncharacterized protein DKFZp686P03159**	DKFZp686 P03159	Q6N0B3	74	61.3	-1.6
**Hydroxysteroid dehydrogenase-like protein 2**	HSDL2	Q6YN16	8	18.2	-1.6
**DNA replication complex GINS protein PSF3**	GINS3	Q9BRX5	2	7.4	-1.6
**Chromosome 10 open reading frame 35**	C10orf35	Q5VVH9	2	8	-1.6
**PHD finger protein 10**	PHF10	Q8WUB8	6	9	-1.6
**Eukaryotic translation initiation factor 5B**	EIF5B	O60841	26	16.3	-1.6
**Baculoviral IAP repeat-containing protein 4**	XIAP	P98170	2	3.6	-1.6
**Trafficking protein particle complex subunit 6A**	TRAPPC6 A	O75865	4	24.3	-1.6
**Putative ribosome-binding factor A, mitochondrial**	C18orf22	Q8N0V3	2	5.8	-1.6
**Multiple ankyrin repeats single KH domain protein isoform 2**	hCG_204 5902	Q8IWZ2	8	5.1	-1.6
**La-related protein 2**	LARP2	Q659C4	2	4.3	-1.6
**Integrin beta-1**	ITGB1	Q8WUM6	19	11.3	-1.6
**UPF0672 protein C3orf58**	C3orf58	Q8NDZ4	3	4	-1.6
**Serine/threonine-protein kinase 3**	STK3	Q13188	4	5.1	-1.6
**Annexin A1**	ANXA1	P04083	2	6.9	-1.6
**Transferrin receptor protein 1**	TFRC	P02786	33	19.9	-1.6
**Zinc finger protein 451**	ZNF451	Q9Y4E5	3	2.9	-1.6
**CD63 antigen**	CD63	P08962	3	4.2	-1.6
**Stromal cell-derived factor 2-like protein 1**	SDF2L1	Q9HCN8	18	23.3	-1.6
**Anterior gradient protein 2 homolog**	AGR2	O95994	65	47.7	-1.6
**Transcriptional activator protein Pur-alpha**	PURA	Q00577	23	42.5	-1.6
**Occludin**	OCLN	Q16625	3	7.7	-1.6
**Polypyrimidine tract-binding protein 2**	PTBP2	Q9UKA9	17	27	-1.6
			2	87.1	-1.6
**NEDD8-conjugating enzyme Ubc12**	UBE2M	P61081	7	21.3	-1.6

**Heat shock 70 kDa protein 12A**	HSPA12A	O43301	7	14.2	-1.6
			4	81.4	-1.6
**Kinesin-like protein KIF2A**	KIF2A	O00139	5	6.2	-1.7
**Creatine kinase, ubiquitous mitochondrial**	CKMT1A	P12532	37	39.6	-1.7
**SLIT-ROBO Rho GTPase-activating protein 1**	SRGAP1	Q7Z6B7	8	5.8	-1.7
**Cell division protein kinase 5**	CDK5	Q00535	11	24.3	-1.7
**Partitioning defective 6 homolog beta**	PARD6B	Q9BYG5	10	22.8	-1.7
**Synembryn-A**	RIC8A	Q9NPQ8	7	13	-1.7
**Uncharacterized protein C16orf89**	C16orf89	Q6UX73	2	1.6	-1.7
**Rho guanine nucleotide exchange factor 2**	ARHGEF2	Q9H023	8	9.4	-1.7
**Interferon-induced transmembrane protein 2**	IFITM2	Q01629	2	29.5	-1.7
**Moesin-ezrin-radixin-like protein**	NF2	P35240	3	2.7	-1.7
**Procollagen-lysine,2-oxoglutarate 5-dioxygenase 2**	PLOD2	O00469	7	6.3	-1.7
**Interferon-induced transmembrane protein 1**	IFITM1	P13164	2	27.2	-1.7
**Fatty acyl-CoA reductase 1**	FAR1	Q8WVX9	14	17.3	-1.7
**Armadillo repeat protein deleted in velo-cardio-facial syndrome**	ARVCF	O00192	3	3.3	-1.7
**Mitochondrial import inner membrane translocase subunit Tim8 A**	TIMM8A	O60220	6	22.7	-1.7
**Zinc finger CCCH-type antiviral protein 1-like**	ZC3HAV1L	Q96H79	6	11	-1.7
**Protein LAP2**	ERBB2IP	Q96RT1	8	8.6	-1.7
**DNA-binding protein SMUBP-2**	IGHMBP2	P38935	2	5	-1.7
**Putative uncharacterized protein EIF4E**	EIF4E	A8MX72	3	13.4	-1.7
**Tubulin folding cofactor B**	TBCB	Q99426	3	14.3	-1.7
**N(G)-dimethylarginine dimethylaminohydrolase 1**	DDAH1	O94760	18	37.5	-1.7
**26S proteasome non-ATPase regulatory subunit 10**	PSMD10	O75832	6	22.1	-1.7
**Methionine aminopeptidase 2**	METAP2	P50579	2	2.1	-1.7

**E3 ubiquitin-protein ligase ZNRF1**	ZNRF1	Q8ND25	2	5	-1.7
**Putative uncharacterized protein CD81**	CD81	A6NMH8	20	14.2	-1.7
**DNAJC10 protein**	DNAJC10	Q3B7W8	9	4.3	-1.7
**Kynurenine--oxoglutarate transaminase 1**	CCBL1	Q16773	2	10.2	-1.7
**Amidophosphoribosyltransferase**	PPAT	Q06203	16	23	-1.7
**Sortilin-related receptor**	SORL1	Q92673	3	1.9	-1.7
**Putative uncharacterized protein NACA**	NACA	A8MTN7	12	5.9	-1.7
**Dihydropyrimidinase-related protein 2**	DPYSL2	Q16555	6	19.2	-1.7
**MAGUK p55 subfamily member 5**	MPP5	Q8N3R9	2	3.9	-1.8
**Cadherin EGF LAG seven-pass G-type receptor 2**	CELSR2	Q9HCU4	6	2.2	-1.8
**InaD-like protein**	INADL	Q8NI35	10	3.7	-1.8
			6	16	-1.8
**Glutaminyl-peptide cyclotransferase-like protein**	QPCTL	Q9NXS2	2	8.4	-1.8
**Isochorismatase domain-containing protein 2, mitochondrial**	ISOC2	Q96AB3	36	60.2	-1.8
**Small EDRK-rich factor 2**	SERF2	P84101	6	16.9	-1.8
**Kelch domain-containing protein 7B**	KLHDC7B	Q96G42	2	6.9	-1.8
**Cathepsin D**	CTSD	P07339	85	30.1	-1.8
**Prostate tumor overexpressed gene 1 protein**	PTOV1	Q86YD1	2	6.2	-1.8
**Histone-lysine N-methyltransferase SETD7**	SETD7	Q8WTS6	2	7.1	-1.8
**Lysophosphatidylcholine acyltransferase 1**	LPCAT1	Q8NF37	3	3.4	-1.8
**Transcription intermediary factor 1-alpha**	TRIM24	O15164	14	14.7	-1.8
**Thymidylate synthase**	TYMS	P04818	3	11.2	-1.8
**EH domain-containing protein 4**	EHD4	Q9H223	13	23.3	-1.8
**Ankyrin repeat domain-containing protein 27**	ANKRD27	Q96NW4	8	8.4	-1.8
**NF-kappa-B-activating protein**	NKAP	Q8N5F7	6	18.3	-1.8
**Microtubule-associated protein tau**	MAPT	P10636	11	20	-1.8
**Heat shock protein beta-11**	HSPB11	Q9Y547	2	8.3	-1.9

**Bleomycin hydrolase**	BLMH	Q13867	4	9.5	-1.9
**Microtubule-actin cross-linking factor 1, isoforms 1/2/3/5**	MACF1	Q9UPN3	18	4.4	-1.9
**Erythrocyte band 7 integral membrane protein**	STOM	P27105	6	17	-1.9
**Membrane-associated guanylate kinase, WW and PDZ domain-containing protein 3**	MAGI3	Q5TCR0	2	2.3	-1.9
**Solute carrier family 2, facilitated glucose transporter member 1**	SLC2A1	P11166	3	3.9	-1.9
**Ferrochelatase**	FECH	Q8NAN0	17	25.4	-1.9
**UPF0577 protein KIAA1324**	KIAA1324	Q6UXG2	7	8.6	-1.9
**Endothelial protein C receptor**	PROCR	Q9UNN8	3	6.2	-1.9
**CD166 antigen**	ALCAM	Q13740	30	38.6	-1.9
**ADP-ribosylation factor GTPase-activating protein 3**	ARFGAP3	Q9NP61	2	6.6	-1.9
**Twinfilin-1**	TWF1	Q12792	5	15.9	-1.9
**Proto-oncogene tyrosine-protein kinase Yes**	YES1	P07947	6	11.4	-1.9
**Phosphatidylserine synthase 1**	PTDSS1	P48651	2	2.7	-1.9
**Prolyl 4-hydroxylase subunit alpha-2**	P4HA2	O15460	3	3.6	-1.9
**Elongation of very long chain fatty acids protein 5**	ELOVL5	Q9NYP7	11	16.4	-1.9
**Protein disulfide-isomerase A3**	PDIA3	P30101	102	54.7	-1.9
**Forkhead box protein O3**	FOXO3	O43524	2	6.5	-1.9
**Filamin-B**	FLNB	O75369	371	51.3	-1.9
**Cytochrome P450 4F22**	CYP4F22	Q6NT55	9	11.9	-1.9
**Putative uncharacterized protein DKFZp686J1254**	DKFZp686 J1254	Q68CT2	2	9.3	-1.9
**Transcription elongation factor A protein-like 4**	TCEAL4	Q96EI5	4	7	-2.0
**BTB/POZ domain-containing protein KCTD3**	KCTD3	Q9Y597	3	7.6	-2.0
**RalBP1-associated Eps domain-containing protein 2**	REPS2	Q8NFH8	3	4.1	-2.0
**PR domain zinc finger protein 2**	PRDM2	Q13029	2	1.5	-2.0
**Palladin**	PALLD	Q8WX93	15	6.9	-2.0
**Cysteine-rich protein 2**	CRIP2	P52943	8	34.1	-2.0
**Four and a half LIM-domain protein 2**	FHL2	Q2I5I4	3	5.7	-2.0

**Cellular retinoic acid-binding protein 1**	CRABP1	P29762	6	38.7	-2.0
**4F2 cell-surface antigen heavy chain**	SLC3A2	P08195	64	32.1	-2.0
**Hexokinase-2**	HK2	P52789	2	3.8	-2.0
**Gem-associated protein 7**	GEMIN7	Q9H840	2	35.1	-2.0
**COBW domain-containing protein 2**	CBWD2	Q8IUF1	2	17.2	-2.0
**Placenta derived apoptotic factor**	PDAF	Q5Y8C7	2	16.7	-2.0
**Leukocyte surface antigen CD47**	CD47	Q08722	3	4.4	-2.0
**Myoferlin**	MYOF	Q9NZM1	34	14.3	-2.0
**Glutamine synthetase**	GLUL	P15104	6	14.7	-2.0
**UPF0364 protein C6orf211**	C6orf211	Q9H993	33	33.1	-2.1
**UPF0534 protein C4orf43**	C4orf43	Q96EY4	9	21.2	-2.1
**KIAA1688 protein**	KIAA1688	Q6PJQ0	3	3.2	-2.1
**Dystonin**	DST	Q5TBT2	7	1.2	-2.1
**Prolyl 4-hydroxylase subunit alpha-1**	P4HA1	P13674	8	15.4	-2.2
**Protein GREB1**	GREB1	Q4ZG55	3	4.8	-2.2
**Adenylate kinase isoenzyme 6**	AK6	Q9Y3D8	7	9.3	-2.2
**Beta-soluble NSF attachment protein**	NAPB	Q9H115	2	5	-2.2
**Prolyl 3-hydroxylase 1**	LEPRE1	Q32P28	10	2.7	-2.2
**Ceramide glucosyltransferase**	UGCG	Q16739	2	7.4	-2.3
**Spectrin beta chain, brain 1**	SPTBN1	Q01082	4	21.3	-2.3
**Protein CDV3 homolog**	CDV3	Q9UKY7	28	63.2	-2.3
**ATP-binding cassette sub-family D member 3**	ABCD3	P28288	18	19.3	-2.3
**Transcription elongation factor A protein-like 3**	TCEAL3	Q969E4	3	16.5	-2.4
**Very long-chain acyl-CoA synthetase**	SLC27A2	O14975	19	17.4	-2.4
**RNA-binding protein 38**	RBM38	Q9H0Z9	3	15.1	-2.4
**Secretory carrier-associated membrane protein 3**	SCAMP3	B1AVS6	7	18.4	-2.4
**Integrin alpha-2**	ITGA2	P17301	30	18.9	-2.4
**Isochorismatase domain-containing protein 1**	ISOC1	Q96CN7	33	53	-2.4
**Tumor protein D53**	TPD52L1	Q16890	12	35.3	-2.4
**Sulfotransferase 1A1**	SULT1A1	P50225	12	24.9	-2.4
**Putative uncharacterized protein ANXA3**	ANXA3	A6NLK4	3	13.3	-2.4
**Tubulin beta-6 chain**	TUBB6	Q9BUF5	17	48.8	-2.4
**Methyltransferase-like protein 7A**	METTL7A	Q9H8H3	4	13.5	-2.4

**Peroxisomal Lon protease homolog 2**	LONP2	Q86WA8	10	12.2	-2.4
**Secretory carrier-associated membrane protein 1**	SCAMP1	O15126	12	20.4	-2.4
**5'-nucleotidase domain-containing protein 3**	NT5DC3	Q86UY8	9	14.1	-2.5
**Vesicle transport protein GOT1B**	GOLT1B	Q9Y3E0	2	10.1	-2.5
**Cytoplasmic FMR1-interacting protein 2**	CYFIP2	Q96F07	2	9.7	-2.5
**Ribonucleoside-diphosphate reductase subunit M2**	RRM2	P31350	11	13.1	-2.5
**Rhophilin-2**	RHPN2	Q8IUC4	6	5.8	-2.5
**Semaphorin-3C**	SEMA3C	Q99985	4	7.8	-2.5
**Spartin**	SPG20	Q8N0X7	2	4.8	-2.5
**Codanin-1**	CDAN1	Q8IWY9	2	3.9	-2.5
**Aflatoxin B1 aldehyde reductase member 3**	AKR7A3	O95154	2	7.9	-2.5
**Tubulin beta-4 chain**	TUBB4	P04350	5	62.2	-2.6
**Plasma membrane calcium-transporting ATPase 4**	ATP2B4	P23634	4	7.7	-2.6
**Acyl-CoA desaturase**	SCD	O00767	7	17.8	-2.6
**Cytochrome P450 4F11**	CYP4F11	Q9HBI6	2	2.7	-2.6
**Large neutral amino acids transporter small subunit 1**	SLC7A5	Q01650	3	6.9	-2.6
**Pannexin-1**	PANX1	Q96RD7	2	3.3	-2.6
**Endothelin-converting enzyme 1**	ECE1	P42892	5	7.7	-2.6
**Inositol monophosphatase 2**	IMPA2	O14732	2	8	-2.6
**Glutaredoxin-1**	GLRX	P35754	4	27.4	-2.6
**Interferon alpha-inducible protein 27**	IFI27	P40305	3	41.8	-2.6
**DEP domain-containing mTOR-interacting protein**	DEPDC6	Q8TB45	2	8.8	-2.7
**Pleckstrin homology-like domain family B member 1**	PHLDB1	Q86UU1	13	9	-2.7
**Endonuclease domain-containing 1 protein**	ENDOD1	O94919	3	11.2	-2.7
**Protein ETHE1, mitochondrial**	ETHE1	O95571	16	47.2	-2.8
**Trophoblast glycoprotein**	TPBG	Q13641	14	13.6	-2.8
**ADP-ribosylation factor-like protein 3**	ARL3	P36405	17	61.5	-2.8
**Prostaglandin reductase 1**	PTGR1	Q14914	20	38	-2.8
**Protein S100-A14**	S100A14	Q9HCY8	4	26	-2.9
**Carbonic anhydrase 12**	CA12	O43570	12	20.6	-2.9
**Sideroflexin-2**	SFXN2	Q96NB2	8	22	-3.0

**Neutral amino acid transporter A**	SLC1A4	P43007	5	17.1	-3.1
**Sialidase-3**	NEU3	Q9UQ49	2	9.3	-3.1
**Dipeptidyl-peptidase 1**	CTSC	P53634	3	2.6	-3.1
**Molybdenum cofactor synthesis protein 2A**	MOCS2	O96033	4	18.2	-3.2
**Annexin A6**	ANXA6	P08133	100	54.4	-3.2
**Zinc transporter ZIP6**	SLC39A6	Q13433	4	1.3	-3.3
**CDC42 small effector protein 2**	CDC42SE2	Q4KMT9	6	66.7	-3.3
**Serum response factor**	SRF	P11831	4	9.6	-3.5
**Cyclic AMP-dependent transcription factor ATF-7**	ATF7	P17544	2	16.8	-3.5
**Band 4.1-like protein 1**	EPB41L1	Q9H4G0	7	9.1	-3.5
**Molybdenum cofactor synthesis protein 2B**	MOCS2	O96007	4	25	-3.5
**Talin-2**	TLN2	Q9Y4G6	5	8.1	-3.8
**Caspase-14**	CASP14	P31944	2	5.8	-3.9
**Insulin receptor substrate 1**	IRS1	P35568	7	7.7	-3.9
**Insulin-like growth factor 1 receptor**	IGF1R	P08069	4	3.4	-4.0
**Adseverin**	SCIN	Q9Y6U3	5	9	-4.7
**Carbonic anhydrase-related protein**	CA8	P35219	3	12.8	-4.9
**Argininosuccinate synthase**	ASS1	P00966	39	39.8	-5.1
**PDZ and LIM domain protein 3**	PDLIM3	Q53GG5	3	9.2	-5.3
**Protein phosphatase 1E**	PPM1E	Q8WY54	3	4	-5.5
**Pre-mRNA cleavage complex 2 protein Pcf11**	PCF11	O94913	11	4.7	-5.9
**Immunoglobulin superfamily member 1**	IGSF1	Q8N6C5	4	2.8	-5.9
**Amine oxidase [flavin-containing] B**	MAOB	P27338	2	2.9	-6.1
**Carbamoylphosphate synthetase I**	CPS1	Q5R206	2	2.8	-6.2
**Sodium-driven chloride bicarbonate exchanger**	SLC4A10	Q6U841	2	2.6	-6.7
**Protein NDRG1**	NDRG1	Q92597	18	32	-6.8
**Na(+)/H(+) exchange regulatory cofactor NHE-RF3**	PDZK1	Q5T2W1	8	12.3	-7.2
**Apoptosis regulator Bcl-2**	BCL2	P10415	7	12.6	-7.5
**Adenylyl cyclase-associated protein 2**	CAP2	P40123	7	20.1	-9.0
**Protein S100-A6**	S100A6	P06703	2	26.7	-9.6
**Progesterone receptor**	PGR	P06401	9	8.5	-10.1
**Neural cell adhesion molecule 2**	NCAM2	O15394	3	6.7	-11.4

**Signal peptide, CUB and EGF-like domain-containing protein 2**	SCUBE2	Q9NQ36	3	1.4	-12.4
**cDNA FLJ75801**	RBBP8	A8K8W6	2	2	-13.3
**Collagen alpha-1(XII) chain**	COL12A1	Q99715	31	12.9	-19.0
**Ras-related protein Rab-31**	RAB31	Q13636	4	27.2	-20.5
**Matrix Gla protein**	MGP	P08493	8	32	-25.1
**Aldo-keto reductase family 1 member C1**	AKR1C1	Q04828	18	27.9	-33.3
**Hemoglobin subunit alpha**	HBA1	P69905	5	23.9	-44.9
**Syntabulin**	SYBU	Q9NX95	3	7.8	-97.8

Supplementary Table 2. Clinical characteristic of the three cohorts of postmenopausal breast cancer patients used in the immunohistochemical studies

	Cohort 1	Cohort 2		Cohort 4		
	All patients n (%)			All patients n (%)	MCM3 ≤10% n (%)	MCM3 ≥10% n (%)
**Parameter**						
**Patients**	79 (100)	218 (100)		683 (100)	335(49)	348 (51)
**Age (Years)**						
Median (range)	61 (48-74)	61 (48-74)				
<59	33 (42)	100 (46)	≤60	221 (32)	102 (30)	119 (34)
60-69	33 (42)	88 (40)	>60	462 (68)	233 (70)	229 (66)
>69	13 (16)	30 (14)				
**Tumor size (mm)**						
≤ 20	34 (43)	89 (41)		506 (76)	252 (77)	254 (75)
> 20	43 (54)	129 (59)		161 (24)	76 (23)	85 (25)
Unknown	2 (3)			16 (2)	7 (2)	9 (3)
**Estrogen receptor status (retrospective)**						
ER+	79 (≥ 10%) (100)	218 (≥ 1%) (100)		516 (≥ 10%) (76)	279 (83)	237 (68)
ER-				147 (22)	47 (14)	100 (29)
Unknown				20 (3)	9 (3)	11 (3)
**Lymph nodes (tumor positive)**			**HER2 status (retrospective)**			
0	6 (8)	17 (8)	HER2-	556 (81)	282 (84)	274 (79)
1-3	43 (54)	122 (56)	HER2+	75 (11)	22 (7)	53 (15)
≥ 4	30 (38)	79 (36)	Unknown	52 (8)	31 (9)	21 (6)
**Tumor grade (ductal)**						
1	20 (25)	48 (22)		100 (15)	69 (21)	31 (9)
2	44 (56)	85 (39)		346 (51)	179 (53)	167 (48)
3	15 (19)	53 (24)		145 (21)	36 (11)	109 (31)
Unknown	15 (19)	32 (15)		92 (13)	51 (15)	41 (12)
**Recurrence (<10 years)**			**Recurrence**			
No	35 (44)	150 (69)	No	506 (74)		
Yes	44 (56)	68 (31)	Yes	177 (26)		
**Death (< 10 years)**			**Tamoxifen**			
No	43 (54)	131 (60)	No	339 (49)	166 (50)	168 (48)
Yes	36 (46)	87 (40)	Yes	349 (51)	169 (50)	180 (52)

Supplementary Table 3. Alteration of MCM3 Axis in Breast Cancer Cells during Adaption to Grow in Estrogen Deprived Medium.

Cell line	Control vs.LTED-cells	MCM3 level (fold-change)	Accession no.	Sample no.
MCF7	0 vs. 3 days	-1.1	GSE20361	GSM510103<br>GSM510104<br>GSM510105<br>GSM510106<br>GSM510107<br>GSM510108<br>GSM510109<br>GSM510110
	0 vs. 15 days	-1.44		
	0 vs. 30 days	-1.57		
	0 vs. 90 days	1.19		
	0 vs. 120 days	1.43		
	0 vs. 150 days	1.69		
	0 vs. 180 days	1.69		
MCF7	Control vs. MCF7/LTED	1.7	GSE38829	GSM950396<br>GSM950398
MCF7	0 vs. 2 days	1	GSE50820	GSM1230317<br>GSM1230344<br>GSM1230345<br>GSM1230346
	0 vs. 6 weeks	-1.6		
	0 vs. 10 months	1.5		

Three microarray gene datasets were downloaded from the GEO database and analysed in Partek Genomic Suite. The MCM3 level were studied in control cells and compared with cells at several time points to follow the alteration of the MCM3 axis during adaption to grow in estrogen-deprived medium.

Supplemantary Table 4. Genes exhibiting altered expression in TamR cells following MCM3 knockdown (transfected with MCM3-specific siRNAs) versus cells transfected with siControl (FDR < 0.05 and ≥ 1.5 fold altered expression).

Column #	Probeset ID	Entrez Gene	Gene Symbol	Gene Title	Fold-Change(TamR1kd vs. TamR1ct)		Fold-Change(TamR4kd vs. TamR4		Fold-Change(TamR7kd vs. TamR7ct)	
45729	236472_at	---	---	---	1,90547	TamR1kd up vs TamR1ct	-1,72157	TamR4kd down vs Tar	1,56184	TamR7kd up vs TamR7ct
46692	237435_at	---	---	---	2,88213	TamR1kd up vs TamR1ct	1,80237	TamR4kd up vs TamR	2,02797	TamR7kd up vs TamR7ct
35627	226363_at	10057	ABCC5	ATP-binding cassette, su	1,60265	TamR1kd up vs TamR1ct	1,54129	TamR4kd up vs TamR	1,60057	TamR7kd up vs TamR7ct
54461	49452_at	32	ACACB	acetyl-CoA carboxylase	1,70048	TamR1kd up vs TamR1ct	1,66255	TamR4kd up vs TamR	1,50883	TamR7kd up vs TamR7ct
18582	209160_at	8644	AKR1C3	aldo-keto reductase fam	2,04086	TamR1kd up vs TamR1ct	1,61216	TamR4kd up vs TamR	2,47685	TamR7kd up vs TamR7ct
14475	205020_s_at	10124	ARL4A	ADP-ribosylation factor	1,7172	TamR1kd up vs TamR1ct	1,58197	TamR4kd up vs TamR	1,85577	TamR7kd up vs TamR7ct
14888	205433_at	590	BCHE	butyrylcholinesterase	2,33665	TamR1kd up vs TamR1ct	2,81976	TamR4kd up vs TamR	1,66928	TamR7kd up vs TamR7ct
17104	207655_s_at	29760	BLNK	B-cell linker	2,69024	TamR1kd up vs TamR1ct	1,67855	TamR4kd up vs TamR	2,15359	TamR7kd up vs TamR7ct
35240	225976_at	91408	BTF3L4	basic transcription facto	-1,61032	TamR1kd down vs TamR1ct	-1,77264	TamR4kd down vs Tar	-1,65454	TamR7kd down vs TamR7ct
18453	209031_at	23705	CADM1	cell adhesion molecule	2,06099	TamR1kd up vs TamR1ct	1,70165	TamR4kd up vs TamR	1,78255	TamR7kd up vs TamR7ct
19810	210404_x_at	816	CAMK2B	calcium/calmodulin-de	1,51348	TamR1kd up vs TamR1ct	-2,20387	TamR4kd down vs Tar	-1,63512	TamR7kd down vs TamR7ct
19796	210390_s_at	6358 /// 6359 /,	CCL14 /// CCL1	chemokine (C-C motif) l	2,02951	TamR1kd up vs TamR1ct	2,00914	TamR4kd up vs TamR	1,61952	TamR7kd up vs TamR7ct
16	1405_i_at	6352	CCL5	chemokine (C-C motif) l	1,85822	TamR1kd up vs TamR1ct	3,75606	TamR4kd up vs TamR	2,30635	TamR7kd up vs TamR7ct
14110	204655_at	6352	CCL5	chemokine (C-C motif) l	2,16823	TamR1kd up vs TamR1ct	3,32458	TamR4kd up vs TamR	2,16137	TamR7kd up vs TamR7ct
45570	236313_at	1030	CDKN2B	cyclin-dependent kinas	3,59582	TamR1kd up vs TamR1ct	2,10081	TamR4kd up vs TamR	2,26993	TamR7kd up vs TamR7ct
31836	222549_at	9076	CLDN1	claudin 1	2,74422	TamR1kd up vs TamR1ct	1,82797	TamR4kd up vs TamR	1,71196	TamR7kd up vs TamR7ct
30986	221698_s_at	64581	CLEC7A	C-type lectin domain fa	1,71023	TamR1kd up vs TamR1ct	2,86613	TamR4kd up vs TamR	1,87706	TamR7kd up vs TamR7ct
2556	1555756_a_at	64581	CLEC7A	C-type lectin domain fa	1,71835	TamR1kd up vs TamR1ct	4,00837	TamR4kd up vs TamR	1,72022	TamR7kd up vs TamR7ct
31331	222043_at	1191	CLU	clusterin	1,55553	TamR1kd up vs TamR1ct	3,09283	TamR4kd up vs TamR	1,52621	TamR7kd up vs TamR7ct
18214	208791_at	1191	CLU	clusterin	1,61607	TamR1kd up vs TamR1ct	2,21742	TamR4kd up vs TamR	1,85368	TamR7kd up vs TamR7ct
18215	208792_s_at	1191	CLU	clusterin	1,72165	TamR1kd up vs TamR1ct	2,38516	TamR4kd up vs TamR	1,88213	TamR7kd up vs TamR7ct
37631	228369_at	10695	CNPY3	canopy 3 homolog (zeb	2,15953	TamR1kd up vs TamR1ct	2,80391	TamR4kd up vs TamR	2,19441	TamR7kd up vs TamR7ct
17251	207802_at	10321	CRISP3	cysteine-rich secretory	1,97772	TamR1kd up vs TamR1ct	1,75101	TamR4kd up vs TamR	4,00574	TamR7kd up vs TamR7ct
14426	204971_at	1475	CSTA	cystatin A (stefin A)	2,90071	TamR1kd up vs TamR1ct	3,7962	TamR4kd up vs TamR	3,31914	TamR7kd up vs TamR7ct
19508	210096_at	1580	CYP4B1	cytochrome P450, fami	1,7164	TamR1kd up vs TamR1ct	1,75189	TamR4kd up vs TamR	1,73719	TamR7kd up vs TamR7ct
14854	205399_at	9201	DCLK1	doublecortin-like kinas	1,51298	TamR1kd up vs TamR1ct	1,52733	TamR4kd up vs TamR	1,51154	TamR7kd up vs TamR7ct
32080	222793_at	23586	DDX58	DEAD (Asp-Glu-Ala-Asp)	1,79063	TamR1kd up vs TamR1ct	1,61042	TamR4kd up vs TamR	2,1112	TamR7kd up vs TamR7ct
28278	218986_s_at	55601	DDX60	DEAD (Asp-Glu-Ala-Asp)	2,22817	TamR1kd up vs TamR1ct	1,62532	TamR4kd up vs TamR	2,5954	TamR7kd up vs TamR7ct
37414	228152_s_at	91351	DDX60L	DEAD (Asp-Glu-Ala-Asp)	1,951	TamR1kd up vs TamR1ct	1,66184	TamR4kd up vs TamR	2,19699	TamR7kd up vs TamR7ct
42104	232843_s_at	81704	DOCK8	dedicator of cytokinesis	1,72039	TamR1kd up vs TamR1ct	1,74917	TamR4kd up vs TamR	1,82846	TamR7kd up vs TamR7ct
34767	225502_at	81704	DOCK8	dedicator of cytokinesis	2,19335	TamR1kd up vs TamR1ct	2,0096	TamR4kd up vs TamR	2,08082	TamR7kd up vs TamR7ct
14101	204646_at	1806	DPYD	dihydropyrimidine deh	1,54499	TamR1kd up vs TamR1ct	1,70804	TamR4kd up vs TamR	1,99724	TamR7kd up vs TamR7ct
15569	206115_at	1960	EGR3	early growth response 3	-1,9543	TamR1kd down vs TamR1ct	-1,52805	TamR4kd down vs Tar	-1,63237	TamR7kd down vs TamR7ct
17140	207691_x_at	953	ENTPD1	ectonucleoside triphos	1,56847	TamR1kd up vs TamR1ct	2,11409	TamR4kd up vs TamR	1,85005	TamR7kd up vs TamR7ct
18895	209474_s_at	953	ENTPD1	ectonucleoside triphos	1,66758	TamR1kd up vs TamR1ct	1,59731	TamR4kd up vs TamR	2,84487	TamR7kd up vs TamR7ct
31641	222354_at	50848	F11R	F11 receptor	-2,28643	TamR1kd down vs TamR1ct	-1,78496	TamR4kd down vs Tar	-1,67164	TamR7kd down vs TamR7ct
32345	223058_at	83641	FAM107B	family with sequence si	1,63261	TamR1kd up vs TamR1ct	1,63369	TamR4kd up vs TamR	1,82972	TamR7kd up vs TamR7ct
33600	224325_at	8325	FZD8	frizzled family receptor	-1,87585	TamR1kd down vs TamR1ct	-1,5867	TamR4kd down vs Tar	-1,72351	TamR7kd down vs TamR7ct
40839	231577_s_at	2633	GBP1	guanylate binding prote	1,60708	TamR1kd up vs TamR1ct	1,59986	TamR4kd up vs TamR	2,06639	TamR7kd up vs TamR7ct
21757	212444_at	9052	GPRC5A	G protein-coupled rece	1,63515	TamR1kd up vs TamR1ct	1,6649	TamR4kd up vs TamR	1,98047	TamR7kd up vs TamR7ct
12565	203108_at	9052	GPRC5A	G protein-coupled rece	1,71108	TamR1kd up vs TamR1ct	2,29223	TamR4kd up vs TamR	2,11239	TamR7kd up vs TamR7ct
13604	204149_s_at	2948	GSTM4	glutathione S-transfera	1,82146	TamR1kd up vs TamR1ct	1,72242	TamR4kd up vs TamR	1,86628	TamR7kd up vs TamR7ct
13690	204235_s_at	51454	GULP1	GULP, engulfment adap	1,59675	TamR1kd up vs TamR1ct	2,12105	TamR4kd up vs TamR	1,71054	TamR7kd up vs TamR7ct
13692	204237_at	51454	GULP1	GULP, engulfment adap	1,73608	TamR1kd up vs TamR1ct	1,78843	TamR4kd up vs TamR	1,86906	TamR7kd up vs TamR7ct
25214	215913_s_at	51454	GULP1	GULP, engulfment adap	2,70308	TamR1kd up vs TamR1ct	1,59559	TamR4kd up vs TamR	2,10557	TamR7kd up vs TamR7ct
28644	219352_at	55008	HERC6	HECT and RLD domain c	1,95659	TamR1kd up vs TamR1ct	3,36203	TamR4kd up vs TamR	2,41648	TamR7kd up vs TamR7ct
13870	204415_at	2537	IFI6	interferon, alpha-induc	2,61085	TamR1kd up vs TamR1ct	2,03091	TamR4kd up vs TamR	2,13886	TamR7kd up vs TamR7ct
12609	203153_at	3434	IFIT1	interferon-induced prot	2,81582	TamR1kd up vs TamR1ct	2,70187	TamR4kd up vs TamR	3,41063	TamR7kd up vs TamR7ct
36020	226757_at	3433	IFIT2	interferon-induced prot	1,89583	TamR1kd up vs TamR1ct	1,73741	TamR4kd up vs TamR	2,88227	TamR7kd up vs TamR7ct
38712	229450_at	3437	IFIT3	interferon-induced prot	2,2467	TamR1kd up vs TamR1ct	1,76802	TamR4kd up vs TamR	3,12116	TamR7kd up vs TamR7ct
23329	214022_s_at	8519	IFITM1	interferon induced tran	1,96416	TamR1kd up vs TamR1ct	2,97349	TamR4kd up vs TamR	2,40492	TamR7kd up vs TamR7ct
11057	201601_x_at	8519 /// 10581	IFITM1 /// IFITN	interferon induced tran	1,66269	TamR1kd up vs TamR1ct	2,64375	TamR4kd up vs TamR	2,1741	TamR7kd up vs TamR7ct
13337	203882_at	10379	IRF9	interferon regulatory fa	2,61683	TamR1kd up vs TamR1ct	1,83485	TamR4kd up vs TamR	1,7562	TamR7kd up vs TamR7ct
14938	205483_s_at	9636	ISG15	ISG15 ubiquitin-like mo	1,71445	TamR1kd up vs TamR1ct	2,79928	TamR4kd up vs TamR	2,18601	TamR7kd up vs TamR7ct
19669	210261_at	3776	KCNK2	potassium channel, sub	2,21605	TamR1kd up vs TamR1ct	2,26505	TamR4kd up vs TamR	2,21492	TamR7kd up vs TamR7ct
14001	204546_at	9764	KIAA0513	KIAA0513	1,56107	TamR1kd up vs TamR1ct	1,59727	TamR4kd up vs TamR	1,7873	TamR7kd up vs TamR7ct
3483	1557165_s_at	23276	KLHL18	kelch-like family membe	1,64721	TamR1kd up vs TamR1ct	1,90607	TamR4kd up vs TamR	1,73488	TamR7kd up vs TamR7ct
15024	205569_at	27074	LAMP3	lysosomal-associated m	1,84931	TamR1kd up vs TamR1ct	2,67828	TamR4kd up vs TamR	1,9325	TamR7kd up vs TamR7ct
12455	202998_s_at	4017	LOXL2	lysyl oxidase-like 2	1,91297	TamR1kd up vs TamR1ct	1,64899	TamR4kd up vs TamR	1,66779	TamR7kd up vs TamR7ct
40125	230863_at	4036	LRP2	low density lipoprotein	1,74465	TamR1kd up vs TamR1ct	3,09403	TamR4kd up vs TamR	3,74437	TamR7kd up vs TamR7ct
15165	205710_at	4036	LRP2	low density lipoprotein	2,11673	TamR1kd up vs TamR1ct	2,86339	TamR4kd up vs TamR	2,97856	TamR7kd up vs TamR7ct
11011	201555_at	4172	MCM3	minichromosome main	-3,26685	TamR1kd down vs TamR1ct	-2,61897	TamR4kd down vs Tar	-3,36795	TamR7kd down vs TamR7ct
23213	213906_at	4603	MYBL1	v-myb myeloblastosis vi	-1,7497	TamR1kd down vs TamR1ct	-2,02417	TamR4kd down vs Tar	-1,61171	TamR7kd down vs TamR7ct
17765	208334_at	64579	NDST4	N-deacetylase/N-sulfotr	4,2186	TamR1kd up vs TamR1ct	2,11883	TamR4kd up vs TamR	2,72271	TamR7kd up vs TamR7ct
45017	235760_at	64324	NSD1	nuclear receptor bindir	1,50349	TamR1kd up vs TamR1ct	1,84947	TamR4kd up vs TamR	1,54427	TamR7kd up vs TamR7ct
12325	202869_at	4938	OAS1	2'-5'-oligoadenylate syn	2,20268	TamR1kd up vs TamR1ct	3,49767	TamR4kd up vs TamR	2,93312	TamR7kd up vs TamR7ct
15007	205552_s_at	4938	OAS1	2'-5'-oligoadenylate syn	2,38369	TamR1kd up vs TamR1ct	4,15708	TamR4kd up vs TamR	2,76033	TamR7kd up vs TamR7ct
15111	205656_at	27253	PCDH17	protocadherin 17	1,65409	TamR1kd up vs TamR1ct	1,56359	TamR4kd up vs TamR	1,90842	TamR7kd up vs TamR7ct
35567	226303_at	5239	PGM5	phosphoglucomutase 5	1,94628	TamR1kd up vs TamR1ct	1,52946	TamR4kd up vs TamR	1,54198	TamR7kd up vs TamR7ct
28306	219014_at	51316	PLAC8	placenta-specific 8	1,81576	TamR1kd up vs TamR1ct	1,69957	TamR4kd up vs TamR	1,76567	TamR7kd up vs TamR7ct
39343	230081_at	345557	PLCXD3	phosphatidylinositol-sp	1,76067	TamR1kd up vs TamR1ct	1,76849	TamR4kd up vs TamR	1,92761	TamR7kd up vs TamR7ct
13739	204284_at	5507	PPP1R3C	protein phosphatase 1,	1,60045	TamR1kd up vs TamR1ct	1,86915	TamR4kd up vs TamR	1,6617	TamR7kd up vs TamR7ct
51247	241990_at	171177	RHOV	ras homolog family mer	1,63935	TamR1kd up vs TamR1ct	2,26038	TamR4kd up vs TamR	1,60739	TamR7kd up vs TamR7ct
38664	229402_at	148418	SAMD13	sterile alpha motif dom	2,21936	TamR1kd up vs TamR1ct	1,75284	TamR4kd up vs TamR	1,63074	TamR7kd up vs TamR7ct
20767	211429_s_at	5265	SERPINA1	serpin peptidase inhibit	-1,98262	TamR1kd down vs TamR1ct	-2,43884	TamR4kd down vs Tar	-2,13831	TamR7kd down vs TamR7ct
12289	202833_s_at	5265	SERPINA1	serpin peptidase inhibit	-1,94639	TamR1kd down vs TamR1ct	-2,36479	TamR4kd down vs Tar	-2,22006	TamR7kd down vs TamR7ct
21753	212440_at	11017	SNRNP27	small nuclear ribonucle	-1,58958	TamR1kd down vs TamR1ct	-1,72807	TamR4kd down vs Tar	-1,56753	TamR7kd down vs TamR7ct
39500	230238_at	134548	SOWAHA	sosondowah ankyrin re	1,71477	TamR1kd up vs TamR1ct	2,02386	TamR4kd up vs TamR	1,89015	TamR7kd up vs TamR7ct
19178	209762_x_at	3431	SP110	SP110 nuclear body pro	1,9203	TamR1kd up vs TamR1ct	2,02084	TamR4kd up vs TamR	1,80889	TamR7kd up vs TamR7ct
18091	208667_s_at	6767	ST13	suppression of tumorig	-1,61183	TamR1kd down vs TamR1ct	-2,12167	TamR4kd down vs Tar	-1,78157	TamR7kd down vs TamR7ct
16379	206925_at	7903	ST8SIA4	ST8 alpha-N-acetyl-neur	1,52326	TamR1kd up vs TamR1ct	2,36684	TamR4kd up vs TamR	1,76268	TamR7kd up vs TamR7ct
52200	242943_at	7903	ST8SIA4	ST8 alpha-N-acetyl-neur	2,21396	TamR1kd up vs TamR1ct	2,62586	TamR4kd up vs TamR	2,12127	TamR7kd up vs TamR7ct
54641	AFFX-HUMISGF3	6772	STAT1	signal transducer and ac	1,50765	TamR1kd up vs TamR1ct	1,53937	TamR4kd up vs TamR	1,74689	TamR7kd up vs TamR7ct
19227	209813_x_at	445347	TARP	TCR gamma alternate re	2,83493	TamR1kd up vs TamR1ct	1,89306	TamR4kd up vs TamR	2,54744	TamR7kd up vs TamR7ct
26218	216920_s_at	6967 /// 44534	TARP /// TRGC2	TCR gamma alternate re	2,59803	TamR1kd up vs TamR1ct	2,04758	TamR4kd up vs TamR	2,61628	TamR7kd up vs TamR7ct
15398	205943_at	6999	TDO2	tryptophan 2,3-dioxyge	1,51784	TamR1kd up vs TamR1ct	1,79408	TamR4kd up vs TamR	2,10158	TamR7kd up vs TamR7ct
37383	228121_at	7042	TGFB2	transforming growth fa	2,38156	TamR1kd up vs TamR1ct	1,7205	TamR4kd up vs TamR	1,57747	TamR7kd up vs TamR7ct
12143	202687_s_at	8743	TNFSF10	tumor necrosis factor (l	2,1094	TamR1kd up vs TamR1ct	1,89422	TamR4kd up vs TamR	2,57615	TamR7kd up vs TamR7ct
36165	226902_at	8975	USP13	ubiquitin specific pepti	-1,69247	TamR1kd down vs TamR1ct	-1,99806	TamR4kd down vs Tar	-2,05152	TamR7kd down vs TamR7ct
36684	227421_at	54059	YBEY	ybeY metallopeptidase	1,94206	TamR1kd up vs TamR1ct	1,8674	TamR4kd up vs TamR	1,95276	TamR7kd up vs TamR7ct

Supplementary Table 5. Gene ontology term enrichment analysis of the 77 genes exhibiting altered expression upon MCM3 knockdown in TamR cell lines using Ingenuity Pathway analysis software. List of the molecular functions in which the genes showed significant enrichment and the corresponding genes involved in each molecular function as analyzed using Ingenuity Pathway analysis software.

Molecular function	p values	Genes
Cell Signaling	$1.4x10^{-3}$-$1.6x10^{-6}$	CCL5, IFIT1, ISG15, OAS1, STAT1, IFITM1
Cell Cycle	$3.3x10^{-3}$-$2.6x10^{-5}$	DOX58, Irf9, STAT1, CLU, EGR3, FZD8, OAS1, TGFB2, TNFSF10, DDX58, CDKN2B, TGFB2
Cell Death & Survival	$5.2x10^{-3}$-$1.5x10^{-5}$	CCL5, ENTPD, GBP1, ISG15, STAT1, TNFSF10, BLNK, CSTA, ENTPD1, IFIT3, MYBL, PLAC8, ST8SIA4, CADM1, DDX58, NDST4, SERPINA1, EGR3, SPI10, TDO2, CLU, EGR3, IFIT2, LRP2, OAS1, ST13, TGFB2, BCHE, DOCK8, ST8SIA4, IFI6, CAMK2B, NDST4, PLAC8

Supplementary Methods

Mass spectrometry-based proteomic analysis

Stable Isotope Labeling with Amino Acids of Cell Lines

MCF-7/S0.5 and TamR-1 cells were cultured separately in custom-made phenol red-free D-MEM/F-12 (1:1) medium without L-arginine, L-lysine and L-glutamine (1-57S10-I, Medinova Scientific A/S,) supplied with 1% dialyzed FBS (Hyclone), insulin (6ng/ml, Sigma, I6634) and $^{12}C_6$ L-glutamine (580mg/L, Sigma, G8540) at 37°C and 5% CO_2. Prior to the comparative quantitative proteome analysis, MCF-7/S0.5 and TamR-1 cell lines were labeled metabolically using stable isotope labeling with amino acids in cell culture (SILAC) (1). Medium for MCF-7/S0.5 cells was supplied with light amino acids $^{12}C_6$ L-arginine (22mg/L, Sigma, A-6969) and $^{12}C_6$ L-lysine (48mg/L, Sigma, L-8662). Medium for TamR-1 cells was supplied with heavy stable isotopes of the amino acids $^{13}C_6{}^{15}N_4$ L-arginine (23mg/L, Cambridge Isotope Laboratories, CNLM-539-0) and $^{13}C_6{}^{15}N_2$ L-lysine (59.5mg/L, Cambridge Isotope Laboratories, CNLM-291-0) and tamoxifen (10^{-6}M, T5648, Sigma). In addition, proline (8mg/L) was added to the medium to inhibit arginine conversion to proline (2). MCF-7/S0.5 and TamR-1 cells were propagated 4 times to achieve > 90% incorporation of the light and heavy isotopes. MCF-7/S0.5 and TamR-1 cells grown in standard medium were 85% confluent after one week when seeded into $1x10^5$/T25 flasks. Evaluation of growth showed that labeling the amino acids with isotopes and the dialyzed FBS had no influence on the growth rate of the cell lines.

Sample preparation for Mass Spectrometry Analysis and Western blotting

MCF-7/S0.5 and TamR-1 cells used for mass spectrometry analysis were harvested with trypsin and mixed 1:1. Cells were lysed in ice-cold hypotonic buffer (1M HEPES (pH 7.9), 1M $MgCl_2$, 2.5M KCL, 1M DTT) supplied with Mini EDTA-free complete protease inhibitor (04396159001, Roche,) for 5 min. Cells analyzed by mass spectrometry were homogenized by 20 strokes with a tight pestle using a pre-chilled Dounce homogenizer. The homogenate was centrifuged at 1000rpm for 5 min. at 4°C

to pellet nuclei and other fragments, and the supernatant was retained as the cytoplasmic fraction. Cells used for Western blotting were scraped off the flask in RIPA buffer (10mm Tris (pH 8.0), 5mm EDTA (pH 8.0), 0.1% Igepal, 0.5% sodium deoxycholate, 0.1% SDS). Protein concentrations were determined using a Pierce BCA protein assay kit (Thermo Scientific, 23225).

Nuclear and cytosolic fractions from SILAC-labeled cells were prepared as described above. The proteins in the cytosolic fraction were precipitated with ice-cold acetone. All fractions were dissolved in denaturant 6M/2M urea/thiourea, and supplemented with phosphatase inhibitors (sodium fluoride, β-glycerophosphate and pervanadate). Benzonase (Merck) was added to the nuclear fraction. All steps were performed at room temperature to avoid carbamoylation of amines. Reduction of cysteines was performed with DTT (5mM) for 30 min followed by alkylation with iodoacetamide (11mM) for 20 min in the dark. The proteins were digested 1:100 protease:protein with LysC (Wako) for 3.5 h, diluted 4 times with 50 mM ammonium bicarbonate followed by trypsin (Promega) digestion overnight. The nuclear fraction was spun at 10,000 rpm for 10 min and the supernatant was filtered through a 0.45μm filter (MillexHV Ø400). From each cellular fraction, approximately 100μg digested protein was collected for isoelectric focusing. The two digested fractions were desalted using SepPak C18 purification cartridges (Waters) and each was subsequently separated into 12 fractions by isoelectric focusing using the Agilent 3100 OFFGEL Fractionator (Agilent, G3100) following the protocol described previously (3). Gelstrips and amfolyte buffer were purchased from GE Healthcare. The peptides were focused for 20 kVh at a maximum current of 50 μA and maximum power of 200 mW**.** Trifluoroacetic acid (10μl of 10%) was added to each fraction and STAGE-tipped as described (4) prior to mass spectrometry (MS) analysis, processing and bioinformatics analysis of the proteomic data.

Isoelectric Focusing

The two digested fractions were desalted using SepPak C18 purification cartridges (Waters) and each was subsequently separated into 12 fractions by IEF using the Agilent 3100 OFFGEL

Fractionator (Agilent, G3100) following the protocol described previously (3). Gelstrips and amfolyte buffer were purchased from GE Healthcare. The peptides were focused for 20 kVh at a maximum current of 50 µA and maximum power of 200 mW.

Mass Spectrometric Analysis

MS analysis was performed on an LTQ-Orbitrap-Velos (5) (Thermo Fisher Scientific) connected to an Agilent 1100 nanoflow HPLC system (Agilent) using a nanoelectrospray ion source (Proxeon Biosystems). Mass spectrometry analysis of MCF-7/S0.5 and TamR-1 was performed twice. Peptides were separated by reverse-phase chromatography using an in-house fused silica emitter (75µm ID) packed with Reprosil-Pur C18-AQ 3µm reverse-phase material (Dr. Maisch GmbH). Peptides were loaded in 98% solvent A (0.5% acetic acid) followed by 100 min linear gradient to 50% solvent B (80% acetonitrile, 0.5% acetic acid). Survey full scan MS spectra (m/z range 300-2000, resolution 60.000@m/z 400) were acquired followed by fragmentation of the 20 most intense multiply-charged ions. Ions selected for MS/MS were placed on a dynamic exclusion list for 45 seconds. Real-time internal lock mass recalibration was used during data acquisition (6).

Processing of Proteomic Data

All raw files were processed with MaxQuant v. 1.0.13.13 (7) into centroided data and submitted to the database with Mascot v.2.2 (Matrix-Science). Pre-processing by MaxQuant was performed to determine charge states, miscleavages and SILAC states, and to filter the MS/MS spectra, keeping the 6 most intense peaks within a 100Da bin. Carbamidomethylation of Cystein was chosen as fixed modification, and acetylation of the protein N-terminus, oxidation of Methionine, Glutamine-to-pyroGlutamate conversion, Glutamate-to-pyroGlutamate conversion and deamidation of Asparagine

and Glutamine, were chosen as variable modifications. The processed MS/MS spectra were searched against a concatenated target-decoy database of forward and reverse sequences from the IPI database (152616 sequences, FASTA file created 20080506). For the search, Trypsin/P+DP (allows tryptic cleavage C-terminal to Lysine and Arginine unless Proline is C-terminal of these amino acids, and allows gas-phase-induced autocleavage between Aspartate and Proline) was chosen for the in silico protein digestion, allowing 3 miscleavages. The mass tolerance for the MS spectra acquired in the Orbitrap was set to 7 ppm, whereas the MS/MS tolerance was set to 0.6 Da for the CID MS/MS spectra from the LTQ.

Subsequent to the peptide search, protein and peptide identification were performed, including an estimated maximal false discovery rate (FDR) of 1% on both the protein and peptide levels. For the FDR calculation, posterior error probabilities (PEP) were calculated based on peptides of at least 6 amino acids having a MASCOT score of at least 7. For the protein group, identification of at least one unique peptide was required. The foundation of the PEP and FDR calculation was described previously (7).

For protein quantification, all modified versions of peptides were used. According to the protein group assignment performed by MaxQuant, both razor and unique peptides were used for protein quantitation. A minimum of two ratio counts is required for protein quantitation, and normalization was based on a logarithm-transformation algorithm, as described (7). The two cell lines were analyzed twice by mass spectrometry and comparison of the quantified proteins showed 96% correlation between the two data sets. The mass spectrometry proteomics data have been deposited to the ProteomeXchange Consortium via the PRIDE partner repository with the dataset identifier PXD001087.

Analysis of the proteomic data showed good incorporation of the labeled amino acids (94%) and demonstrated that most protein ratios were within the 1.5-fold margin, and approximately 50% were based on the median of 2-8 quantified peptides ratio associated to the protein (Supplemental Fig.

5A, B and C). Based on the 5% and 95% percentiles, manual inspection of spectra of proteins, the differential expression threshold was set at 1.5-fold.

Bioinformatic Analysis of Proteomic Data

Mass spectrometry-derived data from MaxQuant processing was subjected to analysis using the statistical environment R (RDevelopmentCoreTeam). A global functional association network was constructed using the probabilistic algorithm STRING (v 3.8) (8) based on identified proteins exhibiting altered expression by >1.5-fold. One intermediate protein predicted by STRING was allowed between two identified and differentially-expressed proteins to obtain a more comprehensive network for further analysis. To produce a network with high confidence, a STRING score of at least 0.7 was required. The Cytoscape (9) plugin MCODE (10) was used to extract significantly-connected sub-networks in the global constructed network. Default settings were applied for the MCODE analysis. KEGG pathway analysis was performed using DAVID (11) by applying hypergeometric enrichment testing (Fisher's Exact test) and subsequent adjustment for multiple testing using the standard Benjamini-Hochberg (BH) algorithm to obtain an adjusted *P*-value. The significance level was set to 0.05 after BH-adjustment. Finally, an enrichment score was defined as –log10(P).

Processing of Gene Microarray Datasets

Three gene microarray data sets of breast cancer cell line models were downloaded to study alterations in the MCM3 axis between cells cultured in estrogen-containing and long-term estrogen-deprived (LTED) medium, respectively. Microarray data sets at several time points for the LTED-cells were available. MCM3 levels in LTED-cells was correlated with the corresponding cells cultured in estrogen-containing medium (day 0). Datasets with the accession numbers GSE20361 (13), GSE38829 (14) and GSE50820 (15) were downloaded from the Gene Expression Omnibus database (16). The effect of MCM3 knockdown on TamR cells was investigated by gene array following siRNA transfections using Affymetrix Hu-U133 plus 2.0 arrays (Affymetrix). Genes showing

≥1.5-fold alteration in expression, false discovery rate (FDR) <0.05 cutoff and $p<0.01$ were considered to represent significantly altered expression in TamR between MCM3-specific siRNA and siControl. All data analyses were performed using Partek Genomic Suite (Partek, Inc., Chesterfield, MO, USA). Raw Affymetrix intensity measurements were background corrected, normalized and summarized into gene expression measurements using Robust Multiarray Average (RMA). Groups were compared using one-way ANOVA and a FDR of 0.05 was applied to identify differentially-regulated genes. Network and pathway analysis were performed using Ingenuity Pathway Analysis (IPA, QIAGEN, www.qiagen.com/ingenuity).

Western Blot Analysis

Protein lysates were prepared as described above. Equal concentrations of protein lysates were loaded on a 4-20% gel (Expedeon, Kem En Tec) and transferred onto a PVDF membrane. Membranes were blocked in TBS, 0.1% Tween-20, and 5% non-fat dry milk powder prior to primary antibody incubation. Antibody incubation and washing steps were carried out in TBS supplied with 0.1% Tween-20 and 1% non-fat dry milk powder or 1% BSA according to manufacturer's protocol. The immunoreactive bands were visualized using ECL Western Blot kit (Amersham Biosciences). Anti-β-actin or anti-GAPDH was included to ensure equal loading. The following antibodies were used: anti-β-actin (1:200.000, ab6276, Abcam), anti-MCM3 (1:1.000, 4012, Cell Signaling technology), anti-ER (1:500, clone SP1, Thermo Scientific), anti-bcl-2 (1:2.000, M0887, Dako), anti-ATR (1:1000, 2790, Cell Signaling technology), anti-JunB (1:1000, 3753, Cell Signaling technology), anti-IGF-1R (1:1000, 3027, Cell Signaling technology), anti-FOXO3a (1:1000, 2497, Cell Signaling technology), anti-AKT1 (1:1000, 2938, Cell Signaling technology), anti-CtIP (1:1000, Ab96773, Abcam), anti-GAPDH (1:10.000, 6C5, Santa Cruz biotechnology). HRP-conjugated goat anti-rabbit (P0448) or goat anti-mouse (P0447) (Dako). All blots derived from the same experiment were generated and processed in parallel.

Quantitative Real-Time PCR

Total RNA was purified using the Isol-RNA lysis reagent (733-1089, SPRIME). RevertAid Premium Reverse Transcriptase (EP0733), ribolock (EO0382), random hexamers (SO142) and dNTP mix (10mM) (R0181) (Fermentas) were used for cDNA synthesis. Relative quantification of gene expression was performed in triplicates using SYBR Green PCR Master mix (Applied Biosystems, Foster City, CA) according to manufacturer's instruction. Relative mRNA expression levels and fold-changes were normalized using the reference gene PUM1 and calculated using $2^{-\Delta\Delta Ct}$. The primers for specific gene amplification were from the QuantiTect Primer assays collection (Qiagen). MCM3: QT00030044, Reference primer: PUM1: QT00029421.

Phospho-Specific Cell Cycle Antibody Microarray Analysis

Phospho-specific cell cycle control antibody array (PCC238) and reagents (KAS02) were obtained from Full Moon Biosystems, Inc. Briefly, TamR-1 cells grown in the presence of 10^{-6}M tamoxifen were transfected with MCM3 targting siRNAs or siControl, as described above, and MCM3 levels were analyzed by Western blotting, as described above. At 96 h following transfection, lysates were prepared and applied on the antibody arrays according to the manufacturer's guidelines. Extraction buffer was supplied with phosphatase and protease inhibitor tablets (04693124001, 04906837001, Roche) and freshly prepared orthovanadate (2mM). Cell lysate (100 µg) in labeling buffer was labeled with 3µl biotin in N,N-Dimethylformamide (10µg/µl) for 2 h. Antibody microarray slides were placed in blocking solution, rinsed extensively in Milli-Q grade water and incubated with biotin-labeled proteins for 2 h. Bound biotin-labeled proteins were detected using Alexa fluor®647-streptavidin (S32357, Invitrogen). The microarrays were scanned and analyzed using Image Analysis Service provided by Full Moon Biosystems, Inc. Network and pathway analysis were performed using Ingenuity Pathway Analysis (IPA, QIAGEN, www.qiagen.com/ingenuity).

References

1. Ong S-E, and Mann M. A practical recipe for stable isotope labeling by amino acids in cell culture (SILAC). *Nat Protocols.* 2007;1(6):2650-60.
2. Lajoie GA, Bendall SC, Hughes C, Stewart MH, Doble B, and Bhatia M. Prevention of amino acid conversion in SILAC experiments with embryonic stem cells. *Molecular & Cellular Proteomics.* 2008;7(9):1587-97.
3. Hubner NC, Ren S, and Mann M. Peptide separation with immobilized pI strips is an attractive alternative to in-gel protein digestion for proteome analysis. *Proteomics.* 2008;8(23-24):4862-72.
4. Rappsilber J, Mann M, and Ishihama Y. Protocol for micro-purification, enrichment, pre-fractionation and storage of peptides for proteomics using StageTips. *Nature protocols.* 2007;2(8):1896-906.
5. Olsen JV, Schwartz JC, Griep-Raming J, Nielsen ML, Damoc E, Denisov E, Lange O, Remes P, Taylor D, Splendore M, et al. A dual pressure linear ion trap Orbitrap instrument with very high sequencing speed. *Molecular & cellular proteomics : MCP.* 2009;8(12):2759-69.
6. Olsen JV, de Godoy LM, Li G, Macek B, Mortensen P, Pesch R, Makarov A, Lange O, Horning S, and Mann M. Parts per million mass accuracy on an Orbitrap mass spectrometer via lock mass injection into a C-trap. *Mol Cell Proteomics.* 2005;4(12):2010-21.
7. Cox J, and Mann M. MaxQuant enables high peptide identification rates, individualized p.p.b.-range mass accuracies and proteome-wide protein quantification. *Nature biotechnology.* 2008;26(12):1367-72.
8. von Mering C, Jensen LJ, Snel B, Hooper SD, Krupp M, Foglierini M, Jouffre N, Huynen MA, and Bork P. STRING: known and predicted protein-protein associations, integrated and transferred across organisms. *Nucleic Acids Res.* 2005;33(Database issue):D433-7.
9. Shannon P, Markiel A, Ozier O, Baliga NS, Wang JT, Ramage D, Amin N, Schwikowski B, and Ideker T. Cytoscape: a software environment for integrated models of biomolecular interaction networks. *Genome Res.* 2003;13(11):2498-504.
10. Bader GD, and Hogue CW. An automated method for finding molecular complexes in large protein interaction networks. *BMC Bioinformatics.* 2003;4(2.
11. Huang da W, Sherman BT, Tan Q, Kir J, Liu D, Bryant D, Guo Y, Stephens R, Baseler MW, Lane HC, et al. DAVID Bioinformatics Resources: expanded annotation database and novel algorithms to better extract biology from large gene lists. *Nucleic acids research.* 2007;35(Web Server issue):W169-75.
12. Leth-Larsen R, Lund R, Hansen HV, Laenkholm AV, Tarin D, Jensen ON, and Ditzel HJ. Metastasis-related plasma membrane proteins of human breast cancer cells identified by comparative quantitative mass spectrometry. *Molecular & Cellular Proteomics.* 2009;8(6):1436-49.
13. Aguilar H, Sole X, Bonifaci N, Serra-Musach J, Islam A, Lopez-Bigas N, Mendez-Pertuz M, Beijersbergen RL, Lazaro C, Urruticoechea A, et al. Biological reprogramming in acquired resistance to endocrine therapy of breast cancer. *Oncogene.* 2010;29(45):6071-83.
14. Aguilar H, Urruticoechea A, Halonen P, Kiyotani K, Mushiroda T, Barril X, Serra-Musach J, Islam A, Caizzi L, Di Croce L, et al. VAV3 mediates resistance to breast cancer endocrine therapy. *Breast Cancer Res.* 2014;16(3):R53.
15. Milosevic J, Klinge J, Borg AL, Foukakis T, Bergh J, and Tobin NP. Clinical instability of breast cancer markers is reflected in long-term in vitro estrogen deprivation studies. *BMC Cancer.* 2013;13(473.
16. Barrett T, Troup DB, Wilhite SE, Ledoux P, Rudnev D, Evangelista C, Kim IF, Soboleva A, Tomashevsky M, and Edgar R. NCBI GEO: mining tens of millions of expression profiles--database and tools update. *Nucleic Acids Res.* 2007;35(Database issue):D760-5.
