# Supplementary material for: MCM3 upregulation confers endocrine resistance in breast cancer and is a predictive marker of diminished tamoxifen benefit
Source: NPJ Breast Cancer. 2021 Jan 4;7:2. doi: 10.1038/s41523-020-00210-8 (PMC7782683; doi:10.1038/s41523-020-00210-8)
Supplement: Supplementary file 2 — Reporting Summary Checklist [file 41523_2020_210_MOESM2_ESM.pdf]

nature research

Corresponding author(s): *Henrik Ditzel*

Last updated by author(s): *Nov 18, 2020*

Reporting Summary

Nature Research wishes to improve the reproducibility of the work that we publish. This form provides structure for consistency and transparency in reporting. For further information on Nature Research policies, see Authors & Referees and the Editorial Policy Checklist.

Statistics

For all statistical analyses, confirm that the following items are present in the figure legend, table legend, main text, or Methods section.

n/a	Confirmed	
☐	☒	The exact sample size (*n*) for each experimental group/condition, given as a discrete number and unit of measurement
☐	☒	A statement on whether measurements were taken from distinct samples or whether the same sample was measured repeatedly
☐	☒	The statistical test(s) used AND whether they are one- or two-sided<br>*Only common tests should be described solely by name; describe more complex techniques in the Methods section.*
☐	☒	A description of all covariates tested
☐	☒	A description of any assumptions or corrections, such as tests of normality and adjustment for multiple comparisons
☐	☒	A full description of the statistical parameters including central tendency (e.g. means) or other basic estimates (e.g. regression coefficient) AND variation (e.g. standard deviation) or associated estimates of uncertainty (e.g. confidence intervals)
☐	☒	For null hypothesis testing, the test statistic (e.g. *F*, *t*, *r*) with confidence intervals, effect sizes, degrees of freedom and *P* value noted<br>*Give P values as exact values whenever suitable.*
☒	☐	For Bayesian analysis, information on the choice of priors and Markov chain Monte Carlo settings
☐	☒	For hierarchical and complex designs, identification of the appropriate level for tests and full reporting of outcomes
☐	☒	Estimates of effect sizes (e.g. Cohen's *d*, Pearson's *r*), indicating how they were calculated

Our web collection on statistics for biologists contains articles on many of the points above.

Software and code

Policy information about availability of computer code

Data collection	kmplot.com, MaxQuant v. 1.0.13.13,
Data analysis	Mascot v.2.2 (Matrix-Science), RDevelopmentCoreTeam, GraphPad Prism, STATISTICA 10 (StatSoft Inc.), STRING, Cytoscape plugin MCODE, DAVID, Partek Genomic Suite, Robust Multiarray Average (RMA).

For manuscripts utilizing custom algorithms or software that are central to the research but not yet described in published literature, software must be made available to editors/reviewers. We strongly encourage code deposition in a community repository (e.g. GitHub). See the Nature Research guidelines for submitting code & software for further information.

Data

Policy information about availability of data

All manuscripts must include a data availability statement. This statement should provide the following information, where applicable:

- Accession codes, unique identifiers, or web links for publicly available datasets
- A list of figures that have associated raw data
- A description of any restrictions on data availability

The data generated and analyzed during this study are described in the following data record (to be included in the final version). The mass spectrometry proteomics data have been deposited to the ProteomeXchange Consortium via the PRIDE partner repository with the dataset identifier PXD001087. The data is currently private, but can be accessed by the reviewers through the account created. Reviewer account: - Username: reviewer55145@ebi.ac.uk Password: xUVzn7WQ, - To access the data please visit: http://tinyurl.com/o2vjeny . Microarray data were deposited and are accessible from the Gene Expression Omnibus (GEO) database [GEO:GSE74391]. The other data supporting the conclusions of this article are included within the article. The clinical data supporting the figures and tables in this published article are not publicly available to protect patient privacy, but can be accessed from the corresponding author on reasonable request. Data will be made available from the Department of Cancer and Inflammation Research at University of Southern Denmark to authorized researchers who have an approved institutional IRB application and have obtained approval from The Regional Committees on Health Research Ethics for Southern Denmark (Vejle, Denmark).

Field-specific reporting

Please select the one below that is the best fit for your research. If you are not sure, read the appropriate sections before making your selection.

☒ Life sciences ☐ Behavioural & social sciences ☐ Ecological, evolutionary & environmental sciences

For a reference copy of the document with all sections, see nature.com/documents/nr-reporting-summary-flat.pdf

Life sciences study design

All studies must disclose on these points even when the disclosure is negative.

Sample size	Power calculations were performed to determine that the number of animals and tumor samples should be sufficient to determine significant differences between the different groups.
Data exclusions	No data was excluded.
Replication	All experiments were performed at least twice, but more often three times.
Randomization	When the FulvR-1 and MPF-R tumor xenografts reached 100-150 mm3, the mice were randomized into treatment groups based on equal tumor sizes.
Blinding	Evaluation of the immunohistochemistry staining was performed by a skilled breast pathologist in a blinded setup.

Reporting for specific materials, systems and methods

We require information from authors about some types of materials, experimental systems and methods used in many studies. Here, indicate whether each material, system or method listed is relevant to your study. If you are not sure if a list item applies to your research, read the appropriate section before selecting a response.

Materials & experimental systems

n/a	Involved in the study	
☐	☒	Antibodies
☐	☒	Eukaryotic cell lines
☒	☐	Palaeontology
☐	☒	Animals and other organisms
☐	☒	Human research participants
☐	☒	Clinical data

Methods

n/a	Involved in the study	
☒	☐	ChIP-seq
☒	☐	Flow cytometry
☒	☐	MRI-based neuroimaging

Antibodies

Antibodies used	anti-β-actin (Abcam, #ab6276, clone AC-15), anti-MCM3 (Cell Signaling technology, #4012, polyclonal), anti-ER (Thermo Scientific, #MA5-14501, clone SP1), anti-bcl-2 (Dako, #M0887, clone 124), anti-ATR (Cell Signaling technology, #2790, polyclonal), anti-JunB (Cell Signaling technology, #3753, clone C37F9), anti-IGF-1R (Cell Signaling technology, #3027, polyclonal), anti-FOXO3a (Cell Signaling technology, #2497, clone 75D8), anti-AKT1 (Cell Signaling technology, #2938, clone C73H10), anti-CtIP (Abcam, #Ab96773, polyclonal), anti-GAPDH (Santa Cruz biotechnology, #sc-32233, clone 6C5), anti-MCM3 (Atlas Antibodies, #HPA004789, polyclonal), anti-AURKA (Sigma-Aldrich, #HPA002636, polyclonal).
Validation	All antibodies are commercially available and have been validated by the company for Western blotting and/or immunohistochemistry.

Eukaryotic cell lines

Policy information about cell lines

Cell line source(s)	The original MCF-7 and T47D cell lines were obtained from the Breast Cancer Task Force Cell Culture Bank, Mason Research Institute.
Authentication	All cell lines were authenticated by Short Tandem Repeat (STR) DNA profiling.
Mycoplasma contamination	All cell lines were tested negative for mycoplasma contamination using Lonza MycoAlert Kit.
Commonly misidentified lines (See ICLAC register)	N/A

Animals and other organisms

Policy information about studies involving animals; ARRIVE guidelines recommended for reporting animal research

Laboratory animals	The recipients were 7-week-old female NOG CIEA mice, which were purchased from Taconic.
Wild animals	N/A
Field-collected samples	N/A
Ethics oversight	All animal experiments were approved by the Experimental Animal Committee of The Danish Ministry of Justice and were performed at the animal core facility at University of Southern Denmark.

Note that full information on the approval of the study protocol must also be provided in the manuscript.

Human research participants

Policy information about studies involving human research participants

Population characteristics	The first cohort consisted of ER+ primary breast cancer tissues from 79 patients collected from Herlev and Roskilde Hospitals, Denmark. Of these 68 had sufficient tumor for staining. All tumors were ERα+ (>10%), 54% had tumor sizes > 20 mm, and 92% of the patients were lymph node-positive. These patients were part of a nationwide study of 1,115 Danish postmenopausal early-stage ER+ breast cancer patients who received 20 mg adjuvant tamoxifen daily for 5 years following radical surgery between 1995 and 2006. Eight-ten patients were excluded due to lack of tissue, leaving a total of 68 evaluable samples. The second cohort was extracted from a retrospective cohort of 589 patients from the Danish Breast Cancer Co-operative Group (DBCG) 89C randomized study. The selected patients consisted of 218 post- or peri-menopausal patients who, between 1989 and 2001, had lumpectomy or mastectomy at Odense Hospital. These patients received adjuvant tamoxifen mono-therapy for 0.5, 1, 2, or 5 years. Inclusion criteria included one of the following: positive axillary lymph nodes, tumor size > 50 mm (> 20 mm since 1999) and/or ductal grade II – III (since 1999), and therefore defined as high-risk patients. An additional criterion for inclusion was age at surgery less than 75 years. Patients were selected on the basis of the availability of concurrent fresh-frozen tumor tissue and positive hormone receptor status (ERα or PGR). None of the cancer patients included in the cohorts had received adjuvant cytotoxic therapy, treatment with AIs or neo-adjuvant endocrine treatment. The third cohort of 2555 breast cancer patients of which 2051 were ER+ (1802 were endocrine treated and 503 did not receive any systemic treatment). The remaining 250 were ER- patients. All data for cohort 3 were obtained from www.kmplot.com database, a net-based survival analysis tool where microarray data from over 4000 breast cancer patients was curetted and made publicly available (kmplot.com)14. The fourth cohort originated from the Stockholm Breast Cancer Study Group randomised tamoxifen STO-3 trial 1976-1990. A cohort of 1,780 postmenopausal women with breast cancer was randomised to adjuvant tamoxifen for 2 or 5 years (n = 886), or no adjuvant endocrine therapy (n = 894). The patients were all postmenopausal at the time of diagnosis and had a tumour size ≤ 30 mm (76% ≤ 20 mm) and lymph node-negative (N0), thus defined as low-risk patients. The patients had been treated with modified radical mastectomy (n = 1348) or breast-conserving surgery plus radiation therapy (n = 432). Since the treatment predictive value of hormone receptor status in the adjuvant setting was not certain at the time of the trial, no selection on the basis of hormone receptor status was made. The original trial has been described in detail previously.15 TMA was originally generated from primary tumors of 910 of these patients, and sufficient tumor tissue of 683 patients remained available for MCM3 assessment. The clinicopathological characteristics in this subset were similar to those in the complete series of 1,780 patients, such as a tumor size ≤20 mm (76% vs. 78%), ER-positive status (78% vs. 80%) and tamoxifen treatment (51% vs. 50%). The tumors were graded retrospectively according to the Nottingham system (NHG) by one pathologist blinded to clinical outcome. The study was approved by the local ethical committee at Karolinska Institute, Stockholm, Sweden. The biomarker study was conducted according to the REMARK recommendations.51 FFPE metastatic lesions from ER+ breast cancer patients treated with combined CDK4/6 inhibitor and endocrine therapy in the advanced setting were selected retrospectively by database extraction from the archives of the Department of Pathology at Odense University Hospital (n = 115). ER+ breast cancer patients treated with combined CDK4/6 inhibitor and endocrine therapy in the advanced setting who had undergone surgery or biopsy for advanced stage disease at Odense University Hospital were included. Samples were excluded if there was insufficient tumor material in the FFPE block or if the metastatic biopsy was only available after treatment with combined CDK4/6 inhibitor and endocrine therapy. These parameters yielded n = 86 patients.
Recruitment	For cohorts 1 and 2, clinical data on post-surgical patients were retrieved from the DBCG registry and by record linkage to the Danish Central Population Registry (date of death). For the first cohort, recurrence-free- (RFS) and overall survival (OS) were defined as the time from surgery to recurrence or death within 10 years, respectively. Patients without recurrence/death were censored at date of emigration, 10 years after the date of surgery or to the date of clinical database retrieval from the national registry (July 1, 2011), whichever came first. For the second cohort, RFS was defined as the time from surgery to the date of recurrence within 10 years. Exclusion criteria were: recurrence < 3 months of surgery, any treatment with AIs, bilateral breast cancer, and secondary primary cancers. OS were defined as the time from surgery to death within 10 years regardless of cause or end of follow-up. Patients who were recurrence-free or alive at 10-years follow-up were censored at date of emigration, 10 years after surgery, or on the date of clinical database retrieval from DBCG (June 6, 2012), whichever came first. With regard to OS, no patients were lost to follow-up due to database linkage to the Danish Civil Registration System. For the third cohort, follow-up data was obtained from a public database in kmplotter.com that was collected and curated for use in survival analysis. For the 4th cohort, follow-up data was collected from regional population registers and the Swedish Cause of Death Registry and the median follow-up period for the patients was 17 years. Recurrence-free survival (RFS) and breast cancer-specific survival (BCSS) were chosen as primary endpoints. For the cohort of ER+ advanced breast cancer patients treated with CDK4/6 inhibitor, progression-free survival (PFS) was chosen as endpoint and defined as the time from initiation of combined endocrine therapy and CDK4/6 inhibitor treatment until disease progression or death.
Ethics oversight	All clinical samples were coded to maintain patient confidentiality and the studies were approved by the Ethics Committee of the

Ethics oversight	Region of Southern Denmark and Copenhagen and Frederiksberg Counties (approval no. S-20080115, S-20170154, and 01025-KF12-138-99) and the Danish Data Protection Agency (2008-58-0035).

Note that full information on the approval of the study protocol must also be provided in the manuscript.

Clinical data

Policy information about clinical studies

All manuscripts should comply with the ICMJE guidelines for publication of clinical research and a completed CONSORT checklist must be included with all submissions.

Clinical trial registration	N/A
Study protocol	N/A
Data collection	N/A
Outcomes	N/A
